# Applying a Trauma-Informed Lens to Challenging Adolescent Encounters: A Faculty Development Session for Pediatricians

**DOI:** 10.15766/mep_2374-8265.11408

**Published:** 2024-05-31

**Authors:** Deborah Hall, Yael Smiley, Ariella Slovin, Jaytoya Manget, James E. Bost, Binny Chokshi

**Affiliations:** 1 Assistant Professor, Department of Pediatrics, The George Washington University School of Medicine and Health Sciences; 2 Instructor, Department of Pediatrics, The George Washington University School of Medicine and Health Sciences; 3 Research Division Chief, Center for Translational Research, Children's National Hospital; Associate Professor, Department of Pediatrics, The George Washington University School of Medicine and Health Sciences; 4 Associate Professor, Division of Military Child and Family Research, Department of Pediatrics, Uniformed Services University of the Health Sciences F. Edward Hébert School of Medicine

**Keywords:** Adverse Childhood Experiences, Trauma-Informed Care, Adolescent Medicine, Case-Based Learning, Communication Skills, Faculty Development, Pediatrics, Primary Care, Well-Being/Mental Health, Editor's Choice

## Abstract

**Introduction:**

Patient encounters perceived to be challenging are common and contribute to both suboptimal patient health outcomes and provider burnout. A trauma-informed care (TIC) approach to these encounters is critical, as many of the characteristics associated with challenging patient encounters can be linked to a history of trauma exposure.

**Methods:**

Our team created and delivered a 1-hour synchronous virtual session intended to bolster provider knowledge of TIC principles and their application to challenging adolescent encounters. Participants were all faculty and staff engaged in pediatric primary care at an urban academic center, including physicians, nurse practitioners, psychologists, and social workers. The content was rooted in adult learning principles and included didactic components anchored to case-based learning with facilitated group discussions and opportunities for reflection. We used paired pre- and postsession self-assessments of provider knowledge, confidence, and practice related to TIC using Likert-scale and free-text questions. Descriptive statistics and a paired *t* test were used to determine the impact of the session on these metrics.

**Results:**

In 24 paired surveys, there were statistically significant increases (*p* ≤ .001) in participant perceived knowledge, confidence, and practice, with 100% of participants having a statistically significant improvement in one or more of these domains. There were also strongly positive Likert-scale and free-text responses regarding content relevance and delivery.

**Discussion:**

We demonstrate that a brief session can create improvement in pediatric providers’ perceived knowledge about the application of TIC principles to challenging adolescent encounters as well as confidence in their ability to put these into practice.

## Educational Objectives

By the end of this activity, learners will be able to:

1. Describe the association between traumatic exposures and health outcomes.
2. Demonstrate how a trauma-informed lens can be applied to challenging patient encounters.
3. Discuss how to utilize trauma-informed practice in challenging patient encounters.

## Introduction

Studies have consistently shown that clinicians perceive up to 15% of patient encounters as difficult.^1^ Difficult patients or challenging clinical encounters are those in which the provider may have difficulty forming a therapeutic relationship with the patient.^2^ The reasons for this can be multifactorial. Previous studies have highlighted that patients are more likely to be labeled as difficult if they are nonadherent to treatment recommendations, have underlying mental health disorders, are high utilizers of health care services, have chronic health issues, or have greater symptom severity.^1,3^ The potential implications of a difficult patient interaction can include anxiety, concern, frustration, and dissatisfaction for the patient, along with similar emotions in the clinician, leading to clinician disengagement and potential burnout.^4,5^ These factors can contribute to the loss of a trusting patient-physician relationship and suboptimal patient outcomes.^6^

Given these potential outcomes, there is a need for formal training opportunities for health care providers on how to approach difficult patient interactions. For example, Collins and colleagues identified a need for training among pediatric residents who reported experiencing difficult patient encounters frequently and expressed a desire to learn how to manage these encounters, preferably using clinical experiences.^7^ In our review of the literature, we found a small number of existing curricula focused on this topic; one resource uses standardized patient scenarios to teach medical students strategies to care for adults who may display medication-seeking behaviors,^8^ while another introduces concepts utilized in mediation to reframe difficult patient interactions by approaching them with empathy and focusing on the underlying reasons for the conflict.^9^ Although these workshops present useful skills to apply to difficult patient encounters, they do not help clinicians recognize the causes of difficult behaviors or apply a holistic, trauma-informed approach to building relationships with patients.

Trauma-informed care (TIC) is a framework that involves understanding the prevalence of trauma and adversity, recognizing the impact of traumatic exposures on health and behaviors, and responding to patients and families with this perspective in mind, ultimately to avoid retraumatization and promote health and wellness.^10^ Ashana, Lewis, and Hart highlighted that the adaptive coping responses that may be rooted in a trauma response can be identified by health care providers as disruptive, leading patients and families to be labeled as difficult.^11^ In addition, patient characteristics and diagnoses frequently cited by physicians as difficult have been known to be linked to an interpersonal history of trauma.^12–14^ Underscoring this, Strous, Ulman, and Kotler noted that a physician's ability and openness to obtain a holistic picture of a patient ultimately assist with improved medical care, improved satisfaction for both physician and patient, and improved physician well-being.^15^

Applying a trauma-informed approach to challenging patient encounters provides clinicians with a framework to employ TIC's central tenet, which is “Trauma-informed care shifts the focus from ‘*What's wrong with you?’ to ‘What happened to you?’*”^16^ This allows clinicians to recognize trauma as pervasive and with myriad impacts, leading to cognitive, social, and physical effects, which together can impact an individual's interaction with the health care setting.^10,11,17^ TIC highlights that diagnoses often perceived by health care providers as challenging may themselves be rooted in a past history of trauma. TIC also helps to reframe difficult patient behaviors as adaptive mechanisms used to cope with powerlessness, uncertainty, and isolation in health care settings.

Hardavella and colleagues made recommendations for managing difficult patient encounters, including ensuring safety and prioritizing trust and communication.^6^ Specific to pediatrics, Breuner and Moreno offered communication strategies that can help with difficult encounters, including improved listening, partnering with patients, and increased empathy.^18^ Pluhar, Power, Freizinger, and Altman developed guidelines to manage challenging patient encounters based on a workshop they develised for medical students, including strategies such as nonjudgment, validation, and respect.^19^ However, these resources are not explicitly designed to teach TIC and lack a clear link defining the association between trauma, health outcomes, and difficult patient behaviors.

*MedEdPORTAL* has published numerous curricular resources related to the provision of TIC.^20–24^ While these resources are comprehensive in giving an overview of the principles of TIC and their application in broad settings, they do not include discussion of the use of TIC in the approach to challenging patient encounters.

Given these gaps, we created a session for pediatric practitioners focusing on adolescent patients and explicitly applying a trauma-informed approach to challenging patient encounters. Adolescents were chosen as the subset of pediatric patients for several reasons. We routinely provide care to adolescent patients and recognized through our firsthand experiences the necessity of training in TIC. In addition, within the subset of pediatrics, adolescents are likely to present with primary diagnoses, chief complaints, and behaviors that are often perceived as difficult by clinicians, such as mental health disorders, chronic pain symptoms, somatization, and noncompliance with treatment plans.^25^ Lastly, the development and maintenance of trust in an adolescent patient-clinician encounter are paramount when approaching sensitive topics such as sexual and reproductive health. Therefore, strategies to encourage and foster this relationship are necessary.

## Methods

### Content Development

Our team included a TIC expert (Binny Chokshi) who created the first draft of the session content based on a literature review. Content was developed based on the Substance Abuse and Mental Health Services Administration trauma-informed approach and six guiding principles,^10^ a review of existing education models for TIC and challenging patient encounters, and discussion with content experts through the National Collaborative on Trauma-Informed Care Education and Research, of which the study team senior author (Binny Chokshi) is a member. No baseline knowledge requirement was set, and the content was intended for participants with any level of exposure to the topics of adverse childhood experiences and the effects of trauma on various health and socioeconomic outcomes. The educational approach was rooted in adult learning principles, with a prioritization of participant interaction via audience response, small-group discussion, and reflection. All materials were reviewed for content and clarity by the study team, who collectively had expertise in pediatrics, TIC, primary care for vulnerable pediatric populations, care for parenting and expectant teens, medical education, program evaluation, and quality improvement. The team's members included institutional leaders in medical education and advocacy and had an average of 10 years of clinical experience.

### Content Delivery

We delivered a 1-hour, interactive, synchronous, virtual session attended by general pediatric clinical faculty and staff at Children's National Hospital. The session was delivered during division-wide protected professional development time. Participation in these sessions was strongly encouraged, but not required, and the sessions were open to all clinical team members, including physicians, nurse practitioners, administrators, psychologists, and social workers.

The session consisted of didactic components, case-based learning, and facilitated group discussions (see Appendix A, facilitator guide, and Appendix B, slide set). It began with a didactic presentation that reviewed trauma and adverse childhood experiences, including epidemiology and impacts on physiology, behavior, and health outcomes. We then introduced the concepts of TIC, including the central tenet of TIC and how to apply a trauma-informed lens to patient encounters. To illustrate these principles and facilitate participant application of the content, we utilized case-based learning with three scenarios commonly encountered with our adolescent patients and including components that providers typically find difficult.^1,3^ Utilizing specific prompts, we invited and facilitated discussion of participants’ experiences with similar cases in their clinics and highlighted how these encounters might reflect the effects of traumatic experiences in the lives of patients and families. The case-based discussions were followed by a didactic section providing in-depth review of the six principles of TIC and examples of practical application of each. The final case-based component of the session was a “Putting It Into Practice” review of the cases previously discussed to facilitate participant discussion of how the concepts could be applied in their practice.

Keeping with the current organizational practice for these professional development sessions, the session was conducted virtually using the Zoom online platform, which allowed for maximum participation despite varying geographic locations of participants. The session was facilitated by two presenters (Deborah Hall, Binny Chokshi) who delivered the didactic content and moderated the large-group discussions. An audience response interactive platform (PollEverywhere) allowed participants to respond to various prompts during the module, with the responses shared on-screen in real time to promote active discussion and interaction among the participants.

### Evaluation Methods

To evaluate the effectiveness of the session, each participant was invited to complete an 11-question presession survey asking for self-assessment of knowledge (five items), practice (four items), and confidence (two items) regarding the use of trauma-informed principles in challenging patient encounters. Each of the survey questions mapped to one or more of the stated objectives for the session. Following the presentation, participants were asked to complete a postsession survey that included the same self-assessment Likert-scale questions in addition to open-ended queries about key takeaway items and potential practice changes (Appendix C). A unique identifier was created by survey participants to allow linking of pre- and postsession survey responses. Information about participant clinical role, level of training, and practice setting was solicited, but no personal demographic information was collected. The Children's National Hospital Institutional Review Board reviewed the evaluation protocol and found it to be exempt.

Our team included a trained biostatistician and psychometrician (James E. Bost) specializing in building and analyzing patient-centered outcome measures, particularly survey data, who reviewed results and performed data analysis. We utilized descriptive statistics to highlight means and standard deviations across the full set of the pre- and postsession surveys. We used a paired *t* test to determine statistically significant differences through the generation of *p* values and 95% confidence intervals for mean differences in scores between pre- and postsession responses. We calculated perceived knowledge, practice, and confidence overall scores by taking the mean of the items included in each of these domains. We determined an overall total score by calculating the mean across all the items.

## Results

Our team delivered the session to 53 attendees, whose clinical roles are delineated in Table 1. Overall, 38 participants completed the presession survey (response rate: 72%), and 26 participants completed the postsession survey (response rate: 49%), with 24 paired pre- and postsession surveys (63% of presession responders, 45% of all participants; Table 1).

**Table 1.**
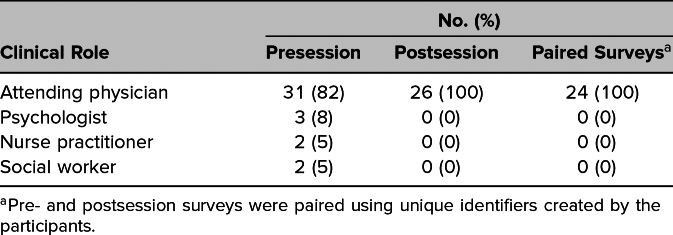
Clinical Roles of Survey Respondents

We observed a statistically significant improvement (*p* ≤ .001) in postsession survey Likert scores for the self-assessment of knowledge, practice, and confidence (Table 2). There were overall domain score increases of 1.5 points (95% CI, 1.1–2.9) for provider confidence and 1.5 points (95% CI, 1.1–1.9) for provider practices. As shown in Table 2, the most significant improvements were in the scores for provider practices and confidence, with paired responses showing an improvement of more than 1 point for all six items (range: 1.2–1.7). Perceived knowledge also increased for all items assessed, with the largest increases for items assessing concepts of TIC and smaller but still statistically significant increases on the two items assessing general knowledge of the effects of trauma on health outcomes and coping behaviors (Table 2).

**Table 2.**
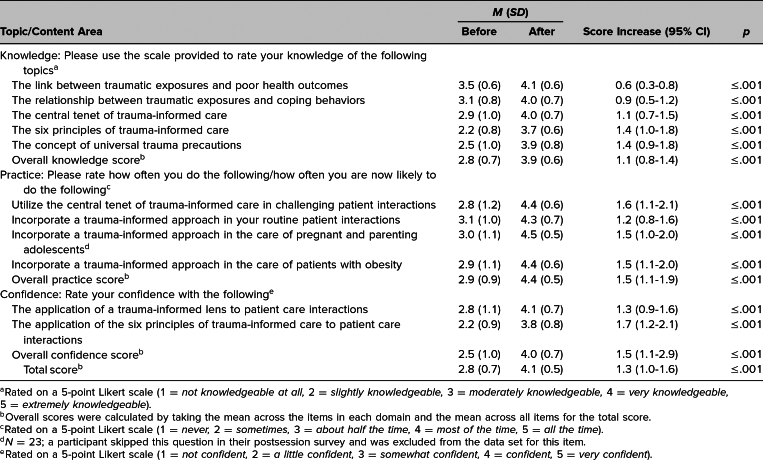
Mean Scores and Score Differences in Paired Pre- and Postsession Surveys (*N* = 24)

**Figure 1.**
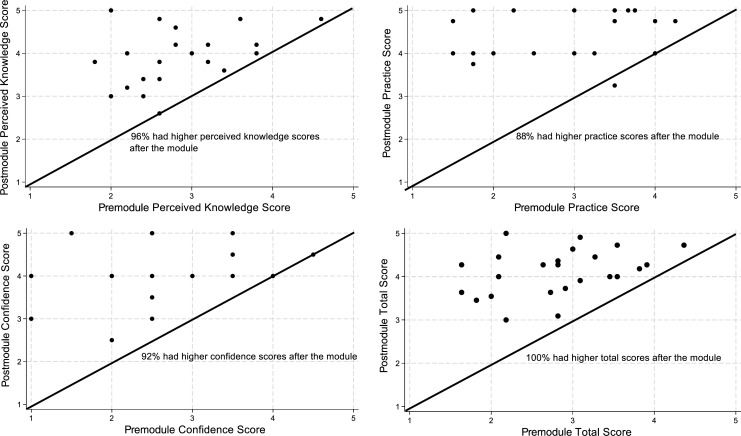
Scatter plots of pre- and postmodule domain and total scores. Dots above the 45-degree line represent improvement in scores. Dots can represent more than one participant (*N* = 24).

Figure 1 shows the number of respondents whose perceived knowledge, practice, confidence, and total scores improved from pre- to postsession surveys (indicated by dots above the 45-degree lines). We looked at changes in overall scores for each category as well as changes in total scores. The percentage of participants with improvements ranged from 88% to 100%, with the highest percentages associated with perceived knowledge (96%) and total scores (100%).

Participation in the case-based discussions was robust, with more than 20 free-text responses logged in the virtual poll for each case in addition to answers provided by participants in the session using the chat feature in the online platform or calling out answers during discussion. On the postsession survey, participants highlighted that the opportunity to actively participate during the session enhanced their learning. Although responses to this optional free-text response question were limited, five of seven respondents indicated that the case examples and discussions were the most helpful for them. Suggestions for improvement included additional time for practical applications and specific examples of trauma-informed language that participants could apply to challenging patient encounters.

**Figure 2.**
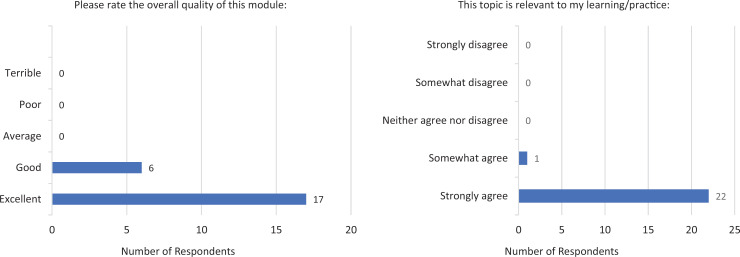
Postsession assessments of module quality and relevance (*N* = 23). Quality: minimum = 4.00, maximum = 5.00, *M* = 4.74, *SD* = 0.44, variance = 0.19. Relevance: minimum = 4.00, maximum = 5.00, *M* = 4.96, *SD* = 0.20, variance = 0.04.

Overall evaluation by postsession survey participants was strongly positive (Figure 2). On a 5-point Likert-scale rating of the overall quality of the session (1 = *terrible,* 5 = *excellent*), the mean score was 4.74 (*SD* = 0.44), and for relevance of the material to participant learning and practice (1 = *strongly disagree,* 5 = *strongly agree*), the mean score was 4.96 (*SD* = 0.20). In both categories, 100% of responses (*n* = 23) were at least 4 out of 5.

## Discussion

The goal of this session is to provide pediatricians with both the knowledge and skills to apply a trauma-informed approach to challenging patient encounters. The content is unique in its review of the central tenet of TIC, urging pediatricians to recognize that challenging patient behaviors and diagnoses may be related to a history of trauma and highlighting that the application of TIC can be useful in patient encounters perceived as challenging.

Our evaluation demonstrated that participation in the session increased participant perceived knowledge, confidence, and intended practices with respect to the application of trauma-informed principles to challenging patient encounters. This session provides an opportunity to train pediatricians efficiently given its 1-hour length and ability to be delivered virtually. The delivery can alternatively be easily adapted for an in-person audience, and suggestions for adaptation are included in Appendix A. It is important to note that our success was predicated on obtaining the buy-in of primary care leadership at our institution, which allowed us to deliver the session to 50+ participants during protected faculty development time.

In our postsession survey, we included a free-text question asking participants to provide suggestions for improvement. One limitation identified was lack of content acknowledging the role of provider biases or reactions to patient behaviors that might interfere with application of TIC principles. The focus of the session is on encouraging pediatricians to obtain a holistic view of patient presentations in order to recognize that challenging behaviors may be rooted in a history of trauma, thereby urging them to apply the principles of TIC in these patient encounters. However, a reflection on individual biases and reactions to a situation is a feature of a trauma-informed approach^26^ and can be the first necessary step before engaging in a challenging patient encounter, as an individual's implicit bias may directly impact their perception of a difficult encounter.^27^ We have included an additional slide at the end of the PowerPoint (Appendix B) should future users of this resource want to begin to incorporate this content. Future iterations of our session may further explore the relationship between implicit bias and difficult patient encounters by highlighting how individuals from racial and ethnic minorities can be disproportionately exposed to traumatic events,^28^ thereby underscoring the impact that a TIC approach can have in these patient encounters. Participants also asked for more concrete examples of action steps or language they could use during encounters they find challenging. We have included content to address this in the speaker notes for each of the cases in the “Putting It Into Practice” section of the module.

There are some additional limitations that we consider important to note. First, the nature of the online asynchronous completion of the surveys likely led to lower response rates than would have been achieved in person. We had 24 paired survey responses, all provided by physicians, representing only 45% of all participants. While this provided sufficient data for valid analysis, we cannot know if our findings would generalize to the remaining participants. The immediate postsession survey asked participants to provide self-assessment of changes in knowledge and to anticipate how their practices would change. Future research may consider more direct assessments of knowledge as well as a 3- to 6-month follow-up analysis to discern any impact on clinician behaviors. Lastly, our patient cases focus on adolescent patients, which may limit generalizability of our resource. However, the application of trauma-informed principles can be beneficial across patient encounters. In future iterations, we may include nonadolescent pediatric cases, and we encourage other users of this material to do so as well if appropriate to their patient care settings.

Regarding delivery and logistics, our team decided to keep the group together for the entire session and to employ multiple modes of participant interaction for the case reviews and group discussions. We did this to maximize time for discussion and to avoid loss of participants at transition points in a virtual setting. In an in-person setting, it would be preferable to incorporate small-group breakout components coupled with a report to the larger group to maximize participation, particularly in settings where participants are not familiar with each other. We provide suggestions for how this could be done in our facilitator guide (Appendix A).

Our results demonstrate that a single brief session can lead to improvement in pediatric providers’ self-assessment of knowledge about the application of TIC principles to challenging adolescent encounters as well as in their confidence in their ability to put the principles into practice. We hope that the content of this session helps to reframe patient behaviors and diagnoses, perhaps preventing an encounter from being perceived as challenging. In addition, we recognize that our session may work well in conjunction with other skill-based curricula to approach challenging patient encounters.^7^

Potential future directions for this work include adding content that encourages more self-reflection on provider biases or reactions to challenging patient encounters as well as expanding the delivery of this training to other health care staff, such as nurses and front-desk staff. To offer training to those audiences, we recommend including individuals in those roles as part of the team developing and delivering the content to ensure that the focus is relevant and addresses the challenges faced by those team members in their roles.

## Appendices


Facilitator Guide.docxModule Slide Set.pptxPre- and Postsession Survey.docx
*All appendices are peer reviewed as integral parts of the Original Publication.*
