# Supplementary material for: Applying a Trauma-Informed Lens to Challenging Adolescent Encounters: A Faculty Development Session for Pediatricians
Source: MedEdPORTAL. 2024 May 31;20:11408. doi: 10.15766/mep_2374-8265.11408 (PMC11219089; doi:10.15766/mep_2374-8265.11408)
Supplement: Supplementary file 1 — Facilitator Guide.docxModule Slide Set.pptxPre- and Postsession Survey.docx [file mep_2374-8265.11408-s001.zip › B. Module Slide Set.pptx]

## Slide 1
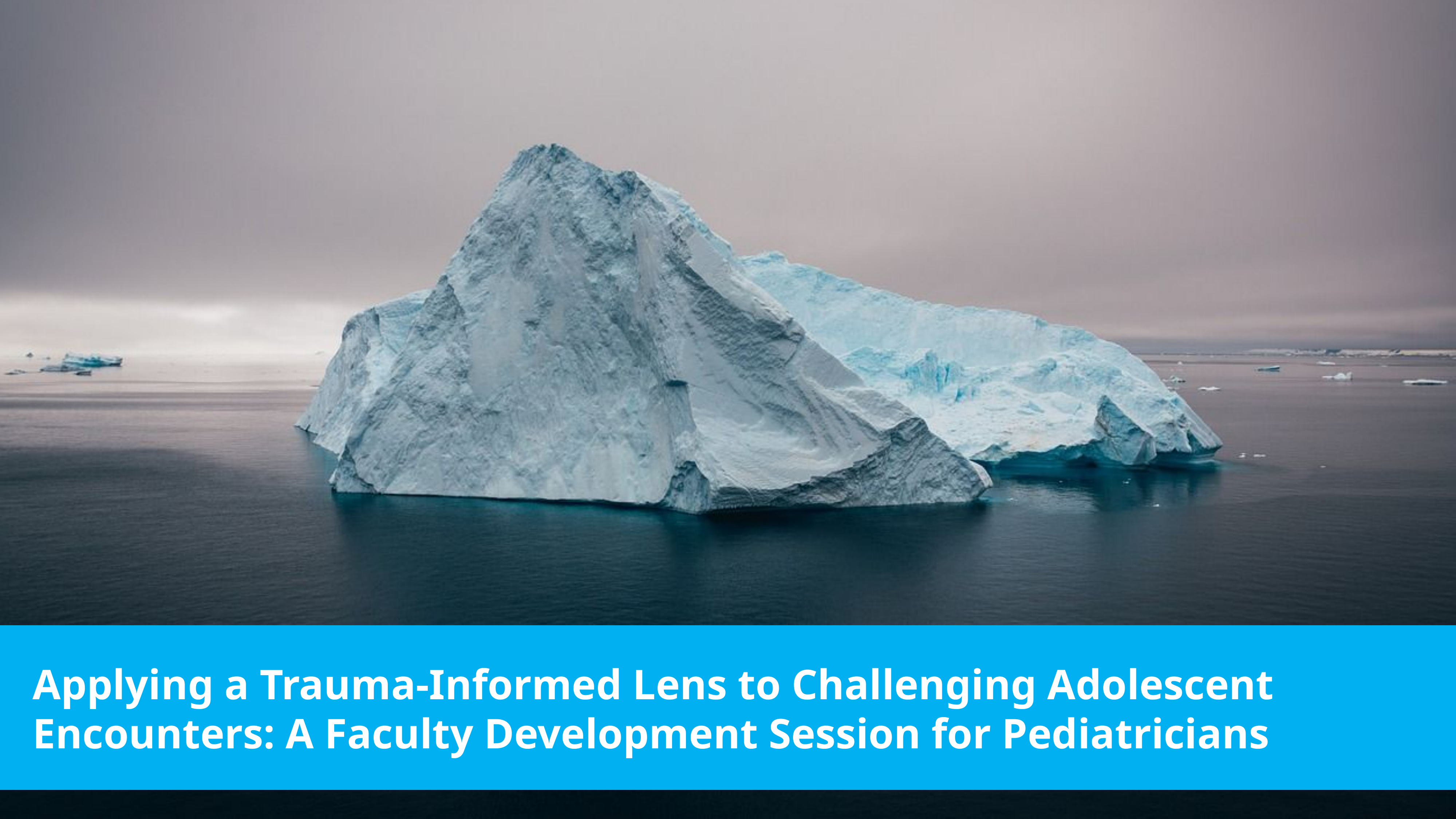

Applying a Trauma-Informed Lens to Challenging Adolescent Encounters: A Faculty Development Session for Pediatricians

## Slide 2
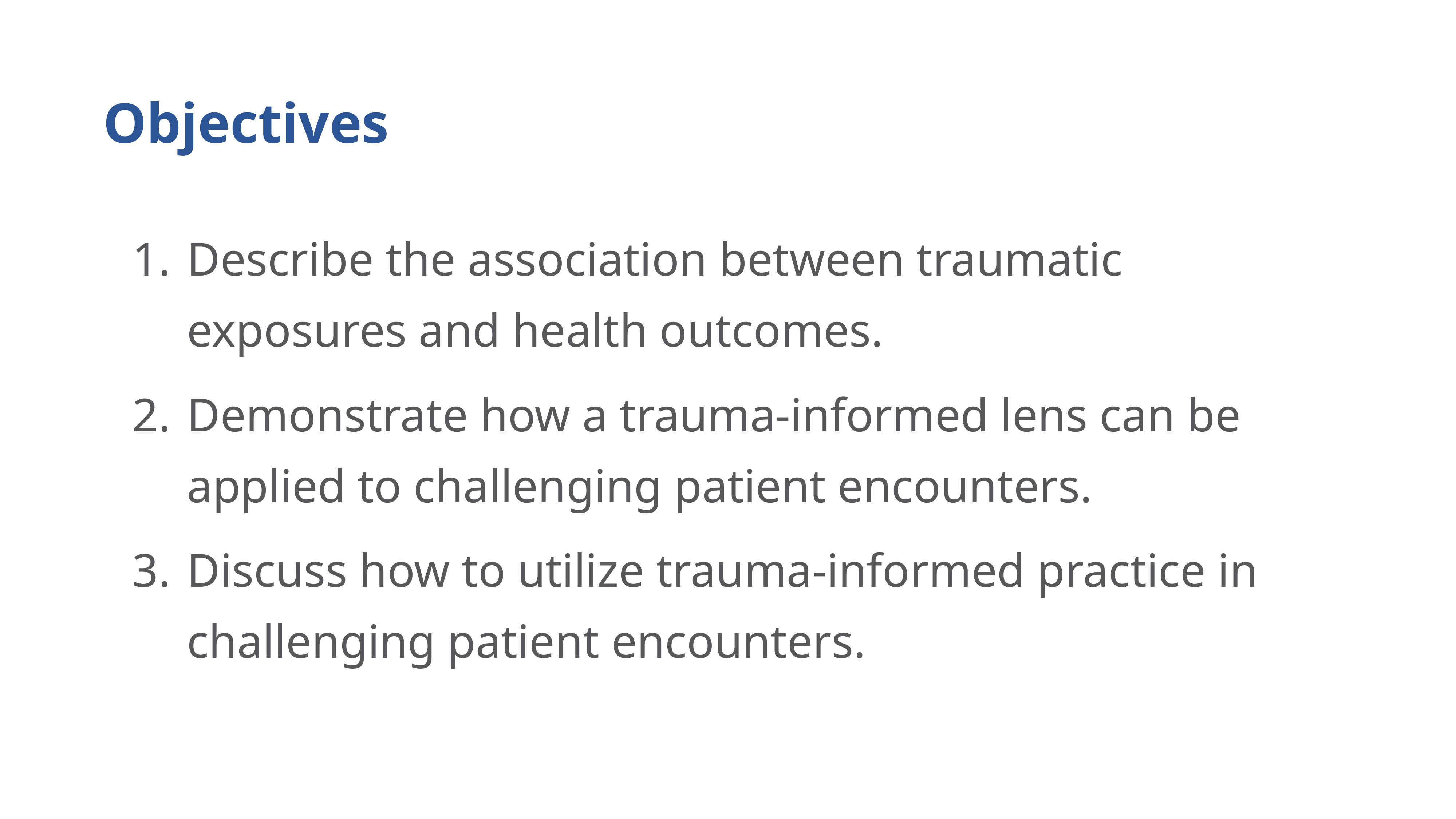

Objectives
Describe the association between traumatic exposures and health outcomes.
Demonstrate how a trauma-informed lens can be applied to challenging patient encounters.
Discuss how to utilize trauma-informed practice in challenging patient encounters.

## Slide 3
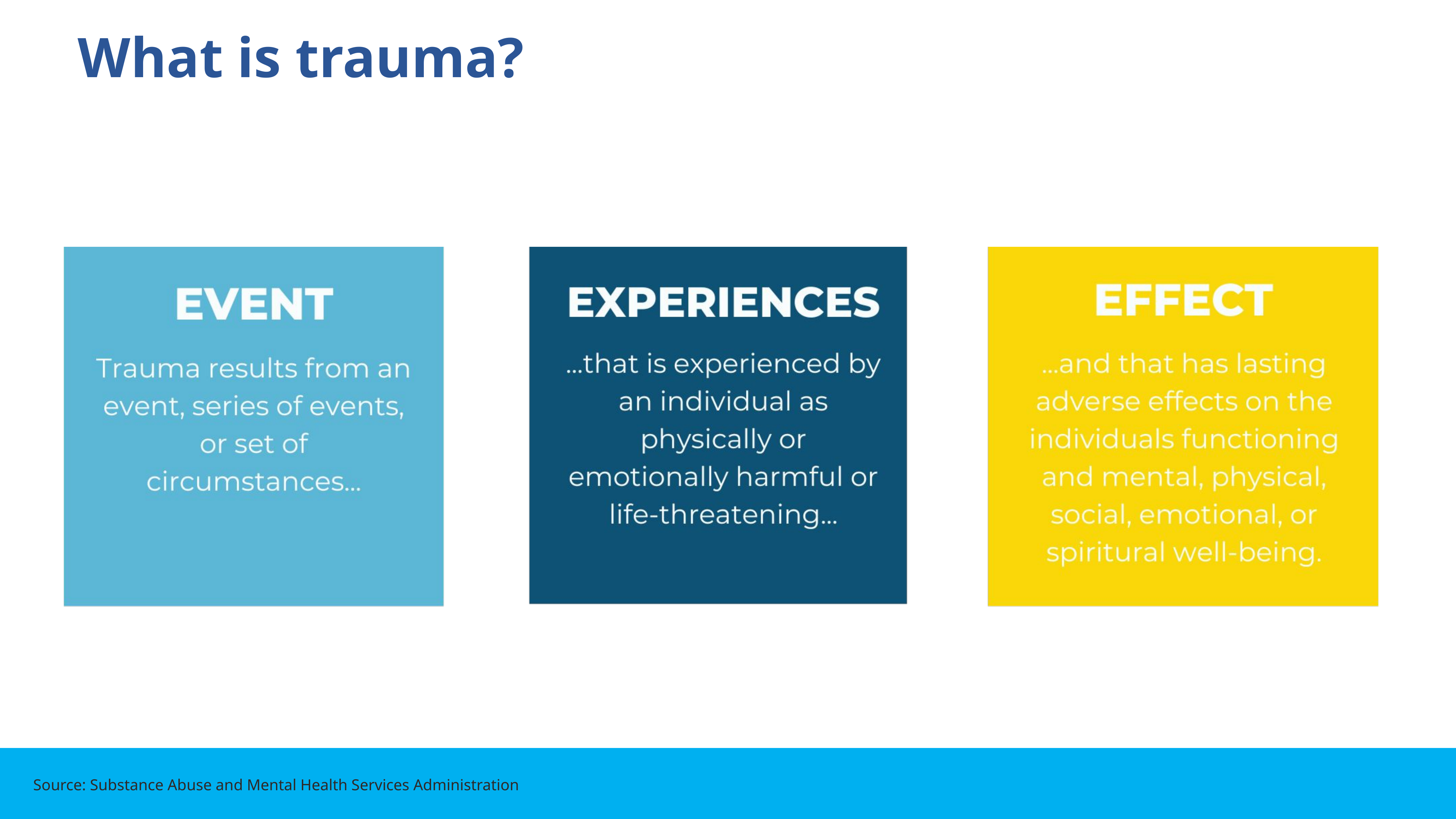

# What is trauma?
Source: Substance Abuse and Mental Health Services Administration

## Slide 4
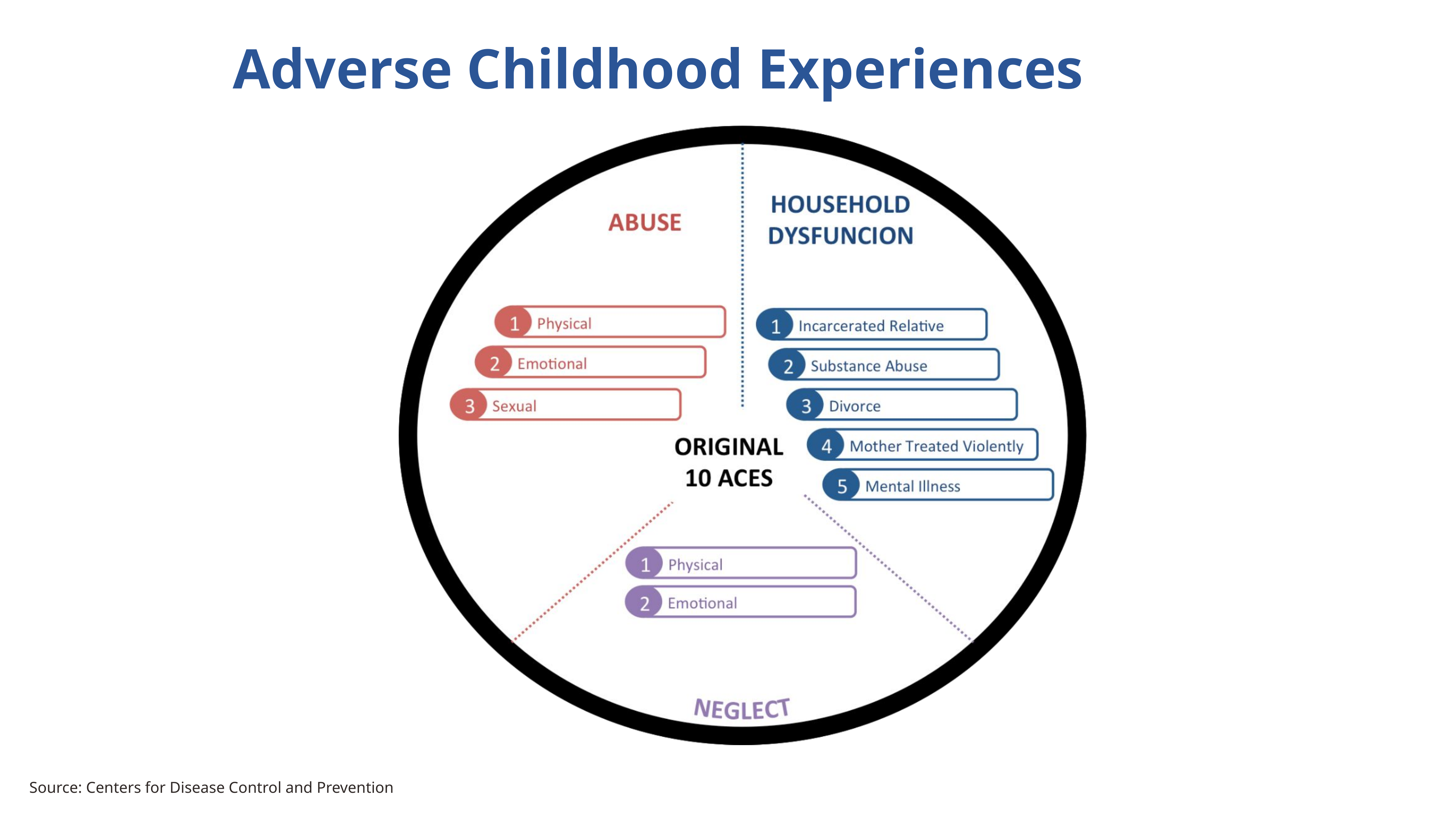

Adverse Childhood Experiences
Source: Centers for Disease Control and Prevention

## Slide 5
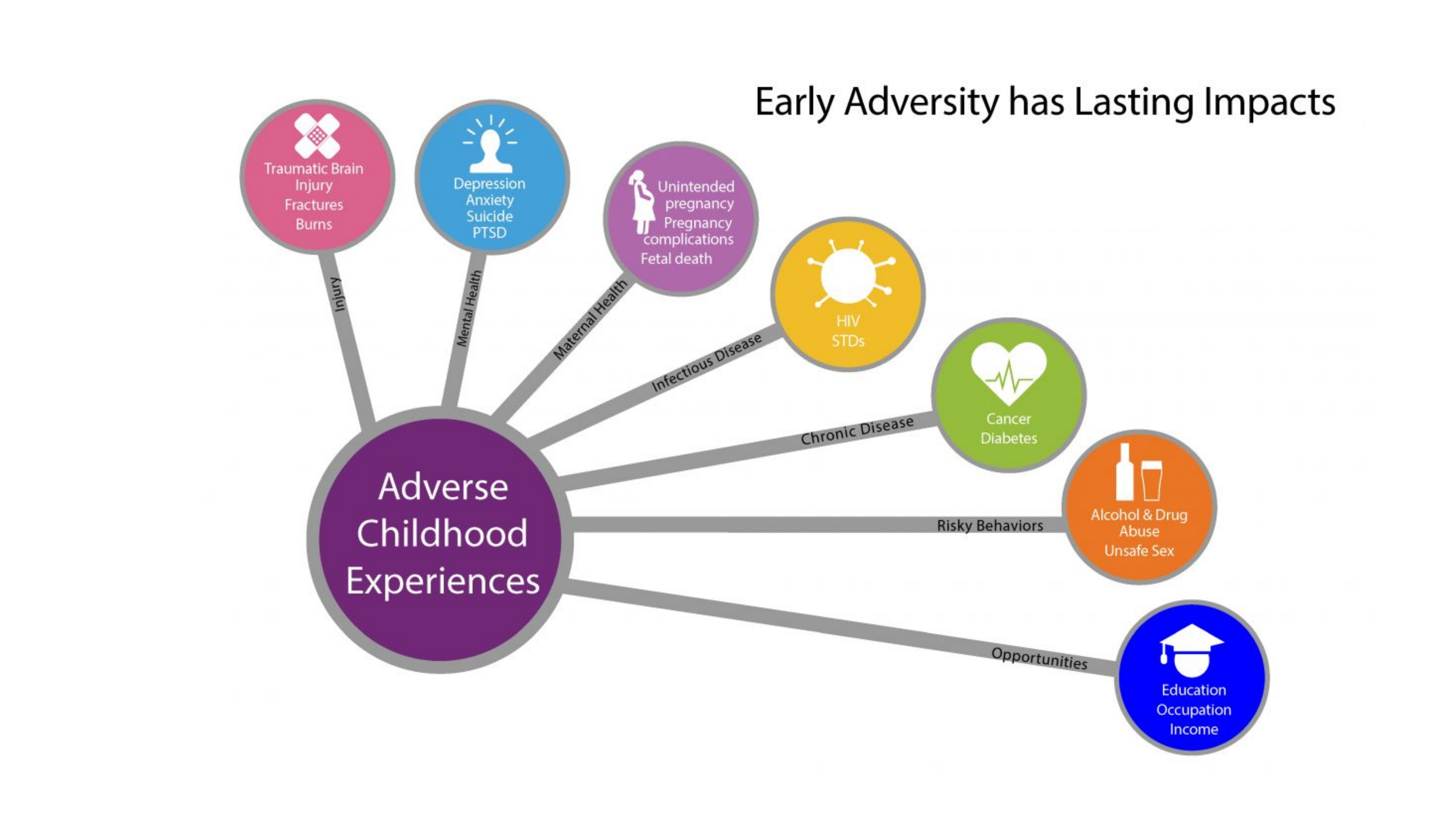

## Slide 6
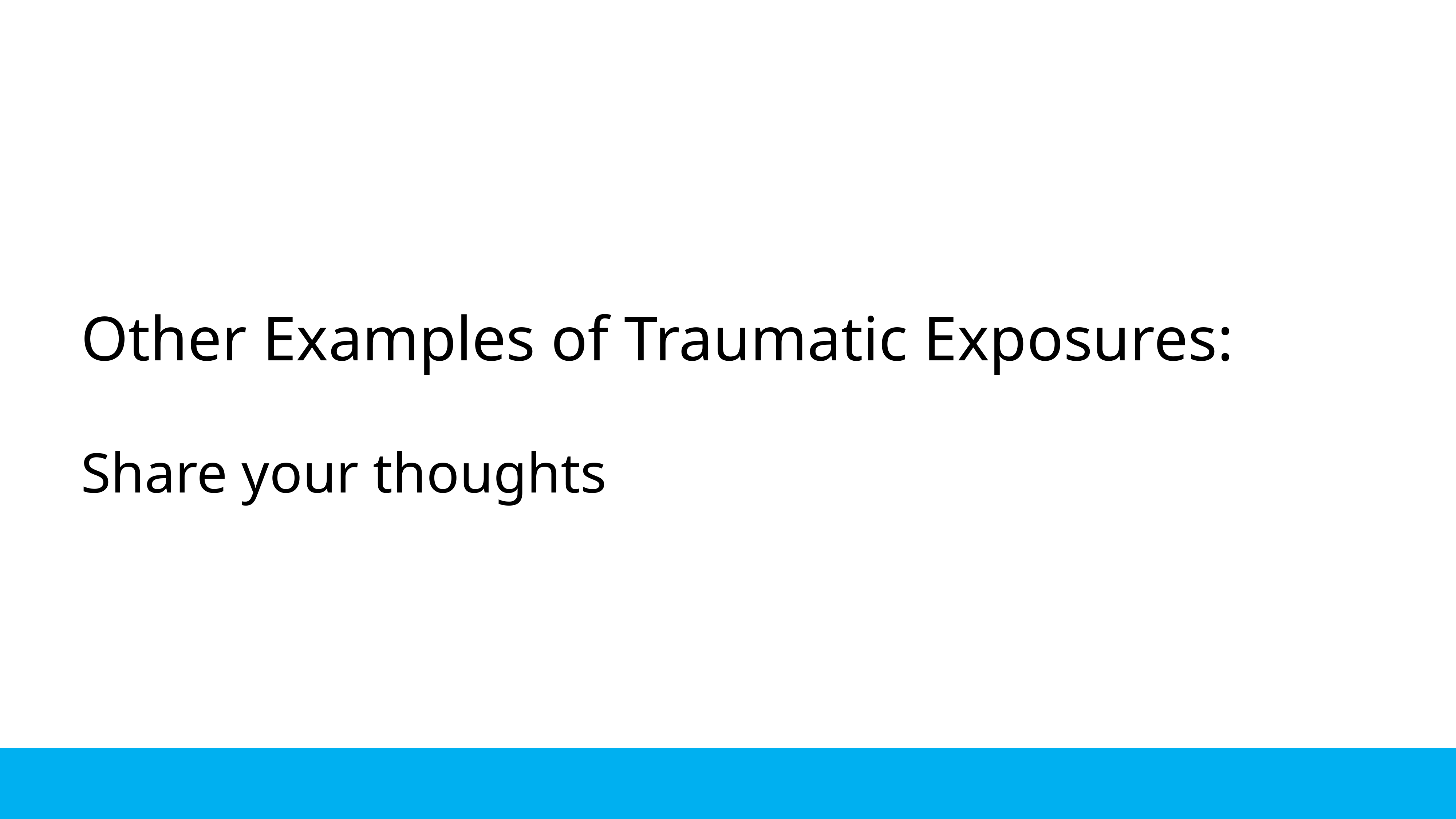

Other Examples of Traumatic Exposures:
Share your thoughts

## Slide 7
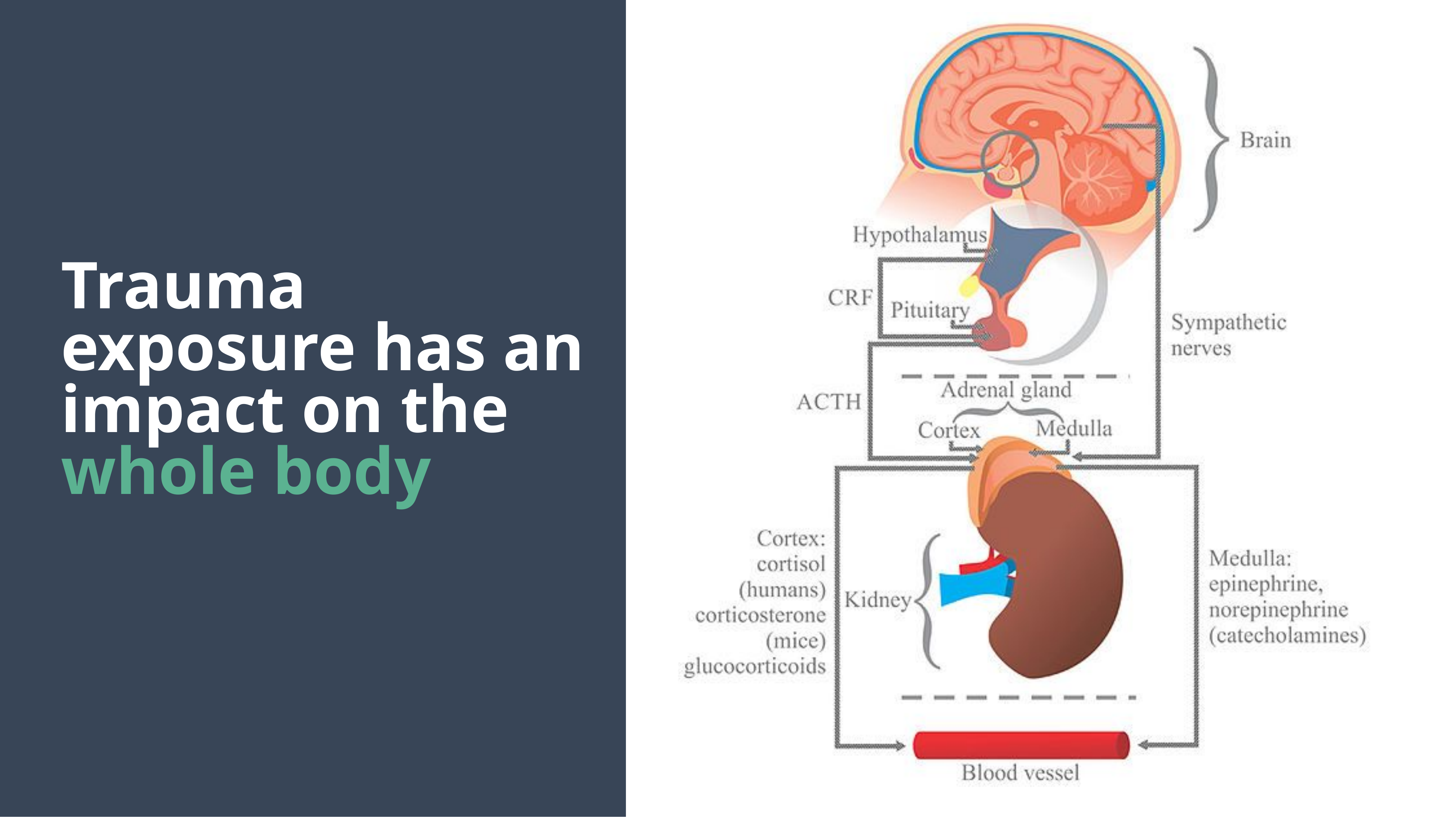

Trauma exposure has an impact on the whole body

## Slide 8
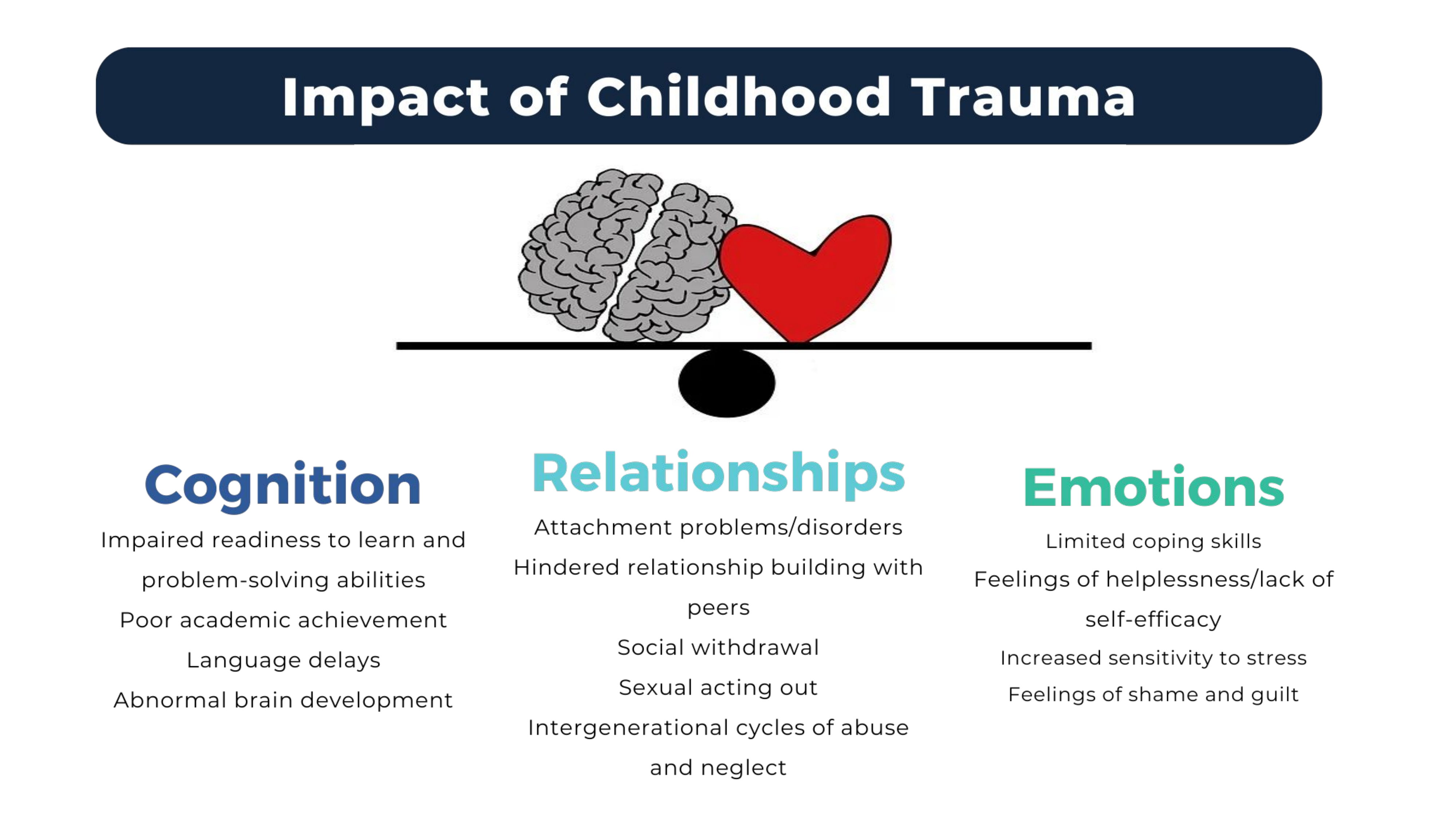

## Slide 9
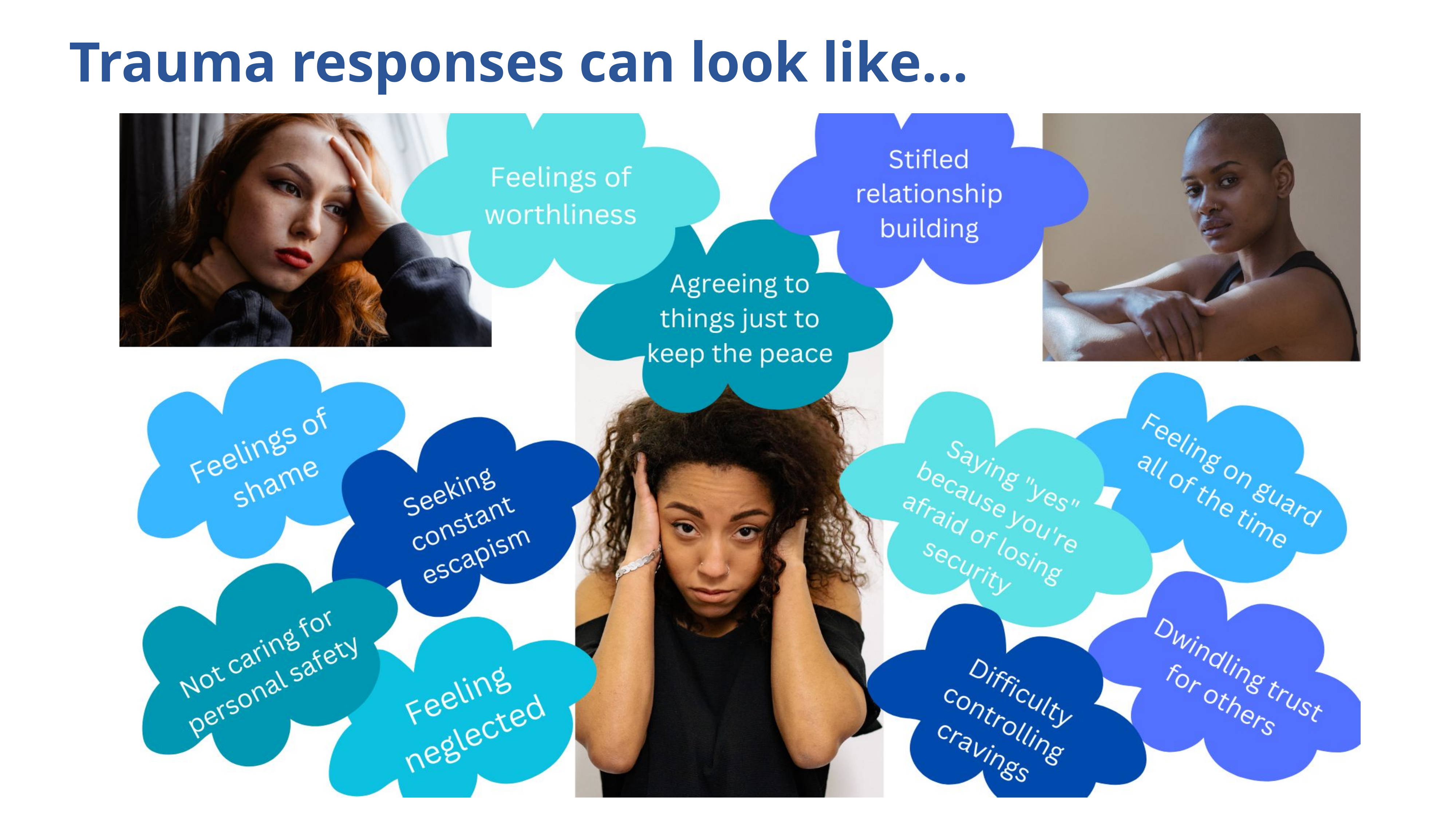

Trauma responses can look like…

## Slide 10
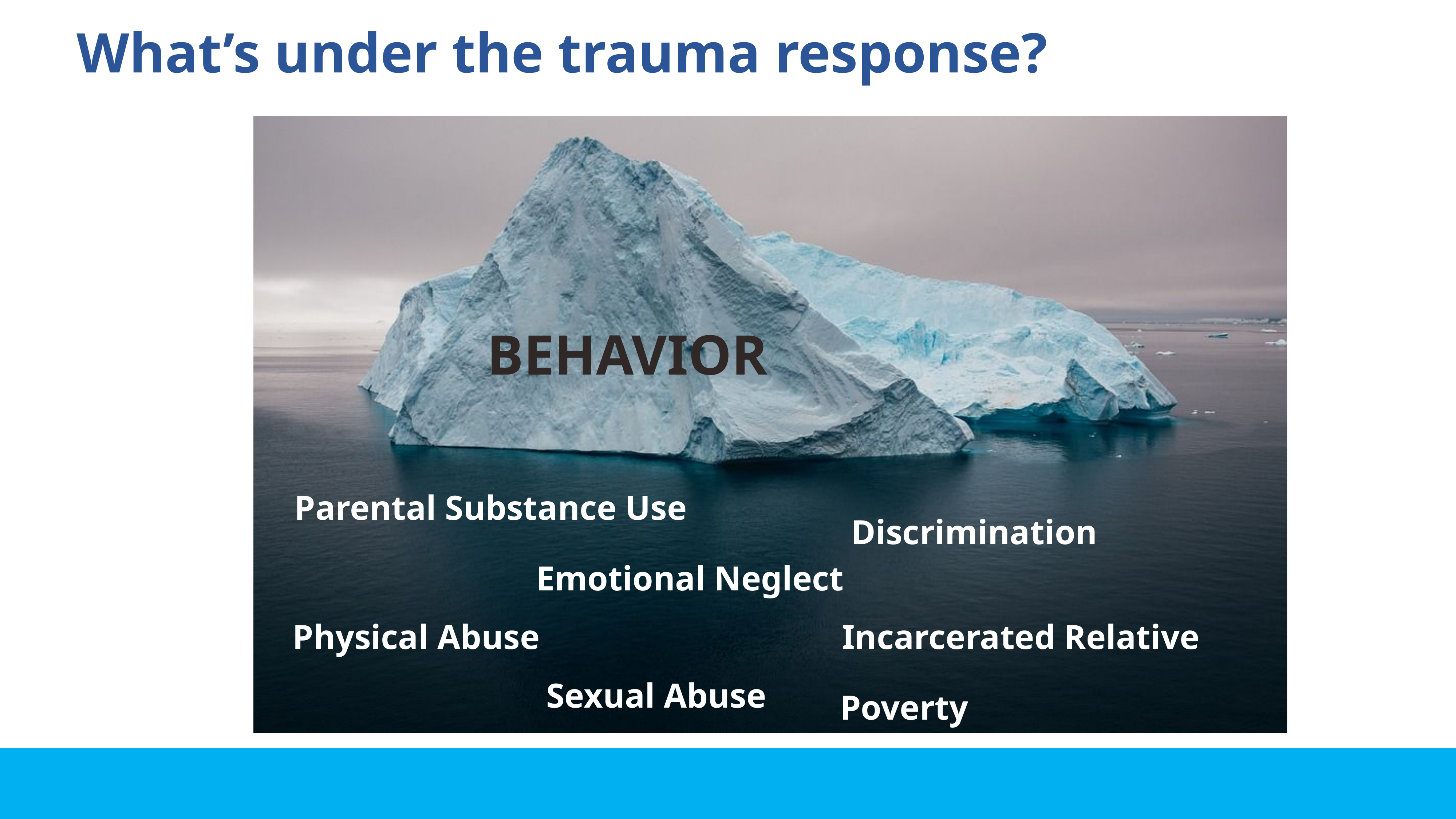

# What’s under the trauma response?
BEHAVIOR
Parental Substance Use
Discrimination
Emotional Neglect
Incarcerated Relative
Physical Abuse
Sexual Abuse
Poverty

## Slide 11
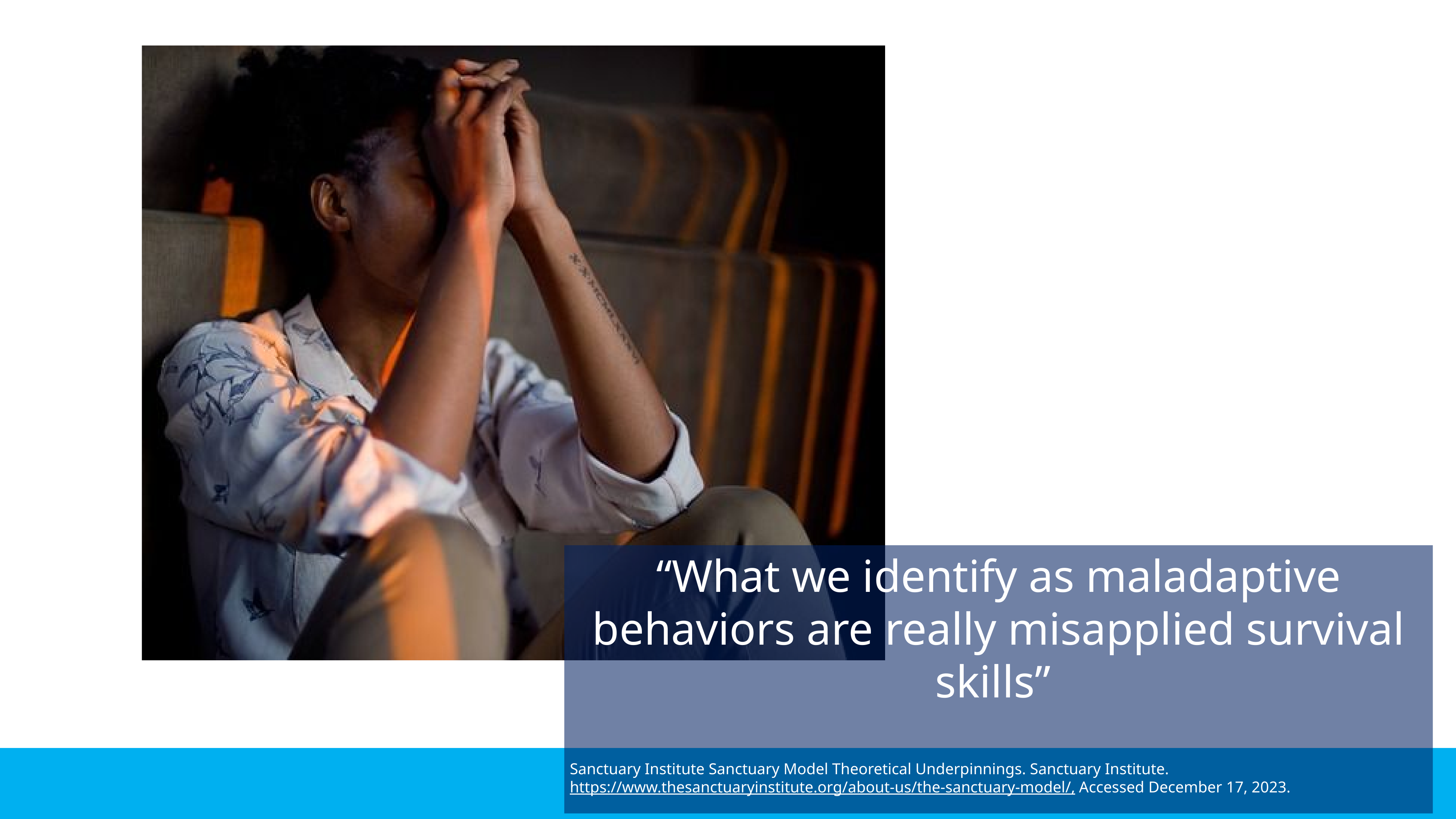

“What we identify as maladaptive behaviors are really misapplied survival skills”
Sanctuary Institute Sanctuary Model Theoretical Underpinnings. Sanctuary Institute. https://www.thesanctuaryinstitute.org/about-us/the-sanctuary-model/, Accessed December 17, 2023.

## Slide 12
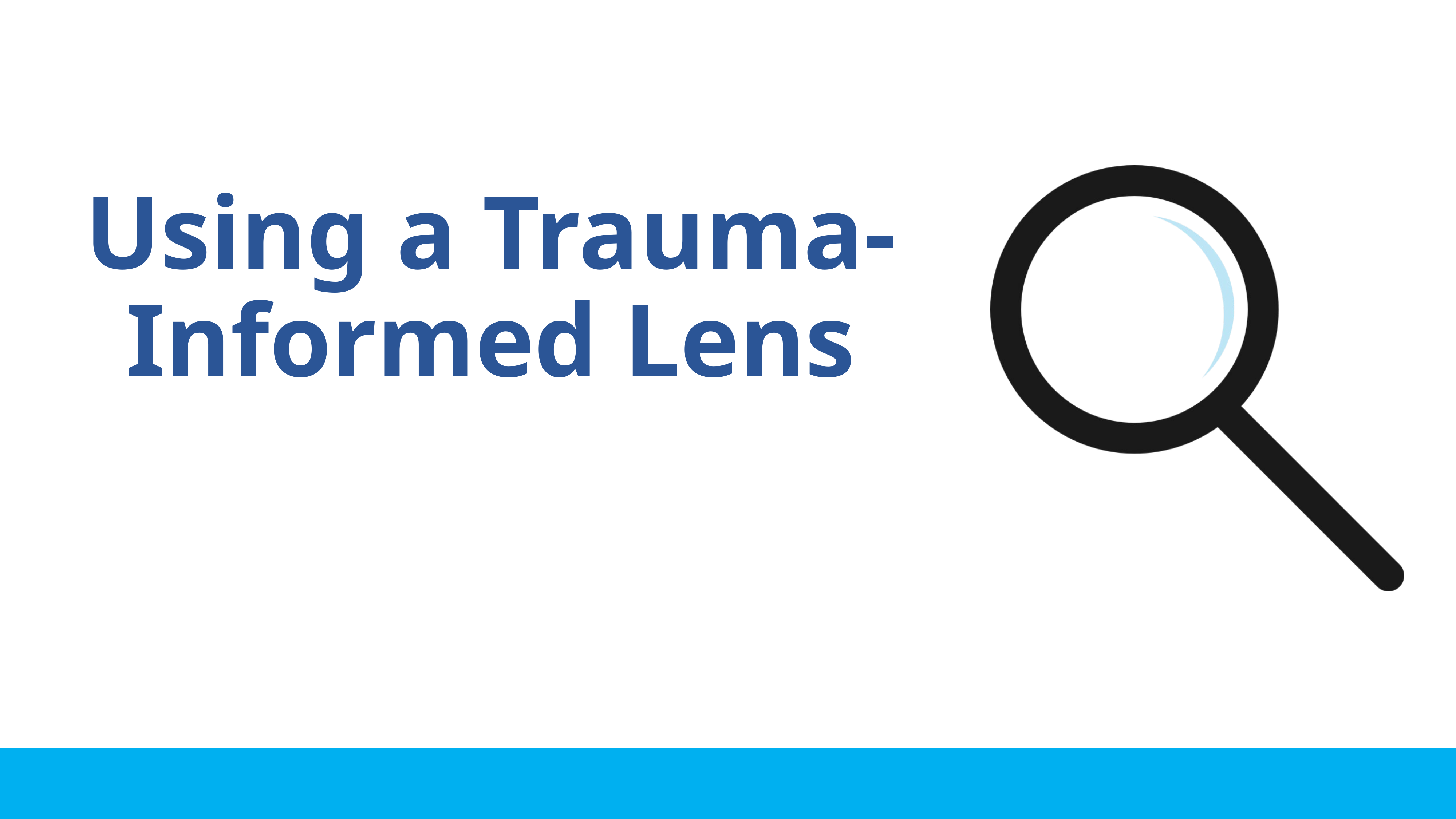

Using a Trauma-Informed Lens

## Slide 13
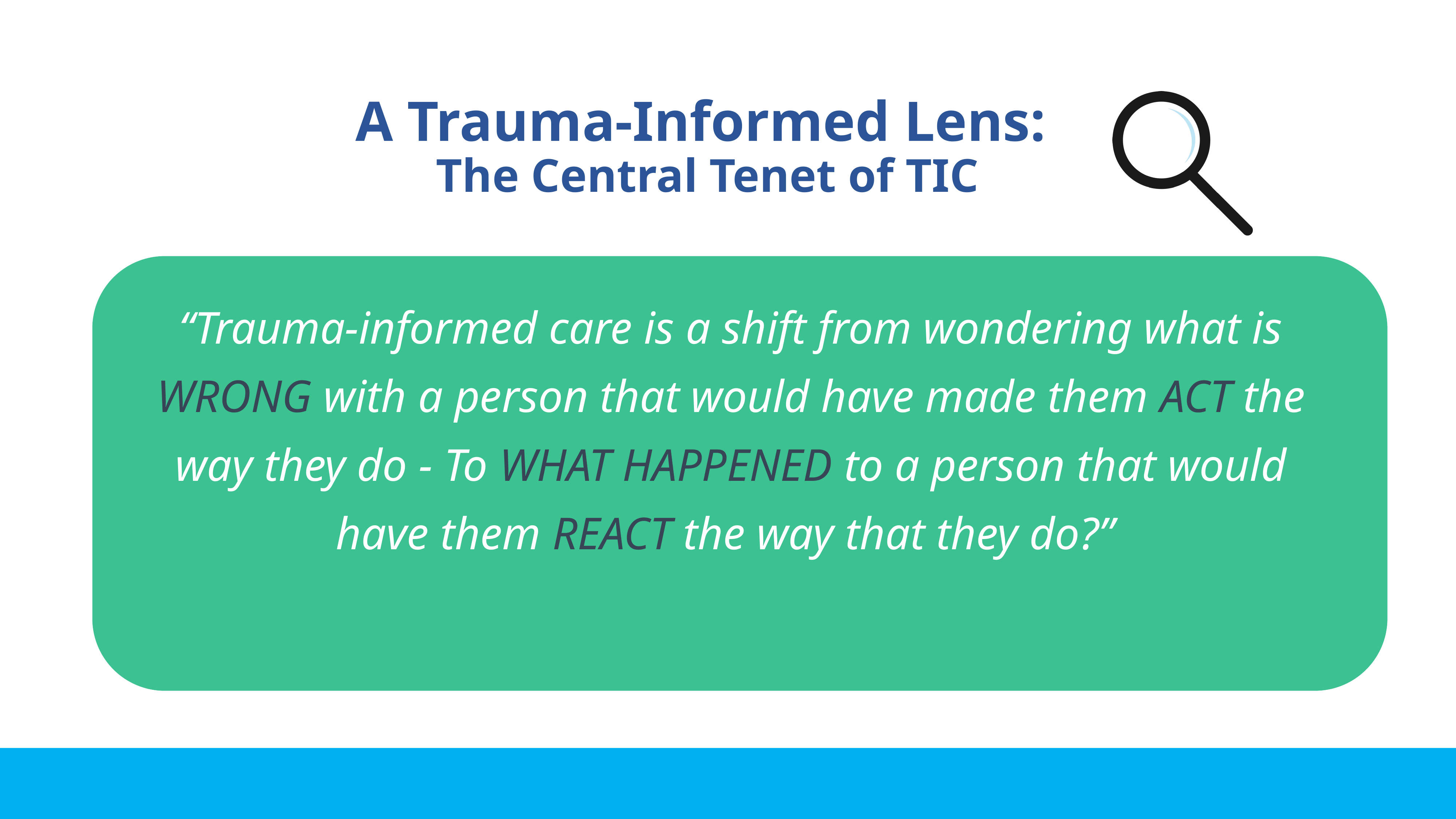

A Trauma-Informed Lens:
The Central Tenet of TIC
“Trauma-informed care is a shift from wondering what is WRONG with a person that would have made them ACT the way they do - To WHAT HAPPENED to a person that would have them REACT the way that they do?”

## Slide 14
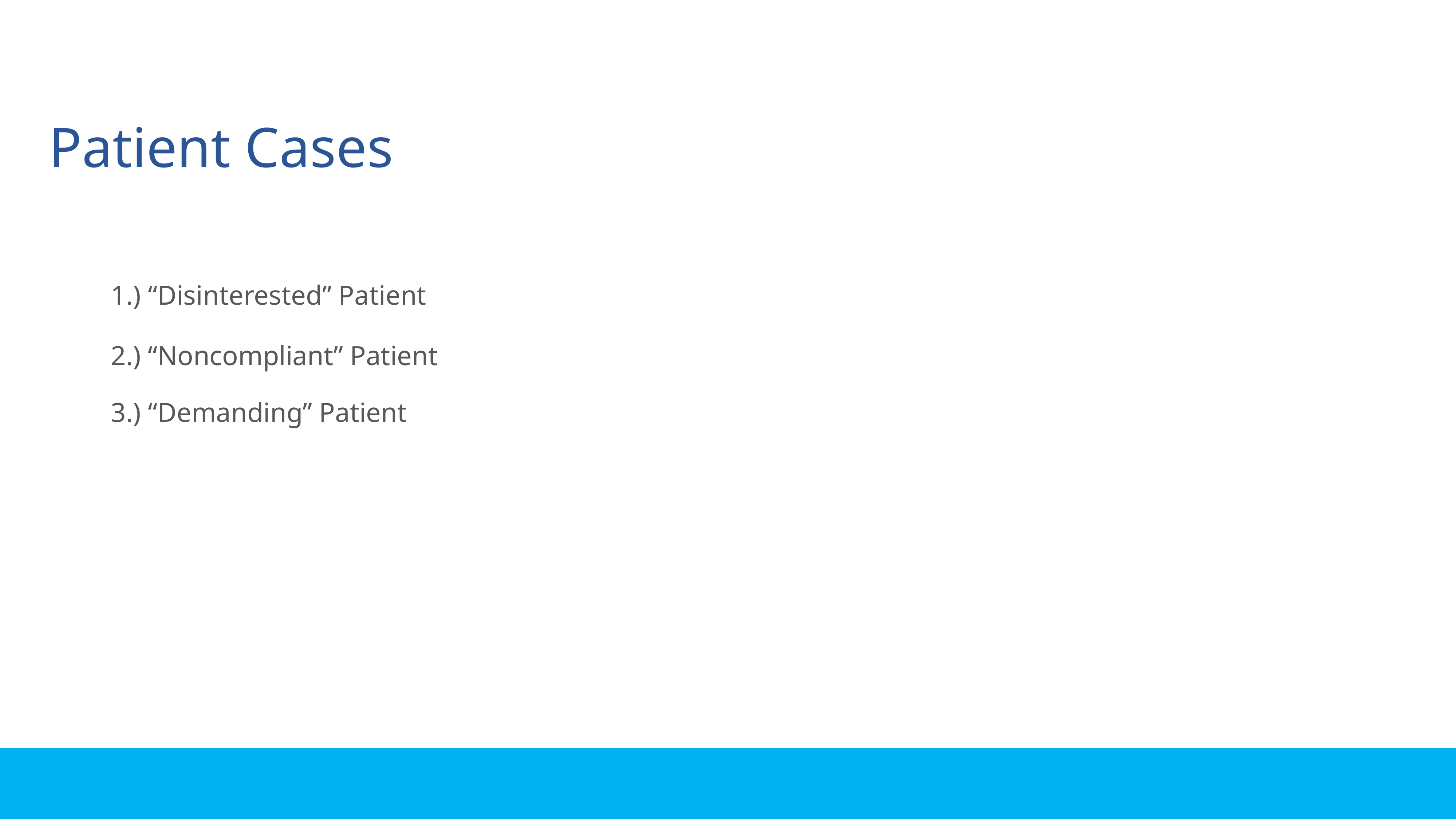

# Patient Cases
1.) “Disinterested” Patient
2.) “Noncompliant” Patient
3.) “Demanding” Patient

## Slide 15
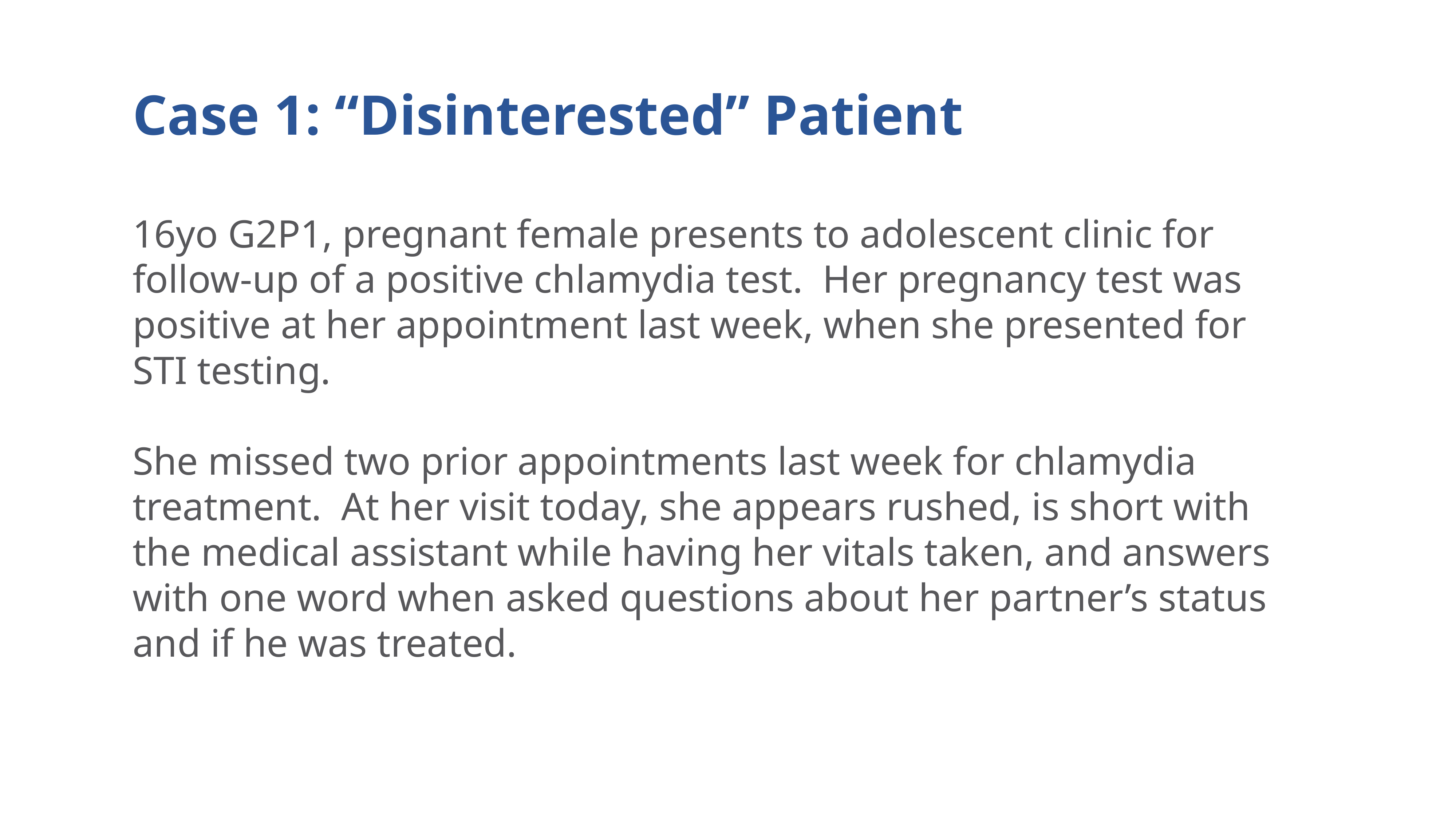

Case 1: “Disinterested” Patient
16yo G2P1, pregnant female presents to adolescent clinic for follow-up of a positive chlamydia test. Her pregnancy test was positive at her appointment last week, when she presented for STI testing.
She missed two prior appointments last week for chlamydia treatment. At her visit today, she appears rushed, is short with the medical assistant while having her vitals taken, and answers with one word when asked questions about her partner’s status and if he was treated.

## Slide 16
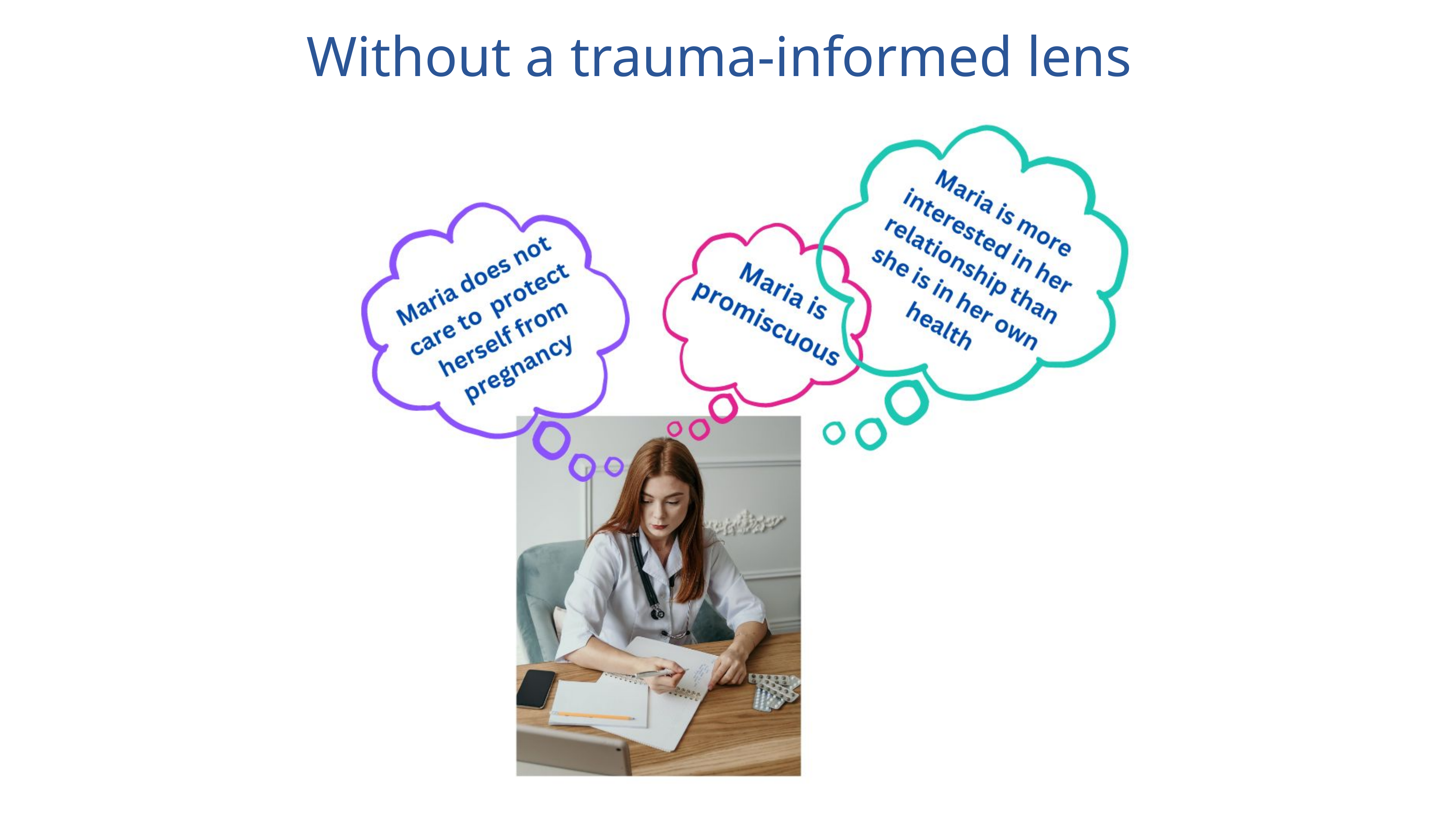

# Without a trauma-informed lens

## Slide 17
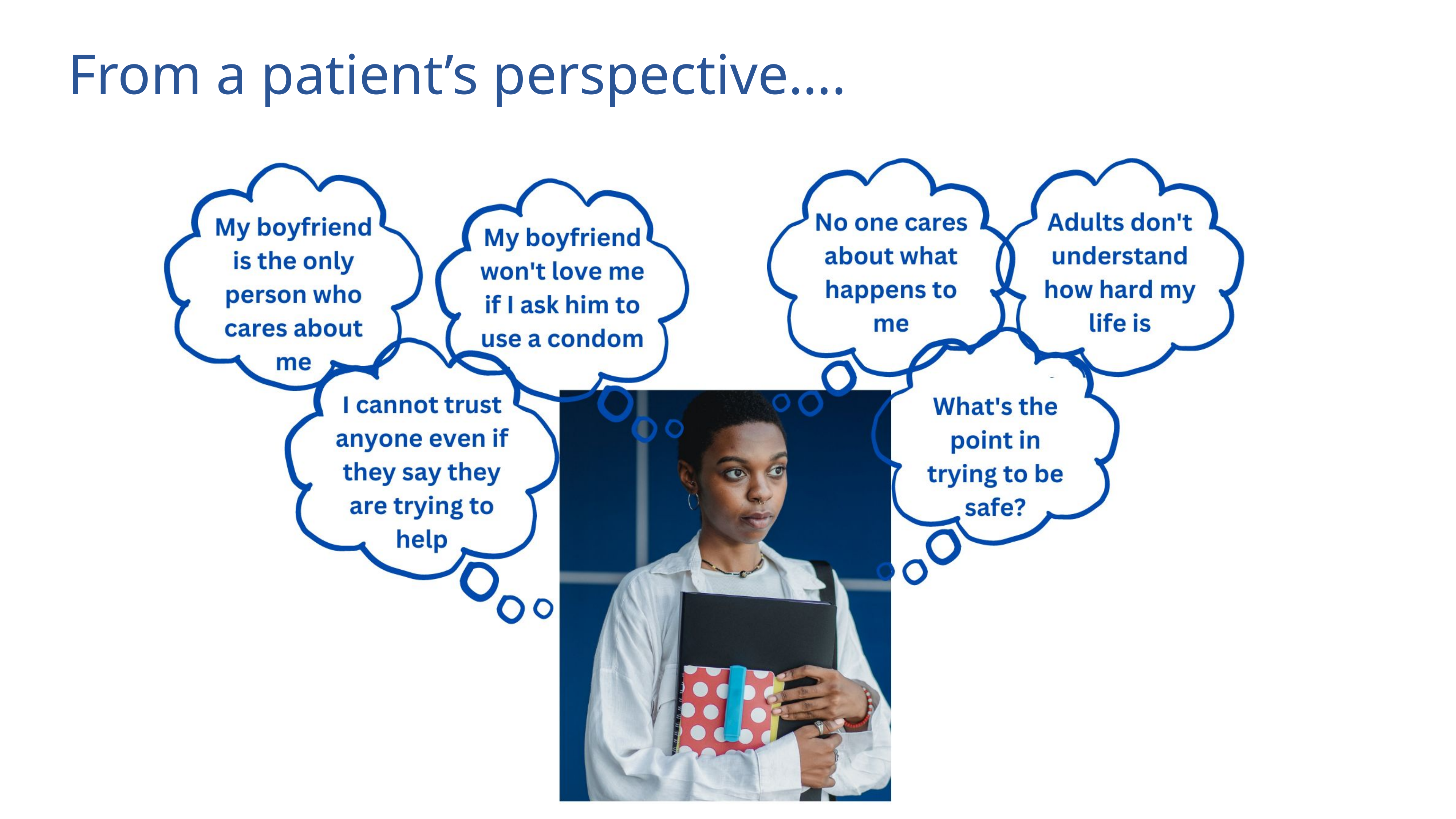

# From a patient’s perspective….

## Slide 18
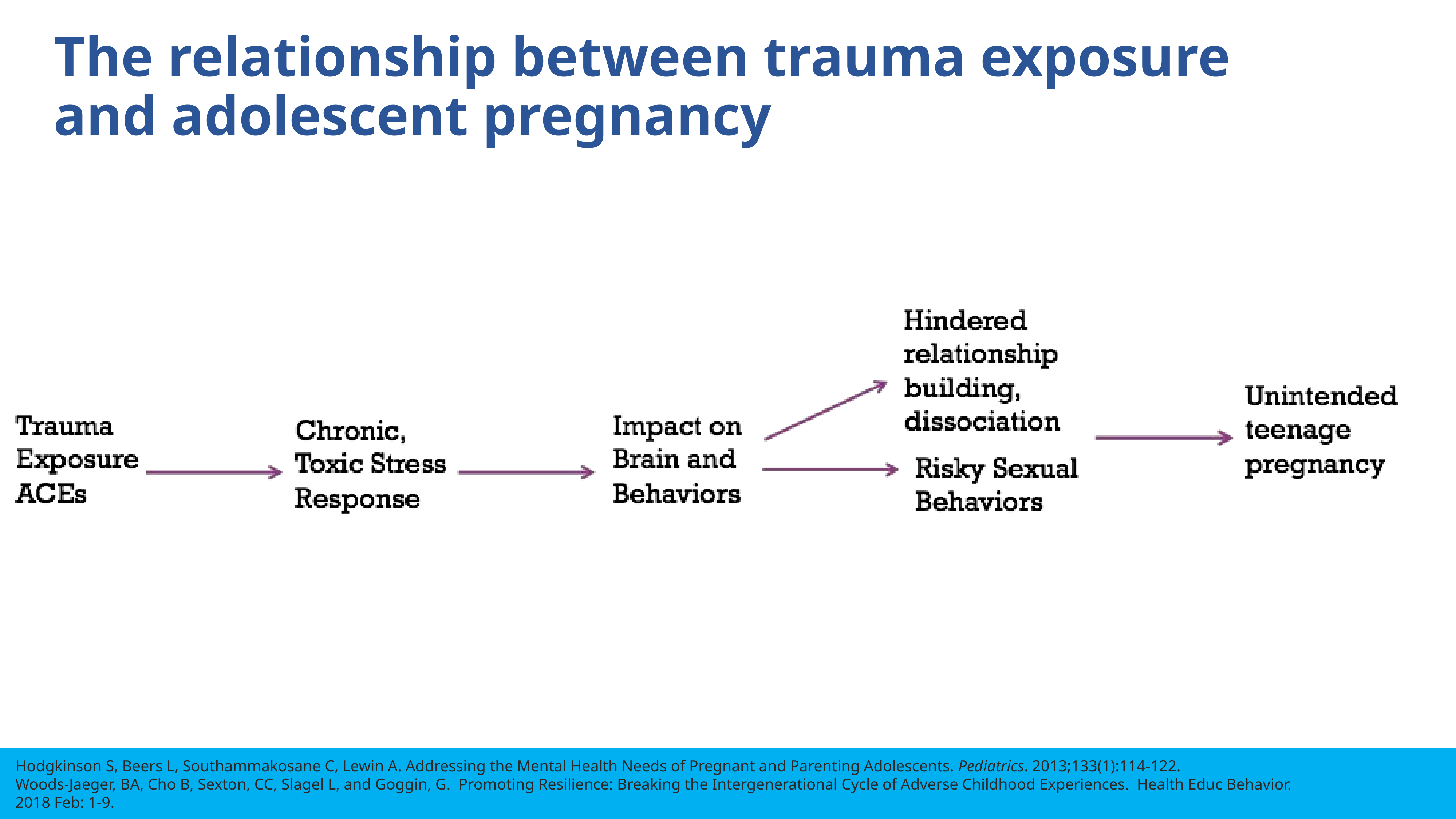

# The relationship between trauma exposure and adolescent pregnancy
Hodgkinson S, Beers L, Southammakosane C, Lewin A. Addressing the Mental Health Needs of Pregnant and Parenting Adolescents. Pediatrics. 2013;133(1):114-122.
Woods-Jaeger, BA, Cho B, Sexton, CC, Slagel L, and Goggin, G. Promoting Resilience: Breaking the Intergenerational Cycle of Adverse Childhood Experiences. Health Educ Behavior. 2018 Feb: 1-9.

## Slide 19
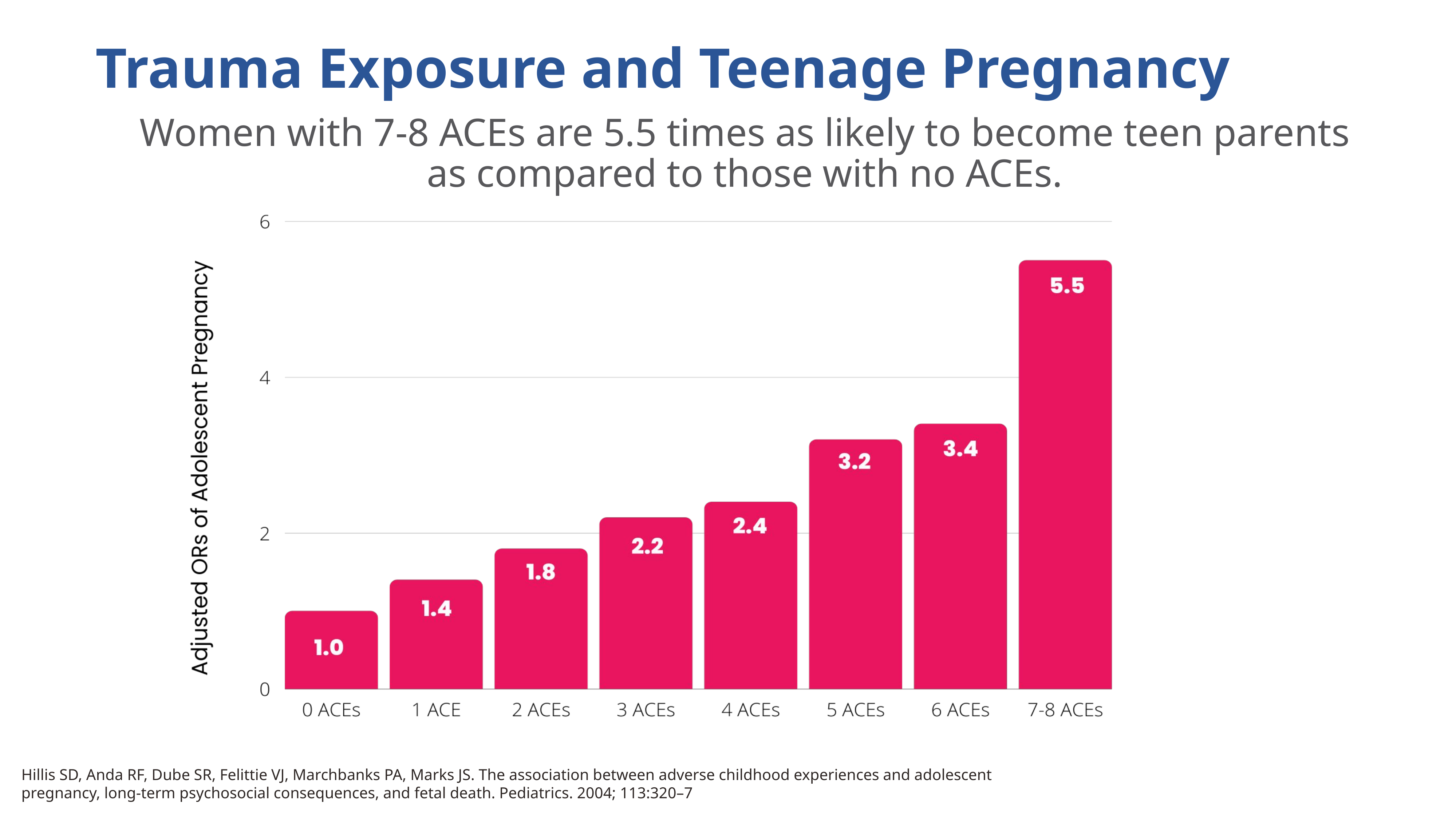

Trauma Exposure and Teenage Pregnancy
Women with 7-8 ACEs are 5.5 times as likely to become teen parents as compared to those with no ACEs.
Hillis SD, Anda RF, Dube SR, Felittie VJ, Marchbanks PA, Marks JS. The association between adverse childhood experiences and adolescent pregnancy, long-term psychosocial consequences, and fetal death. Pediatrics. 2004; 113:320–7

## Slide 20
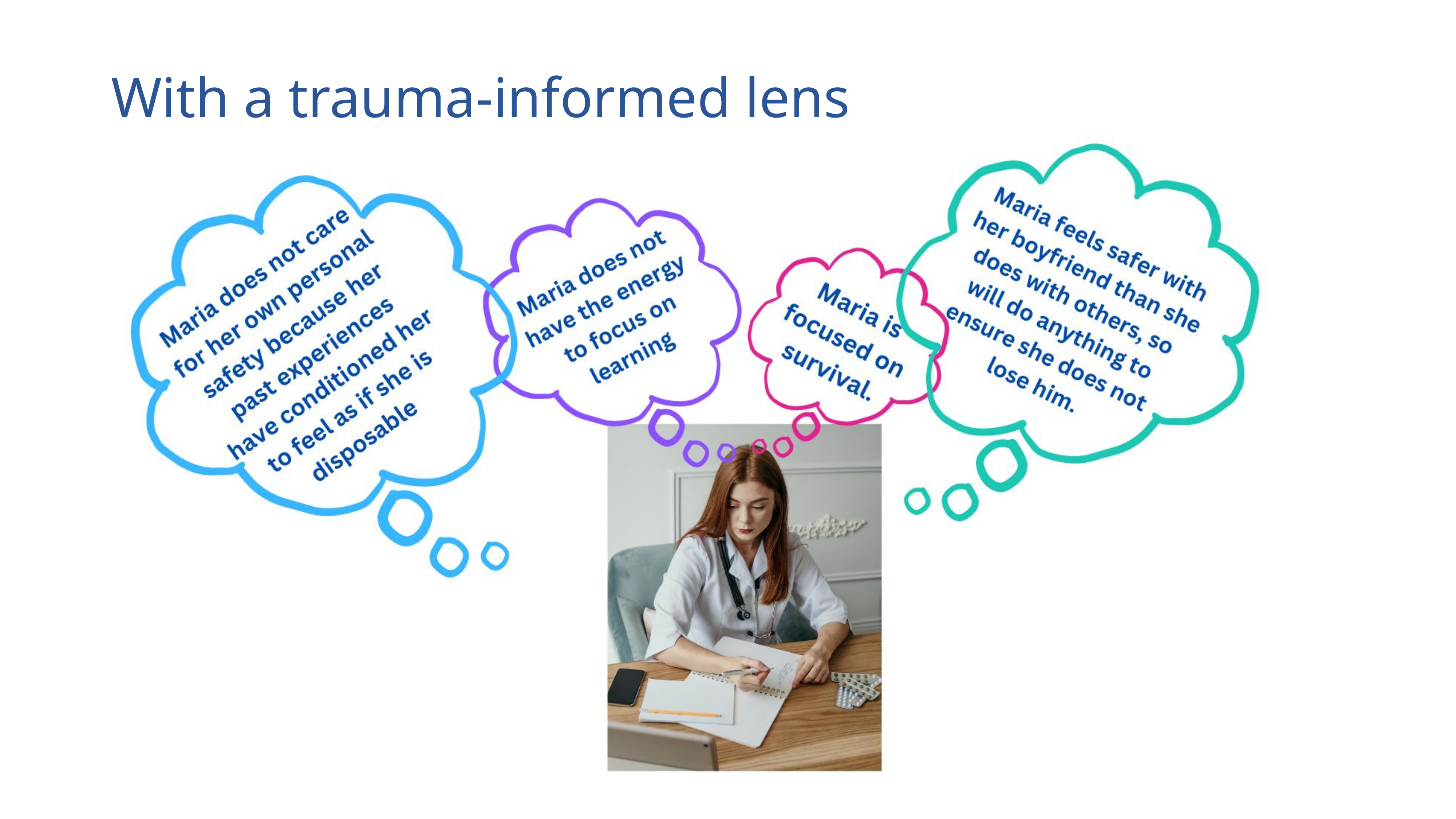

# With a trauma-informed lens

## Slide 21
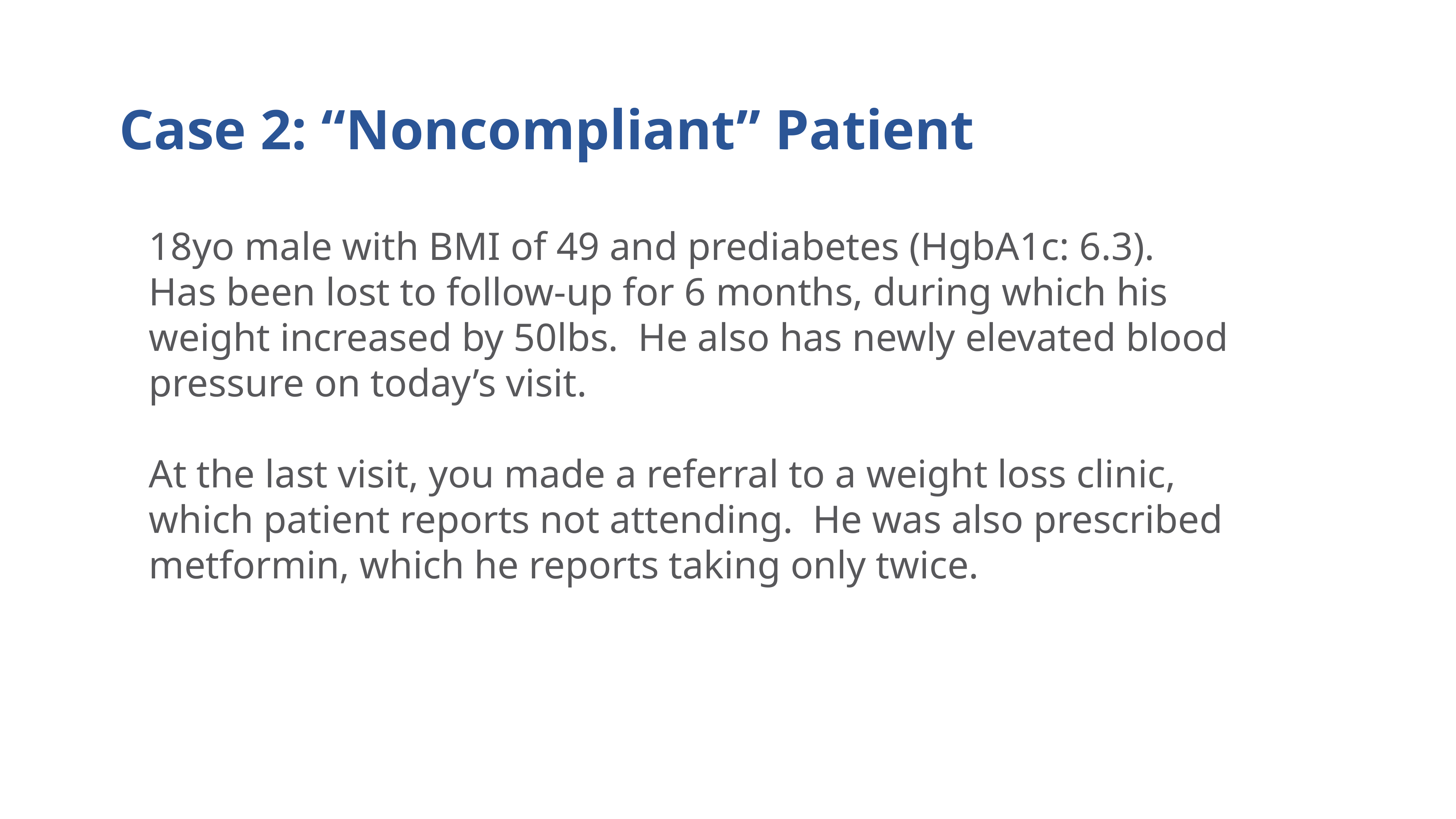

Case 2: “Noncompliant” Patient
18yo male with BMI of 49 and prediabetes (HgbA1c: 6.3). Has been lost to follow-up for 6 months, during which his weight increased by 50lbs. He also has newly elevated blood pressure on today’s visit.
At the last visit, you made a referral to a weight loss clinic, which patient reports not attending. He was also prescribed metformin, which he reports taking only twice.

## Slide 22
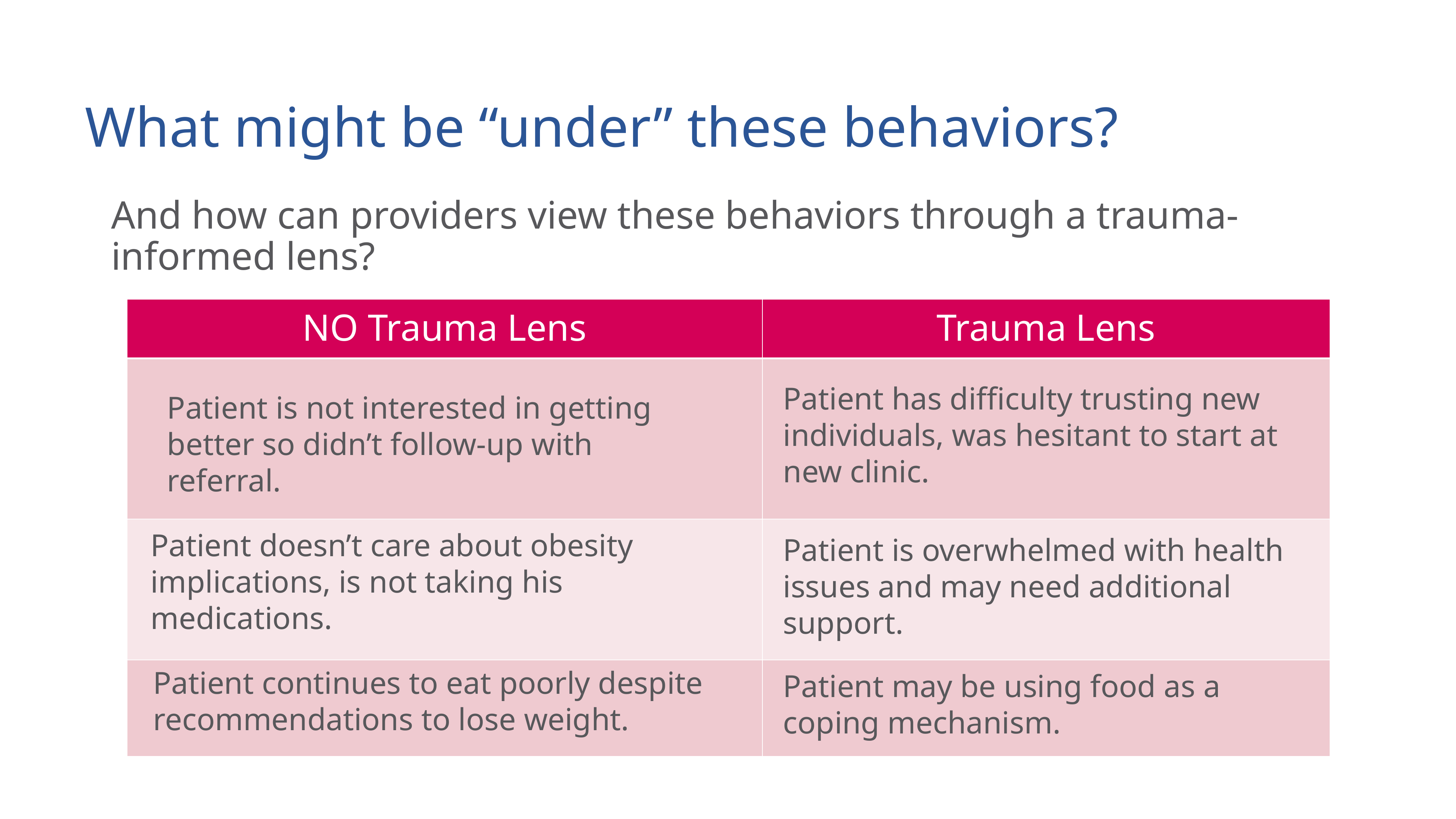

# What might be “under” these behaviors?
And how can providers view these behaviors through a trauma-informed lens?
| NO Trauma Lens | Trauma Lens |
| --- | --- |
| | |
| | |
| | |
Patient has difficulty trusting new individuals, was hesitant to start at new clinic.
Patient is not interested in getting better so didn’t follow-up with referral.
Patient doesn’t care about obesity implications, is not taking his medications.
Patient is overwhelmed with health issues and may need additional support.
Patient continues to eat poorly despite recommendations to lose weight.
Patient may be using food as a coping mechanism.

## Slide 23
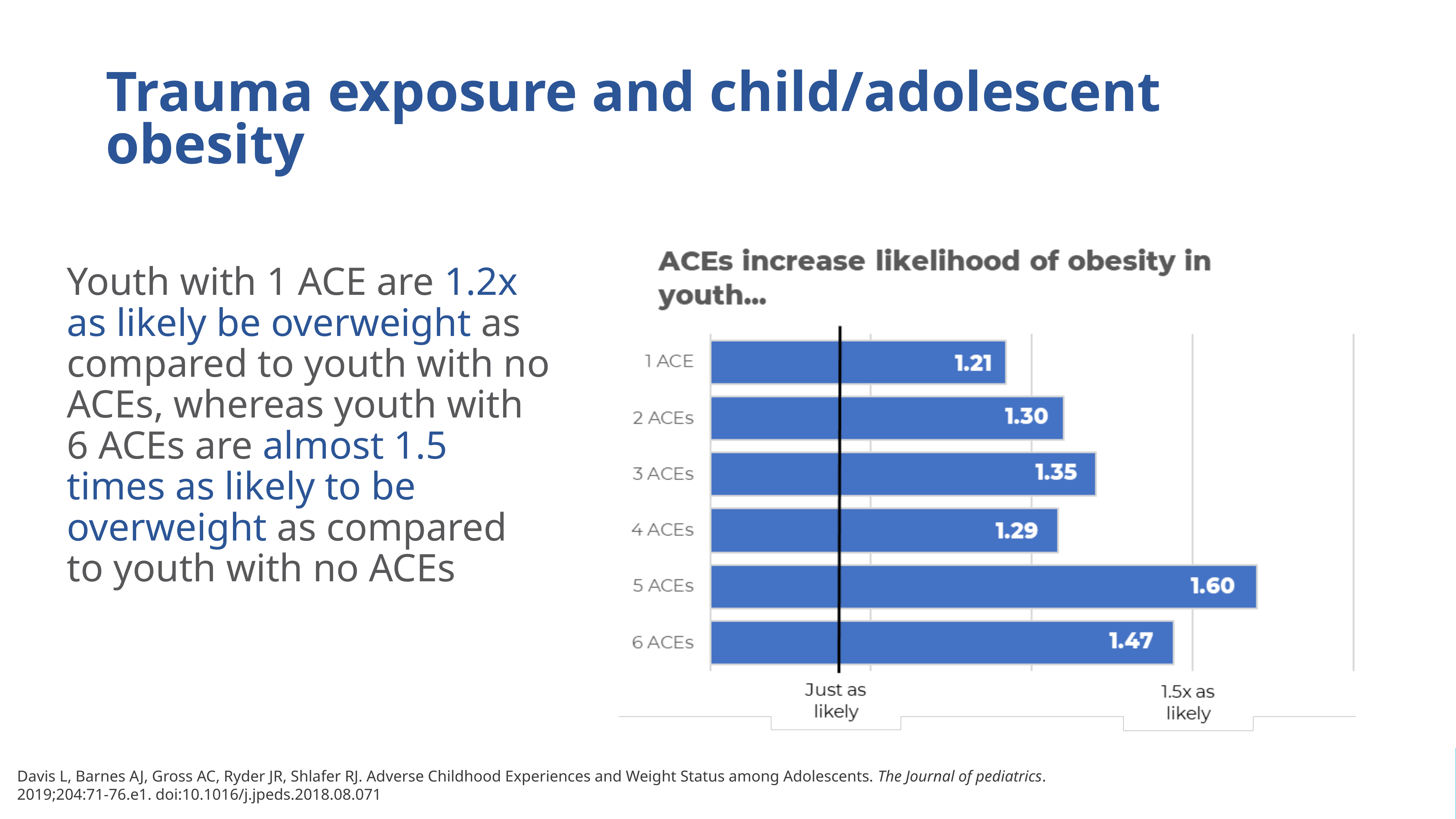

# Trauma exposure and child/adolescent obesity
Youth with 1 ACE are 1.2x as likely be overweight as compared to youth with no ACEs, whereas youth with 6 ACEs are almost 1.5 times as likely to be overweight as compared to youth with no ACEs
Davis L, Barnes AJ, Gross AC, Ryder JR, Shlafer RJ. Adverse Childhood Experiences and Weight Status among Adolescents. The Journal of pediatrics. 2019;204:71-76.e1. doi:10.1016/j.jpeds.2018.08.071

## Slide 24
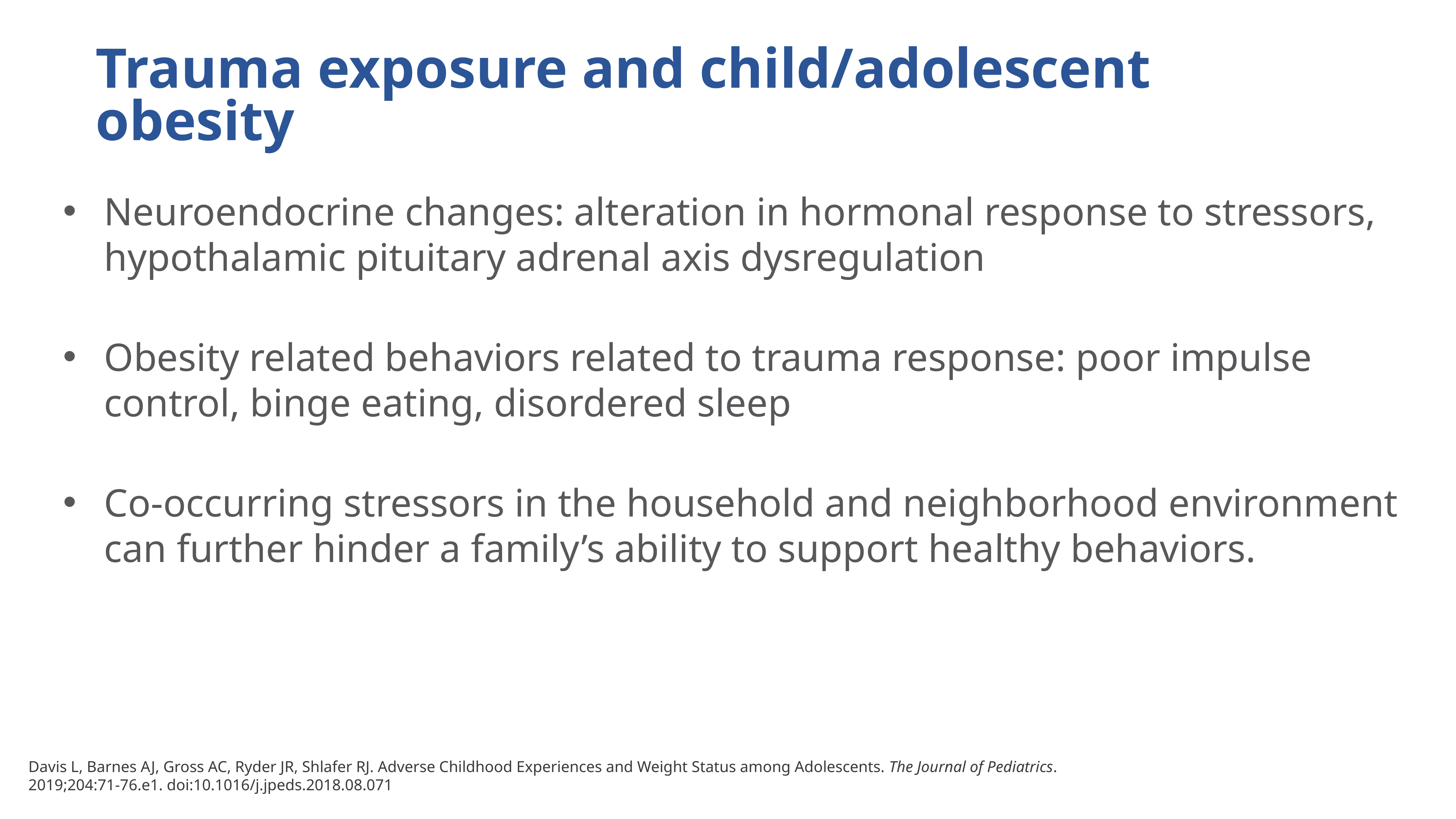

Trauma exposure and child/adolescent obesity
Neuroendocrine changes: alteration in hormonal response to stressors, hypothalamic pituitary adrenal axis dysregulation
Obesity related behaviors related to trauma response: poor impulse control, binge eating, disordered sleep
Co-occurring stressors in the household and neighborhood environment can further hinder a family’s ability to support healthy behaviors.
Davis L, Barnes AJ, Gross AC, Ryder JR, Shlafer RJ. Adverse Childhood Experiences and Weight Status among Adolescents. The Journal of Pediatrics. 2019;204:71-76.e1. doi:10.1016/j.jpeds.2018.08.071

## Slide 25
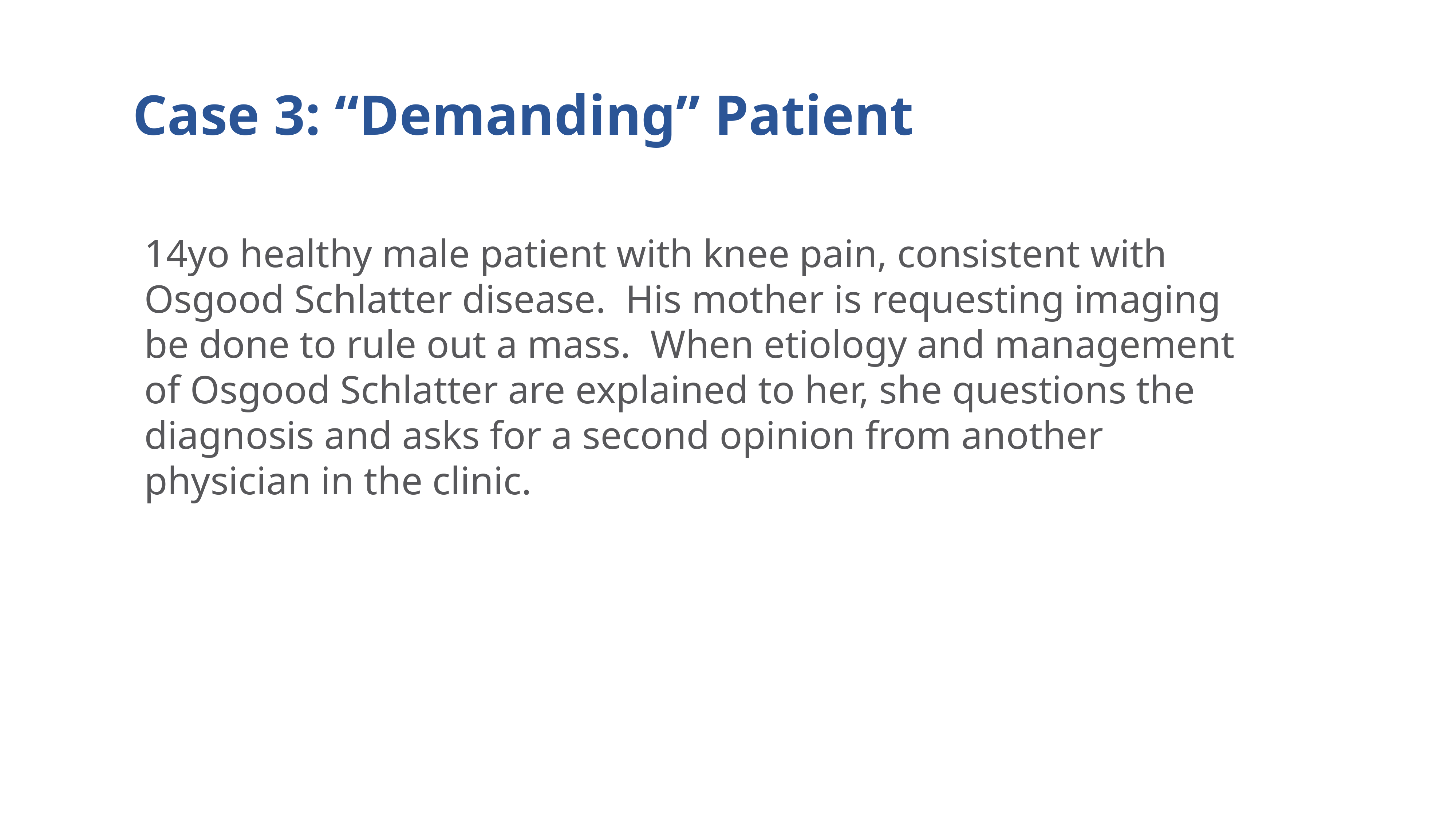

Case 3: “Demanding” Patient
14yo healthy male patient with knee pain, consistent with Osgood Schlatter disease. His mother is requesting imaging be done to rule out a mass. When etiology and management of Osgood Schlatter are explained to her, she questions the diagnosis and asks for a second opinion from another physician in the clinic.

## Slide 26
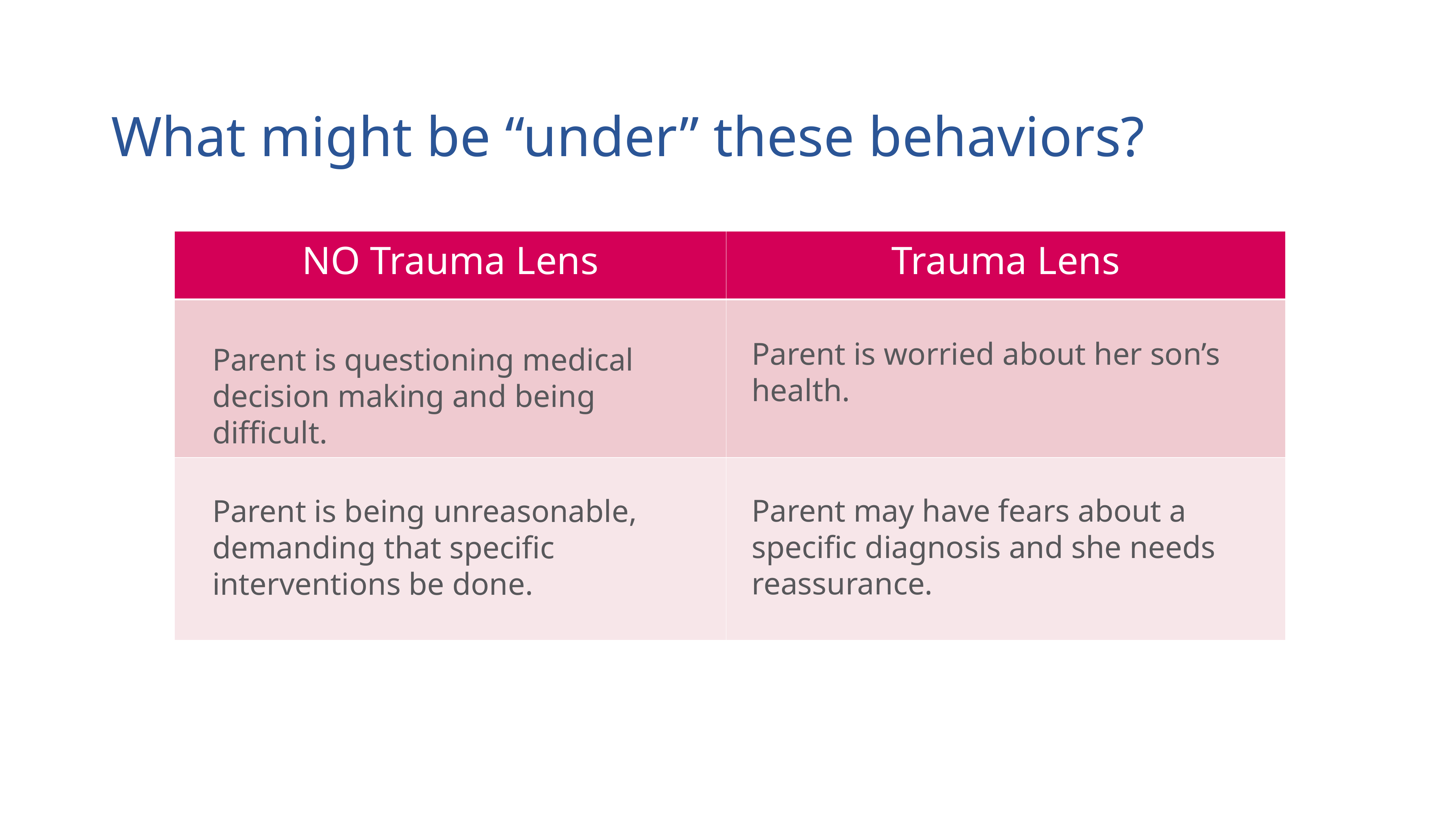

# What might be “under” these behaviors?
| NO Trauma Lens | Trauma Lens |
| --- | --- |
| | |
| | |
Parent is worried about her son’s health.
Parent is questioning medical decision making and being difficult.
Parent may have fears about a specific diagnosis and she needs reassurance.
Parent is being unreasonable, demanding that specific interventions be done.

## Slide 27
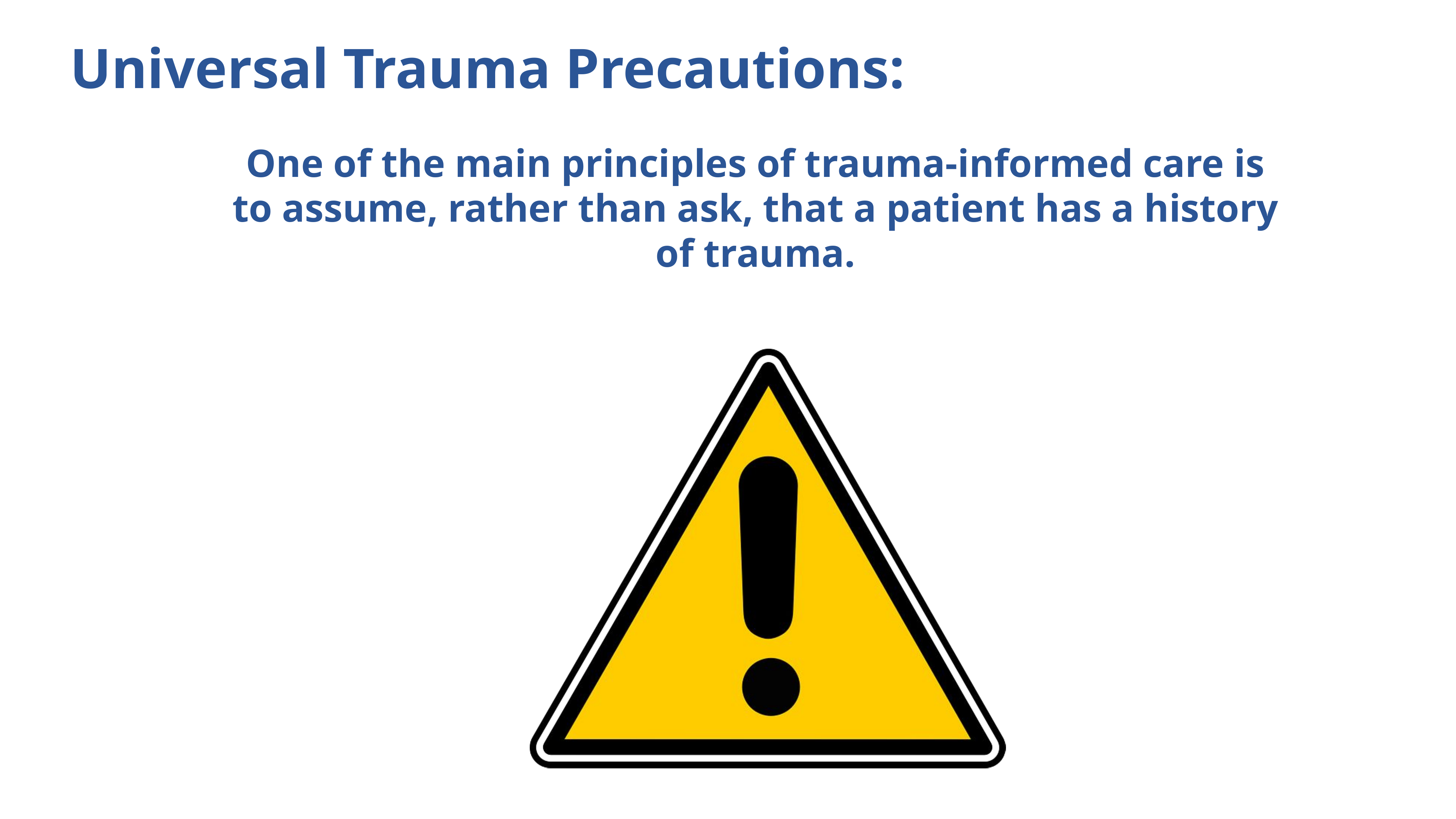

Universal Trauma Precautions:
One of the main principles of trauma-informed care is to assume, rather than ask, that a patient has a history of trauma.

## Slide 28
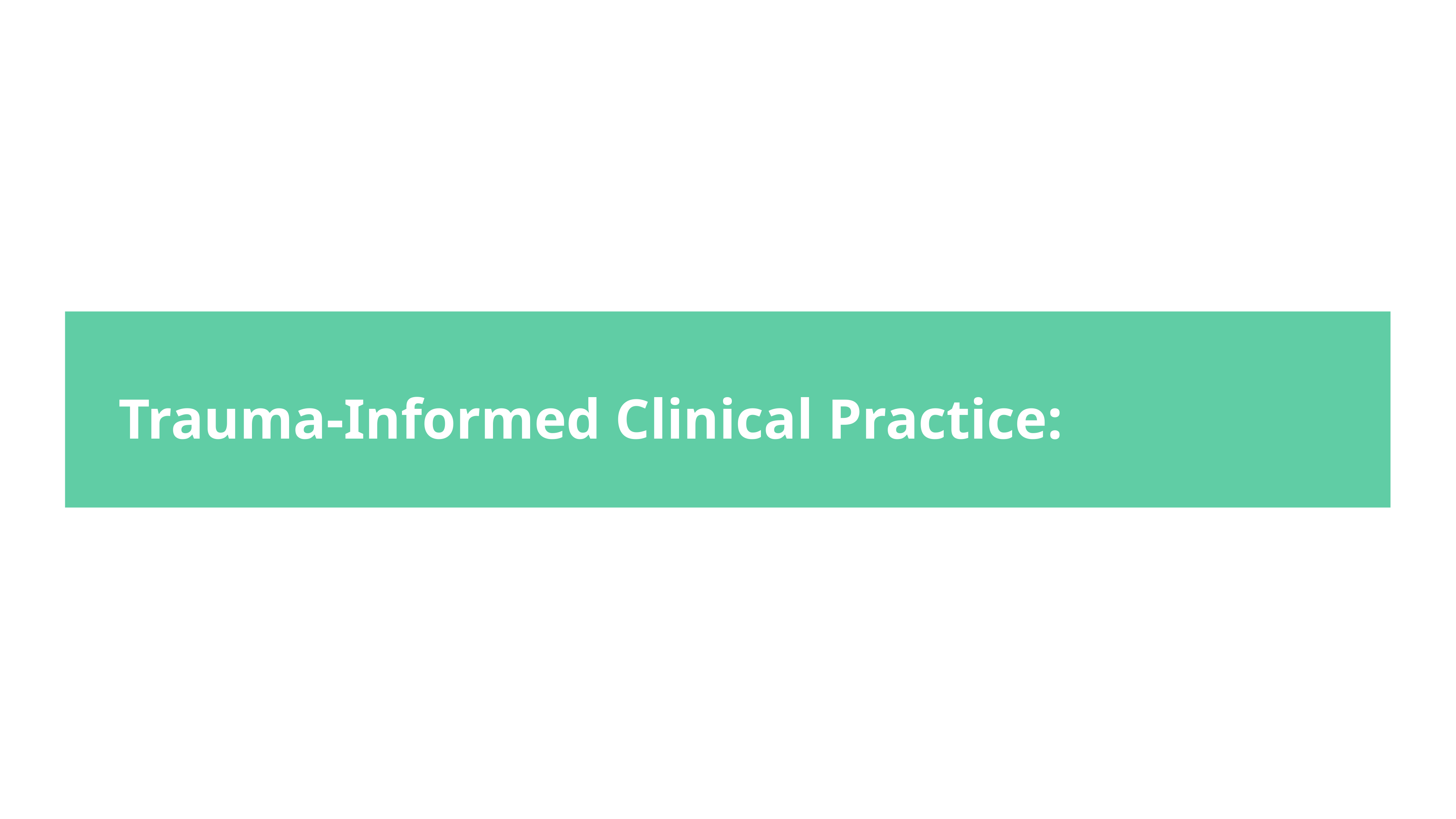

Trauma-Informed Clinical Practice:

## Slide 29
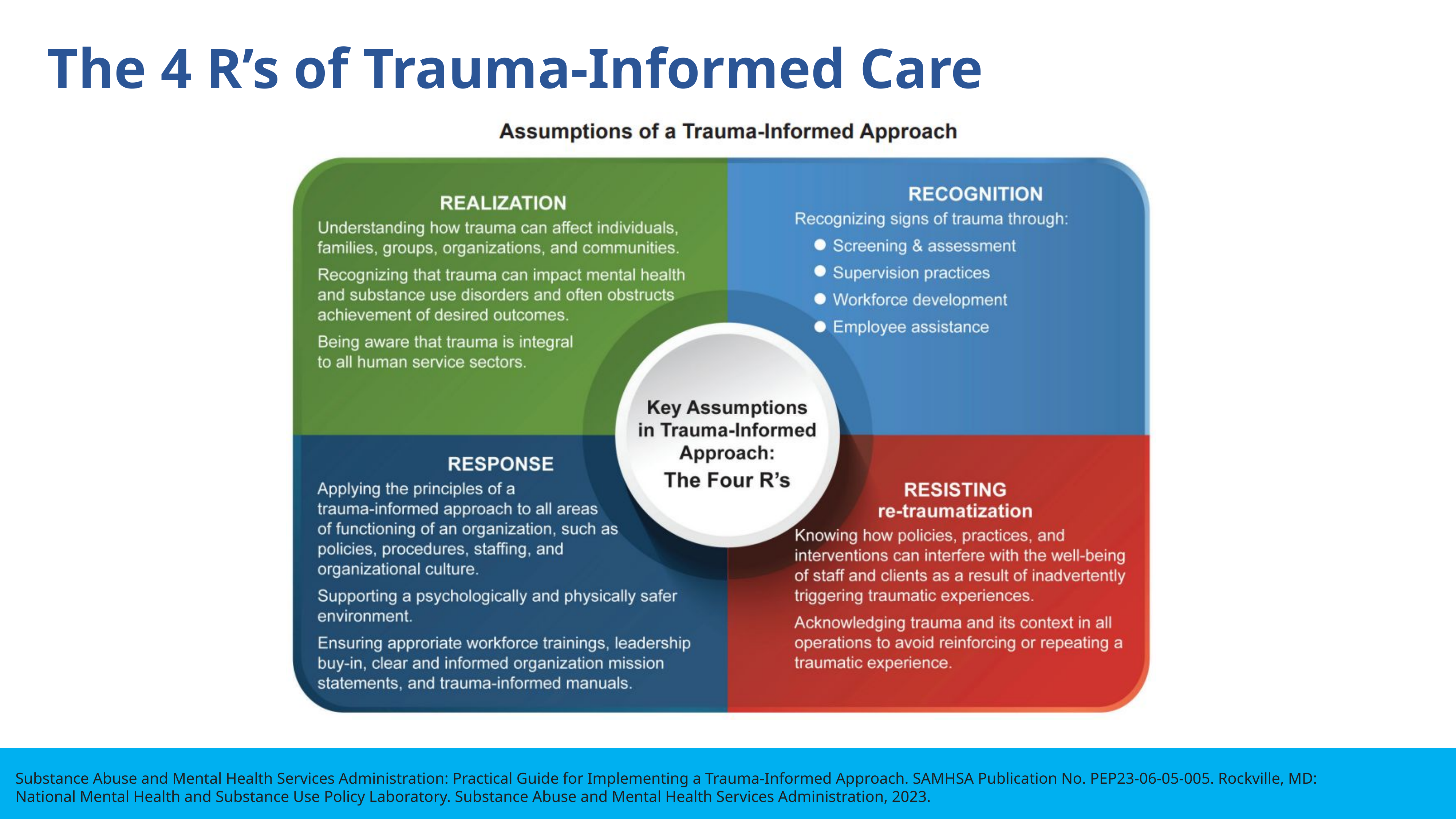

# The 4 R’s of Trauma-Informed Care
Substance Abuse and Mental Health Services Administration: Practical Guide for Implementing a Trauma-Informed Approach. SAMHSA Publication No. PEP23-06-05-005. Rockville, MD: National Mental Health and Substance Use Policy Laboratory. Substance Abuse and Mental Health Services Administration, 2023.

## Slide 30
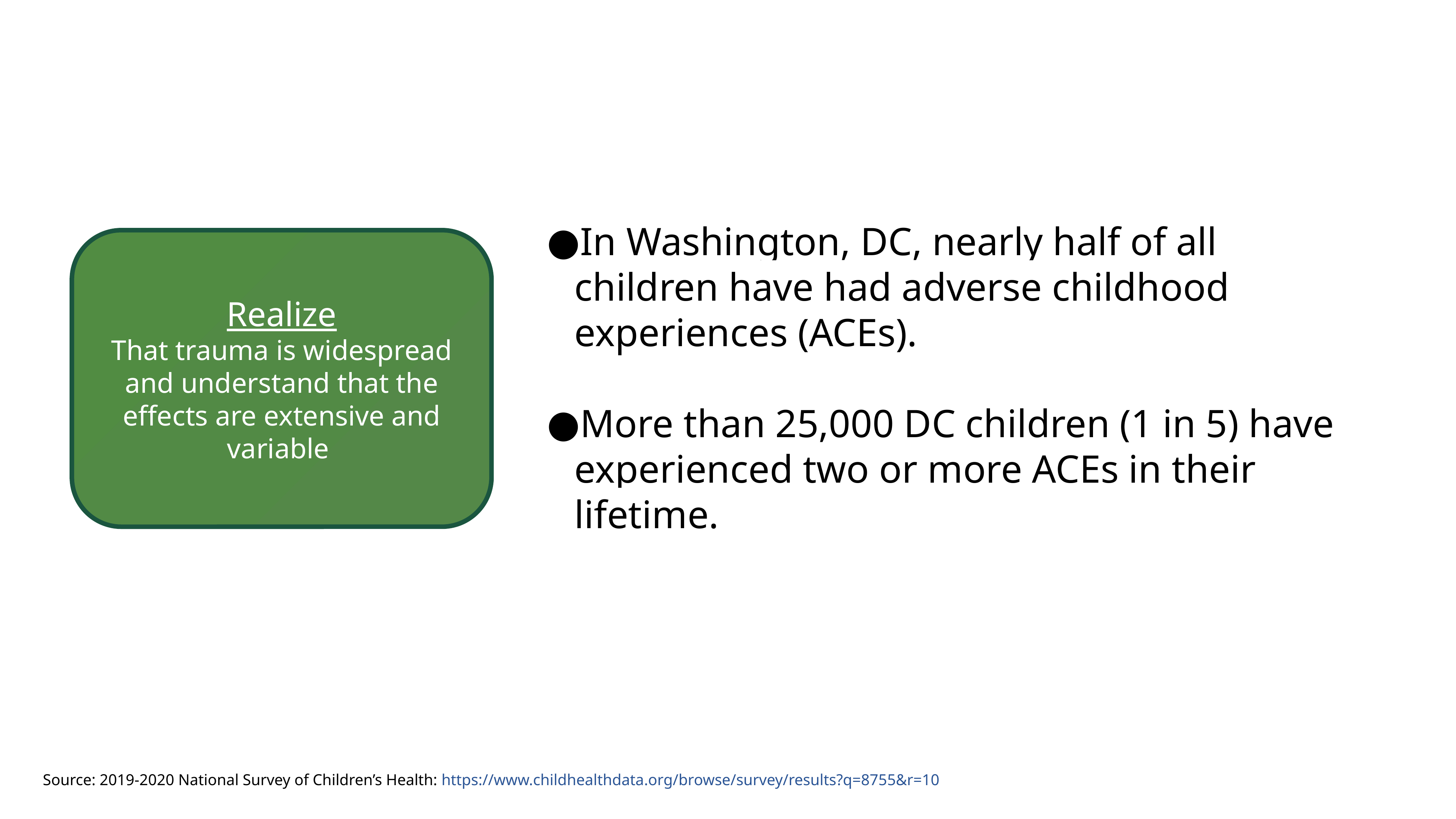

In Washington, DC, nearly half of all children have had adverse childhood experiences (ACEs).
More than 25,000 DC children (1 in 5) have experienced two or more ACEs in their lifetime.
Realize
That trauma is widespread and understand that the effects are extensive and variable
Source: 2019-2020 National Survey of Children’s Health: https://www.childhealthdata.org/browse/survey/results?q=8755&r=10

## Slide 31
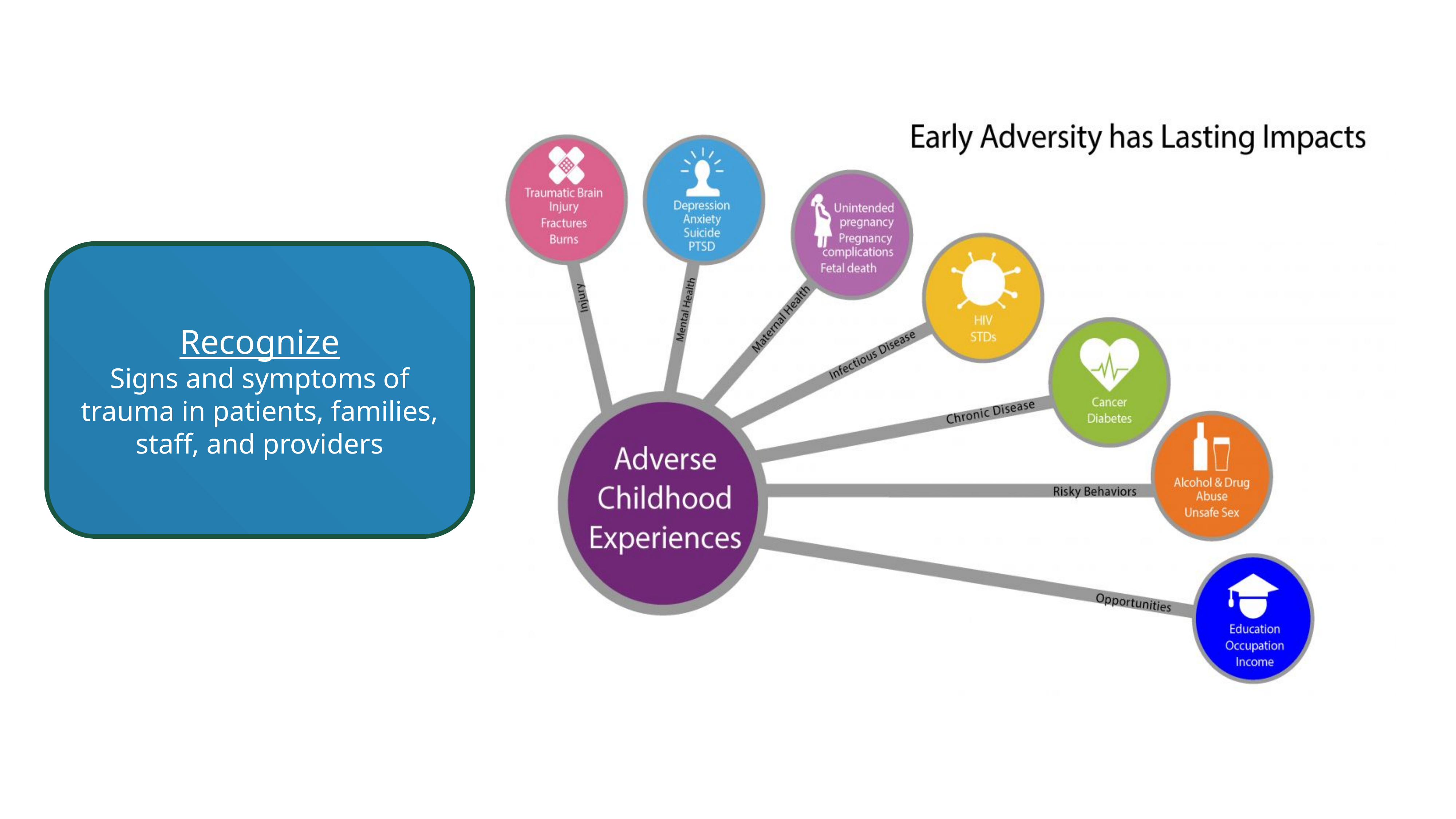

Recognize
Signs and symptoms of trauma in patients, families, staff, and providers

## Slide 32
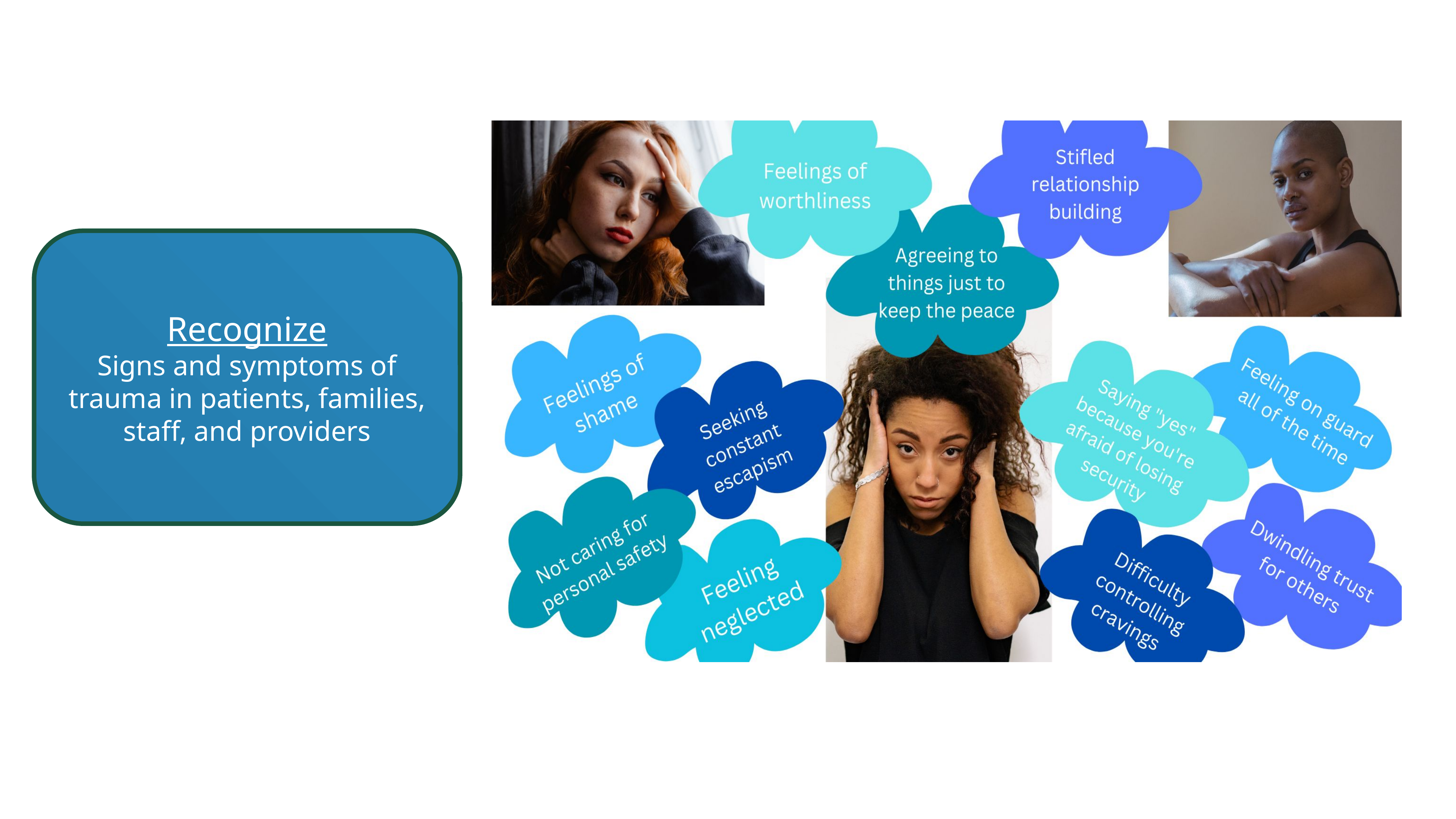

Recognize
Signs and symptoms of trauma in patients, families, staff, and providers

## Slide 33
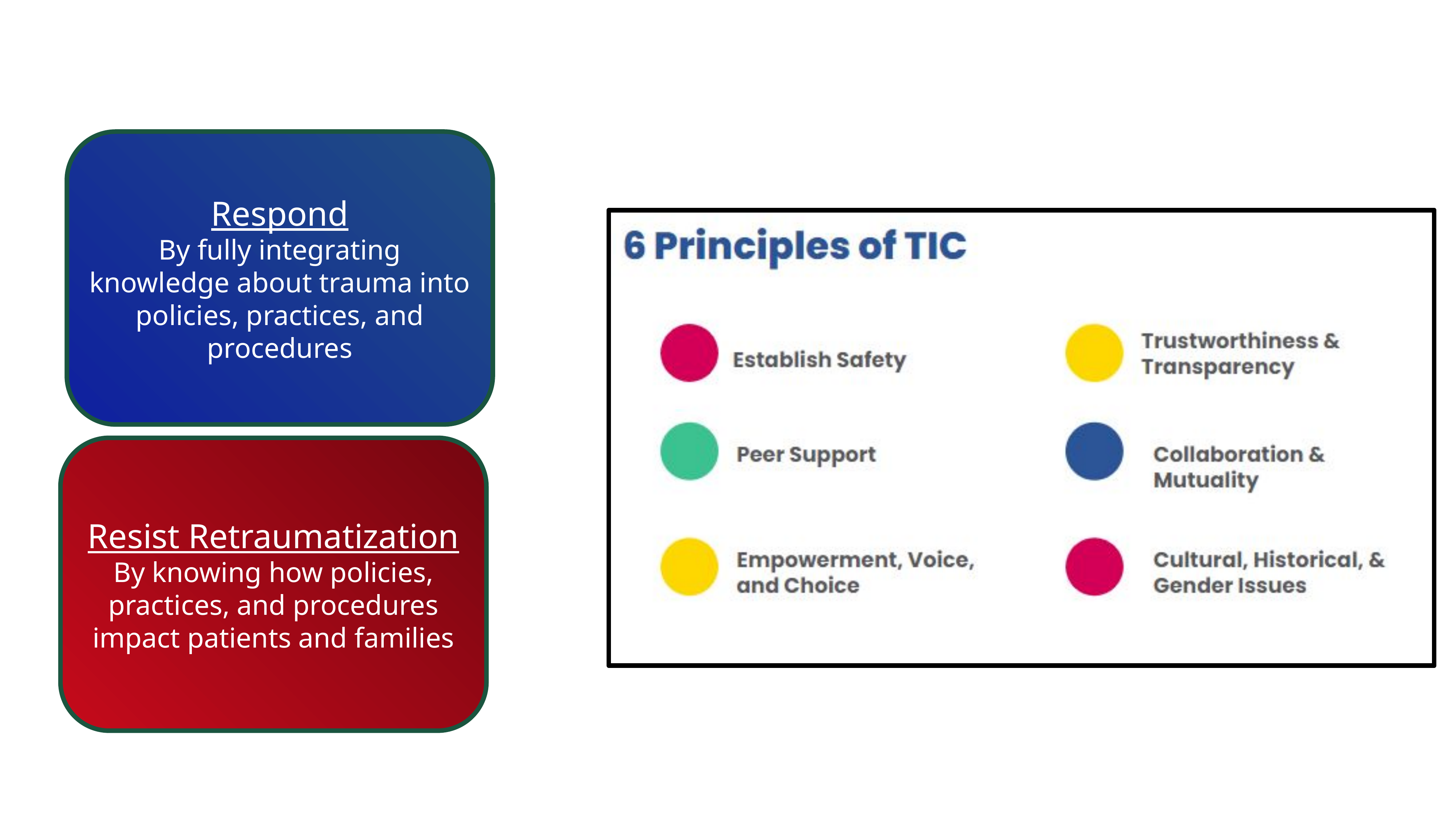

Respond
By fully integrating knowledge about trauma into policies, practices, and procedures
Resist Retraumatization
By knowing how policies, practices, and procedures impact patients and families

## Slide 34
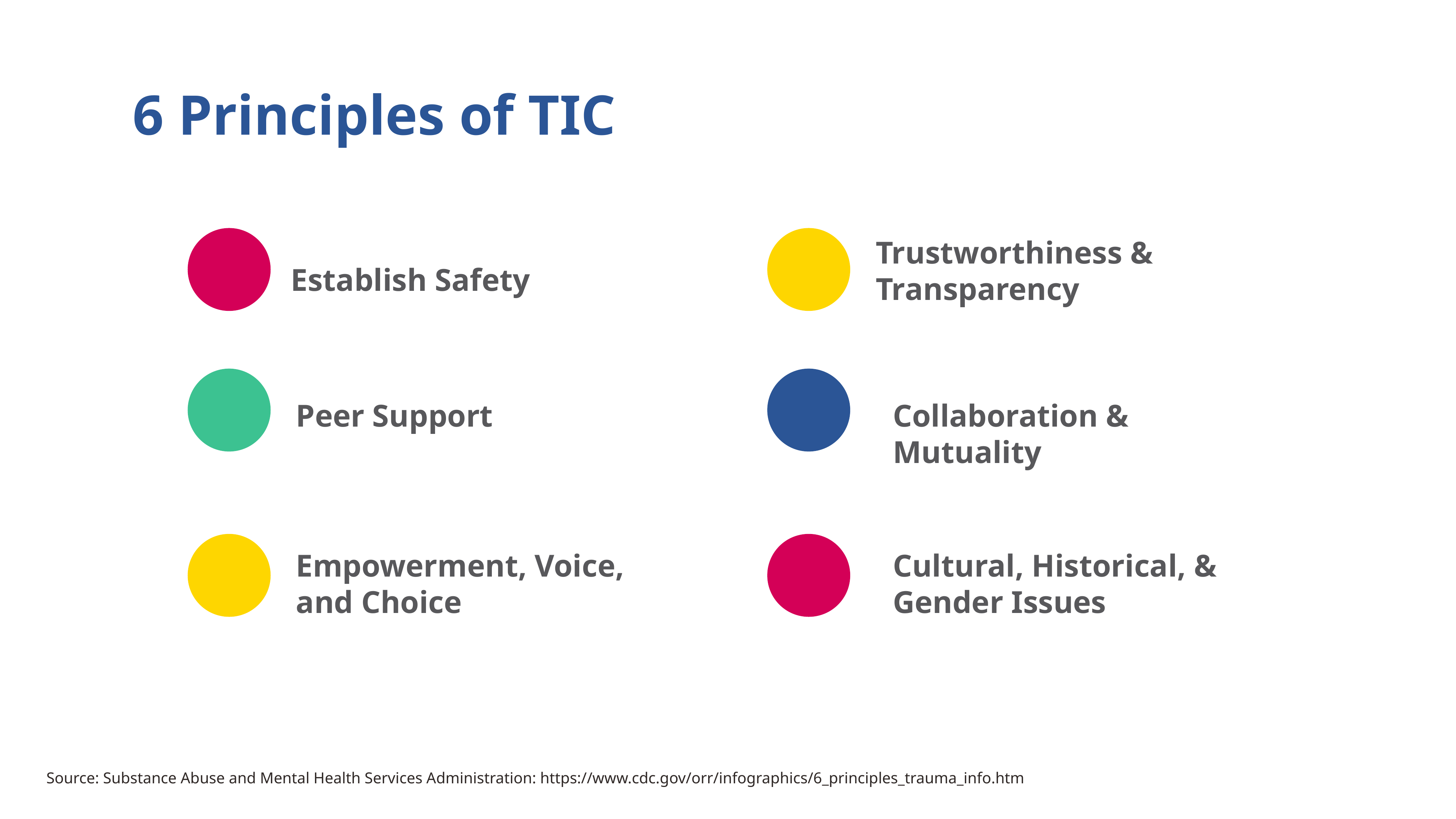

6 Principles of TIC
Trustworthiness & Transparency
Establish Safety
Collaboration & Mutuality
Peer Support
Empowerment, Voice, and Choice
Cultural, Historical, & Gender Issues
Source: Substance Abuse and Mental Health Services Administration: https://www.cdc.gov/orr/infographics/6_principles_trauma_info.htm

## Slide 35
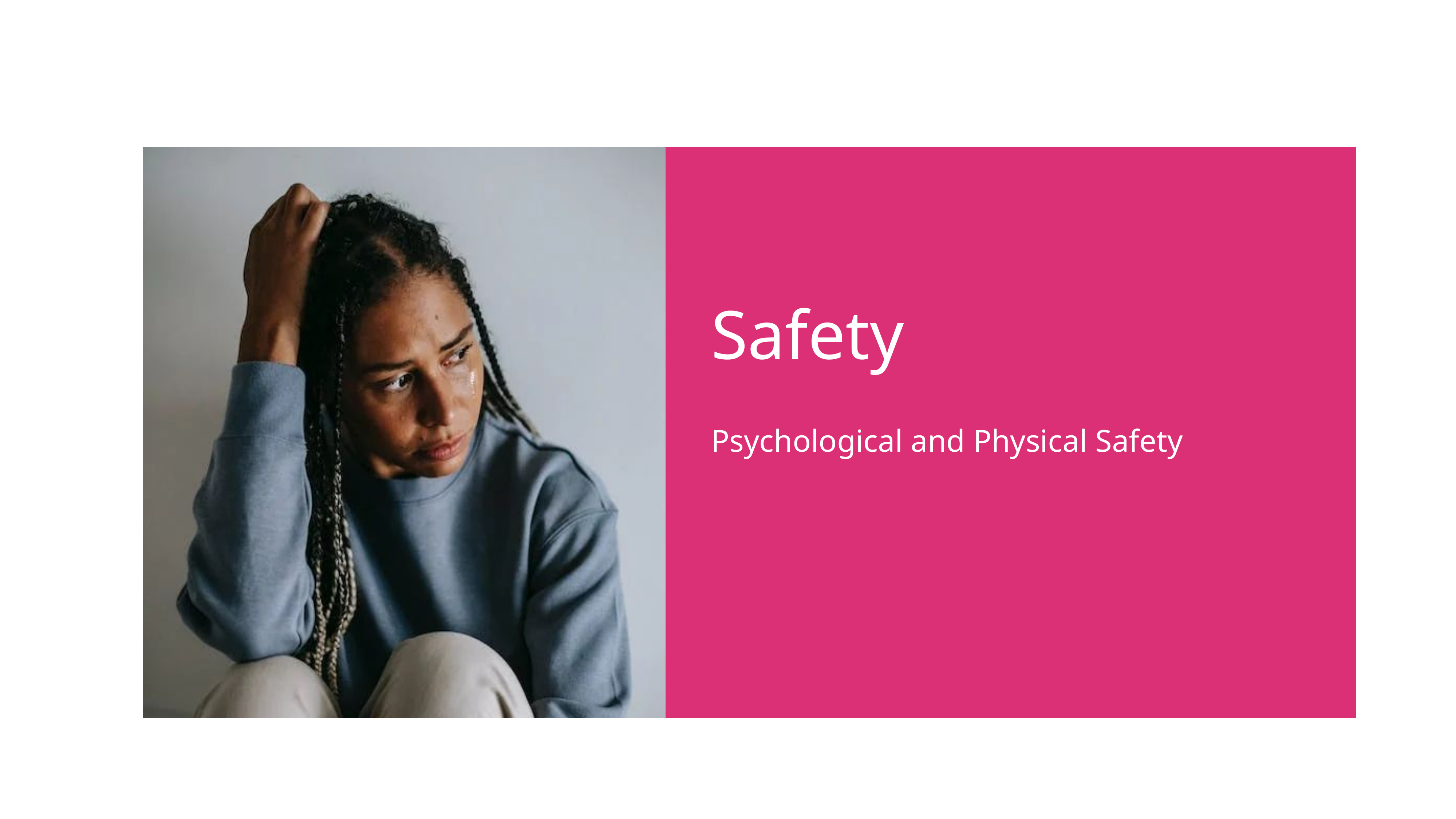

Safety
Psychological and Physical Safety

## Slide 36
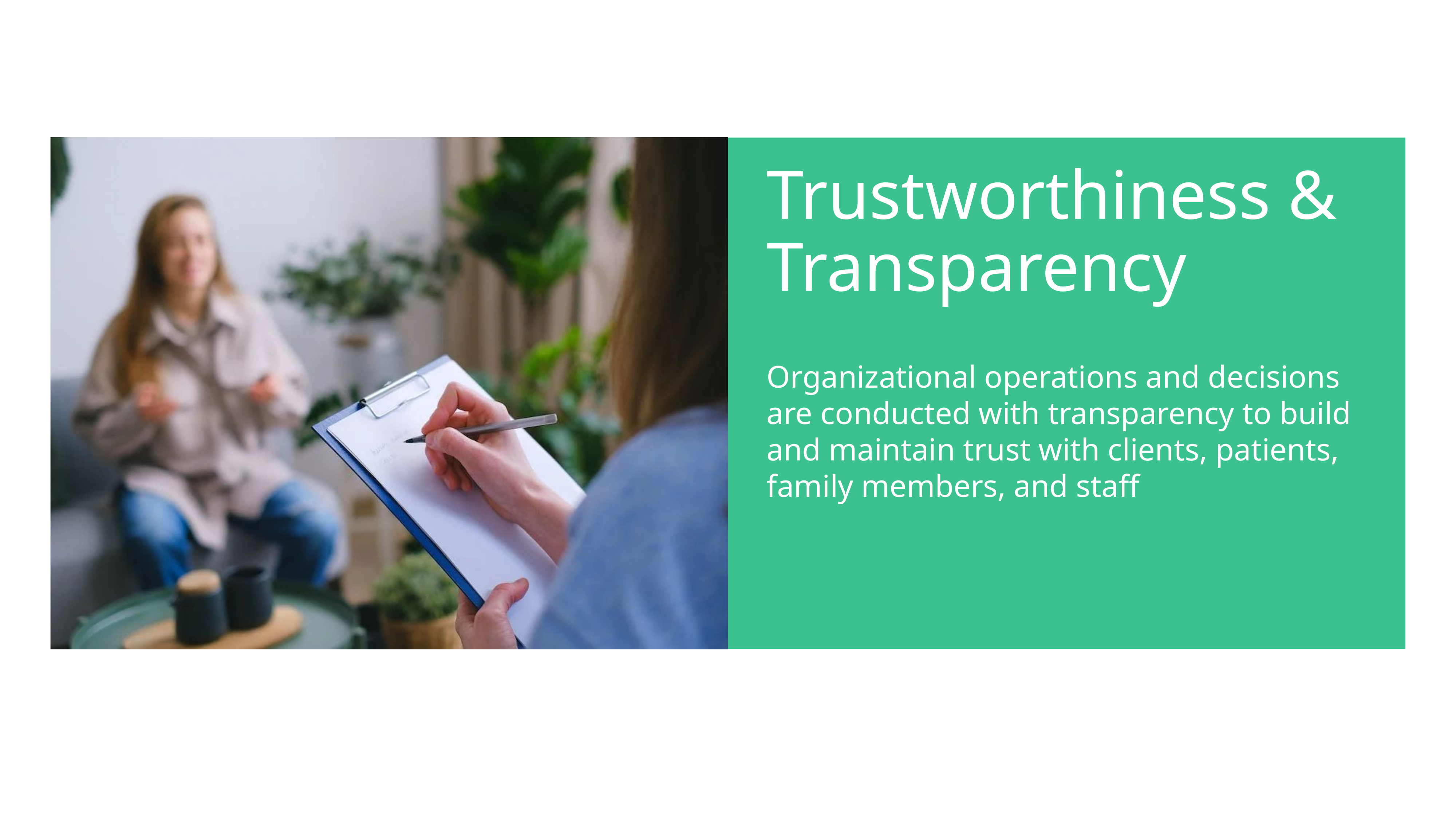

Trustworthiness & Transparency
Organizational operations and decisions are conducted with transparency to build and maintain trust with clients, patients, family members, and staff

## Slide 37
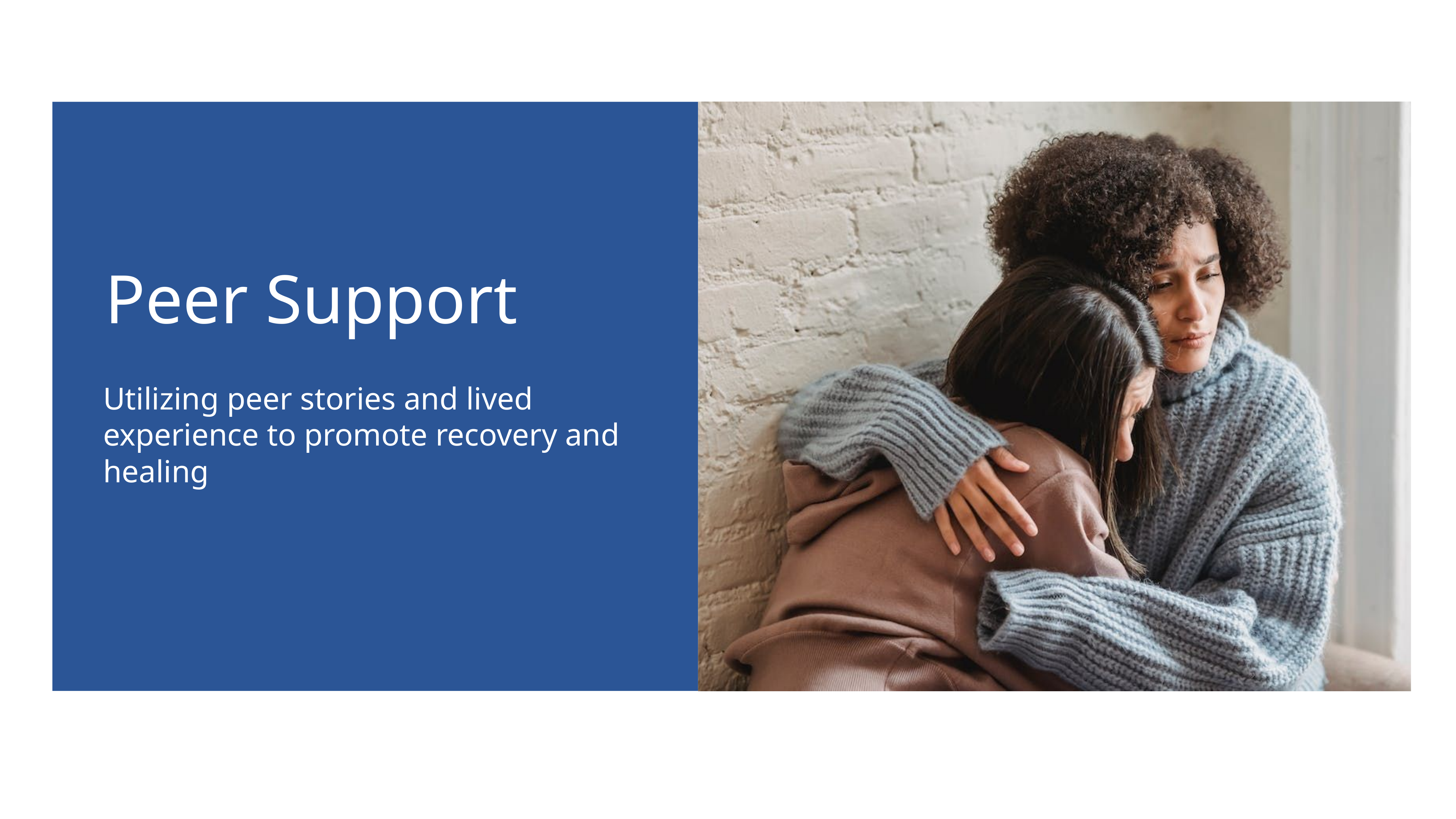

Peer Support
Utilizing peer stories and lived experience to promote recovery and healing

## Slide 38
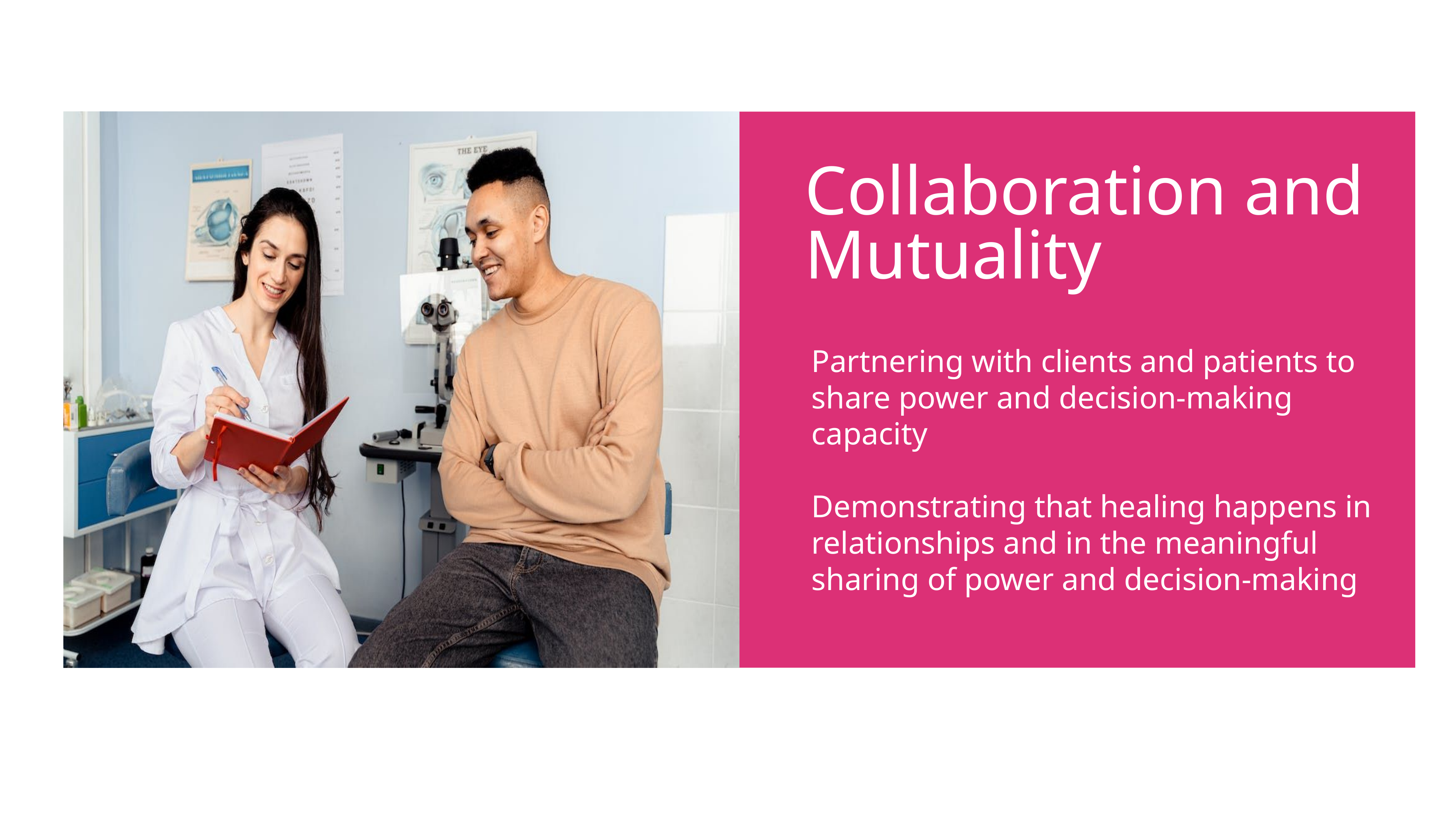

Collaboration and Mutuality
Partnering with clients and patients to share power and decision-making capacity
Demonstrating that healing happens in relationships and in the meaningful sharing of power and decision-making

## Slide 39
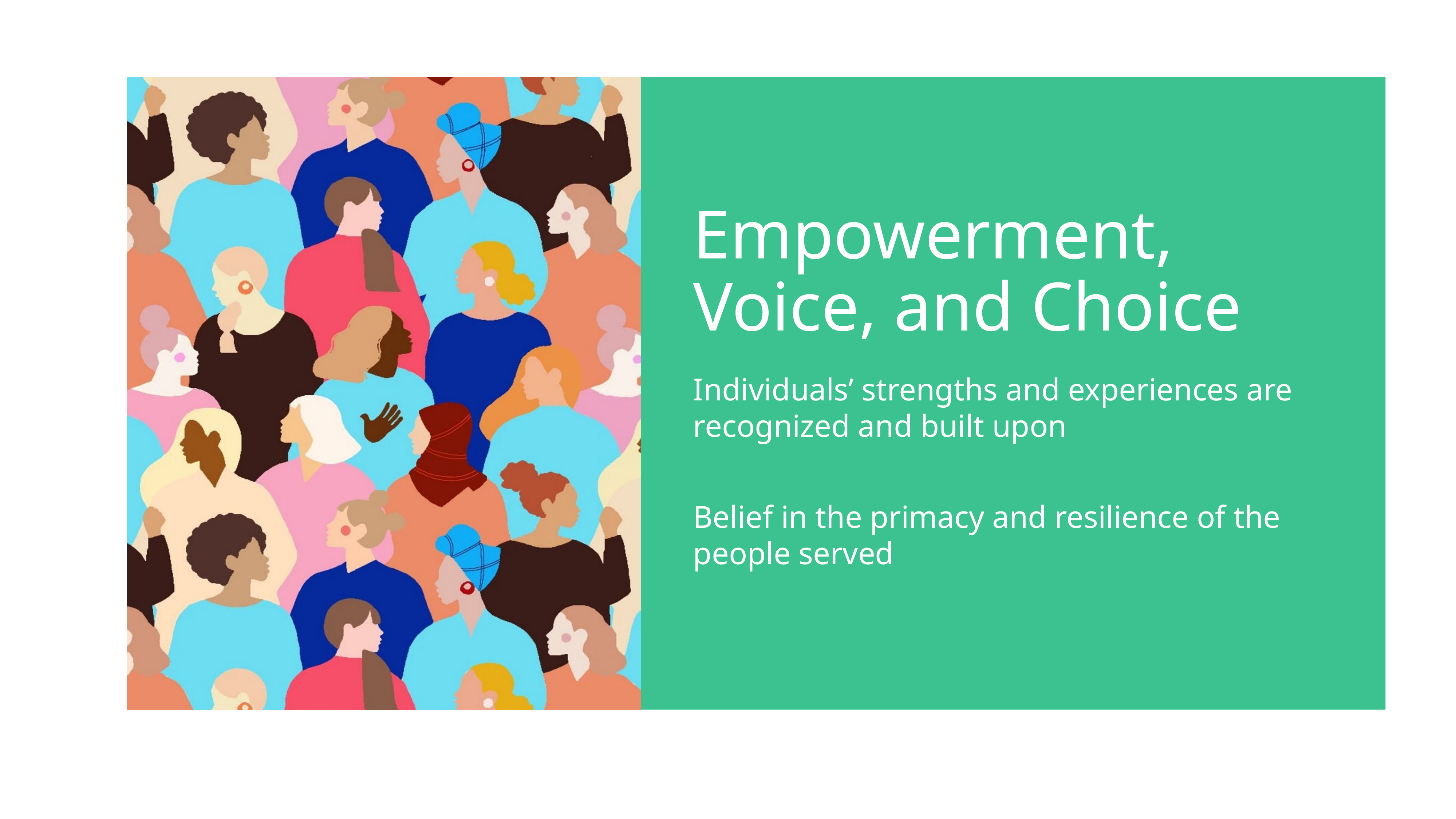

Empowerment, Voice, and Choice
Individuals’ strengths and experiences are recognized and built upon
Belief in the primacy and resilience of the people served

## Slide 40
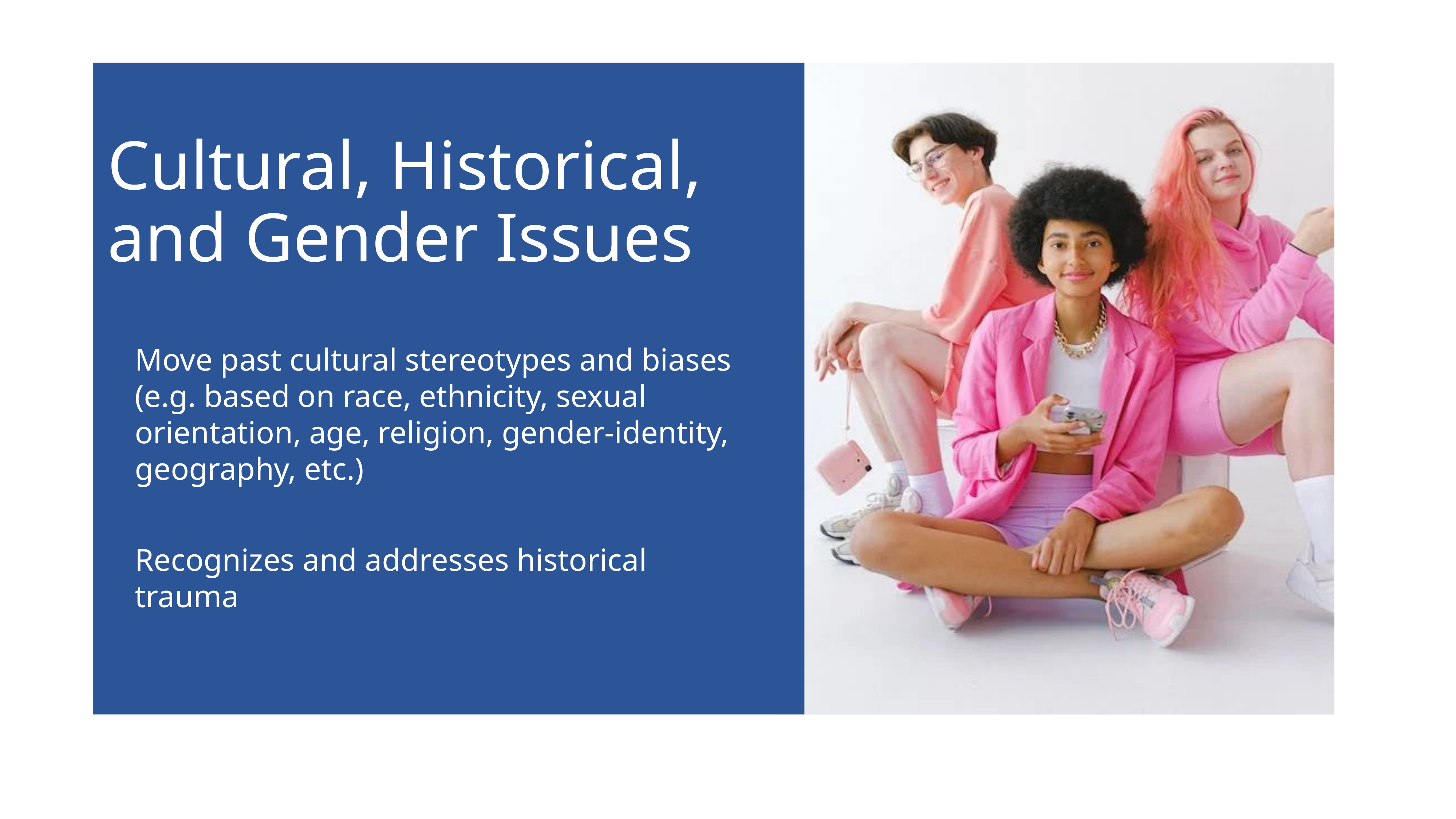

Cultural, Historical, and Gender Issues
Move past cultural stereotypes and biases (e.g. based on race, ethnicity, sexual orientation, age, religion, gender-identity, geography, etc.)
Recognizes and addresses historical trauma

## Slide 41
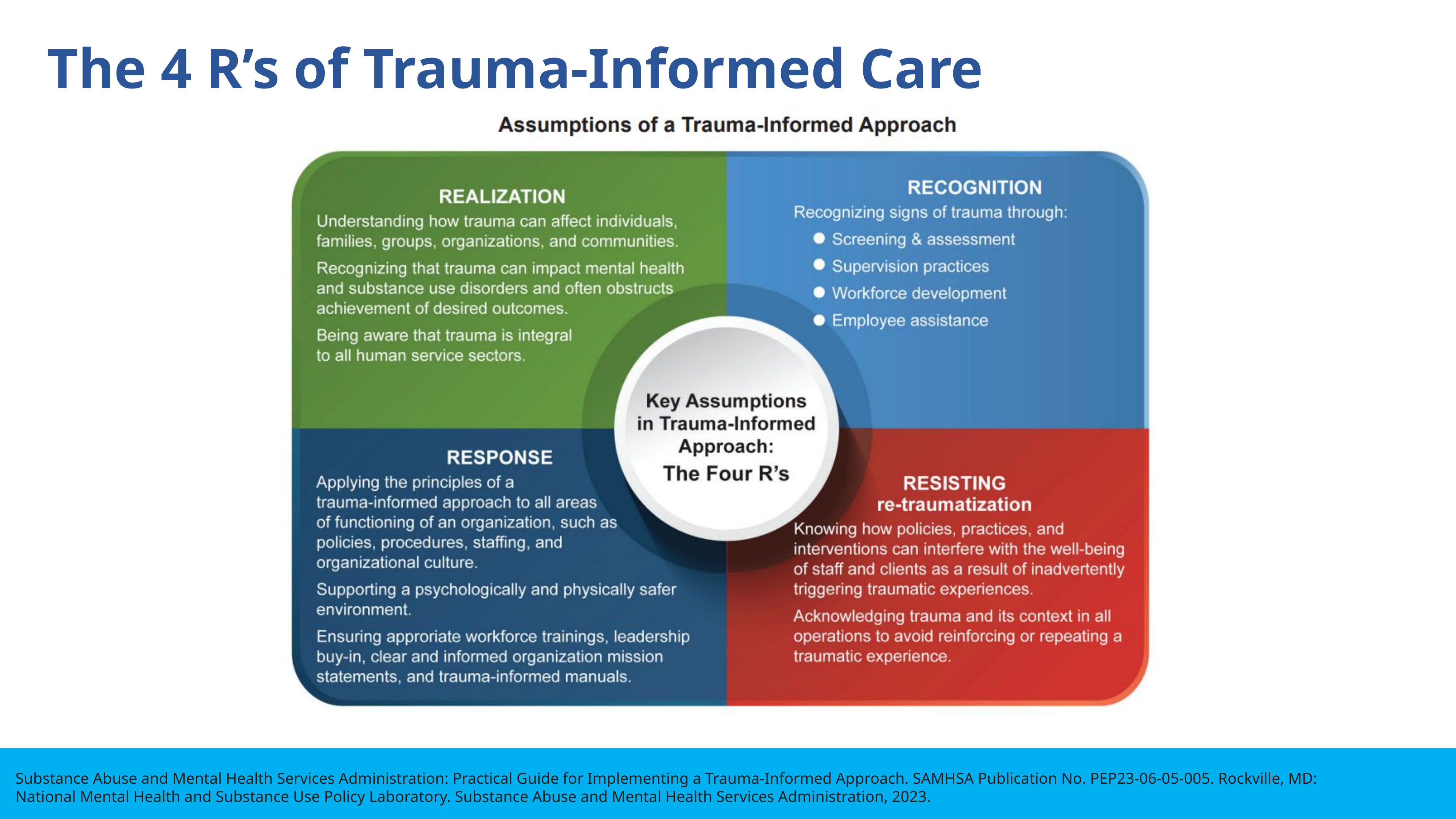

# The 4 R’s of Trauma-Informed Care
Substance Abuse and Mental Health Services Administration: Practical Guide for Implementing a Trauma-Informed Approach. SAMHSA Publication No. PEP23-06-05-005. Rockville, MD: National Mental Health and Substance Use Policy Laboratory. Substance Abuse and Mental Health Services Administration, 2023.

## Slide 42
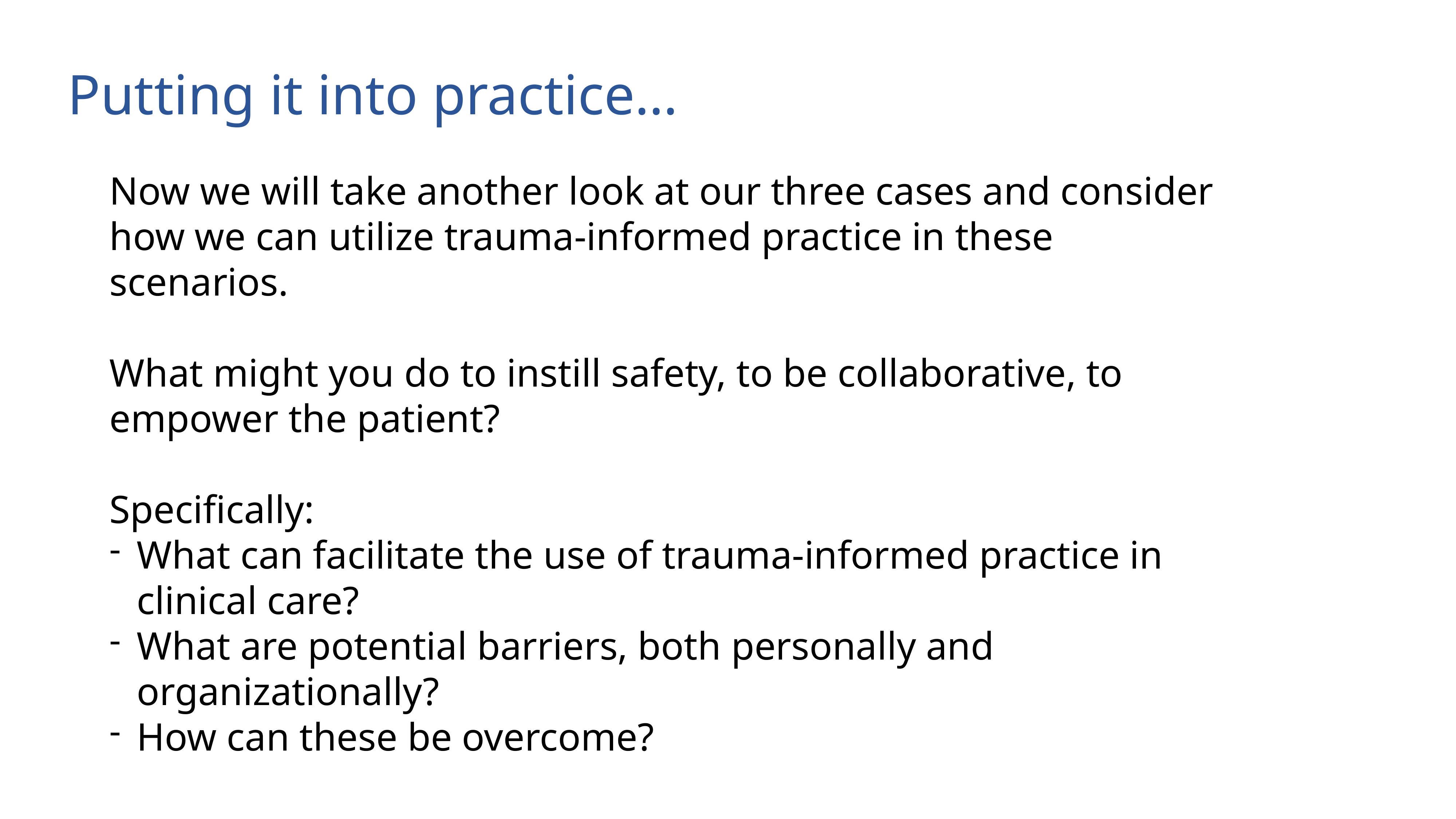

# Putting it into practice…
Now we will take another look at our three cases and consider how we can utilize trauma-informed practice in these scenarios.
What might you do to instill safety, to be collaborative, to empower the patient?
Specifically:
What can facilitate the use of trauma-informed practice in clinical care?
What are potential barriers, both personally and organizationally?
How can these be overcome?

## Slide 43
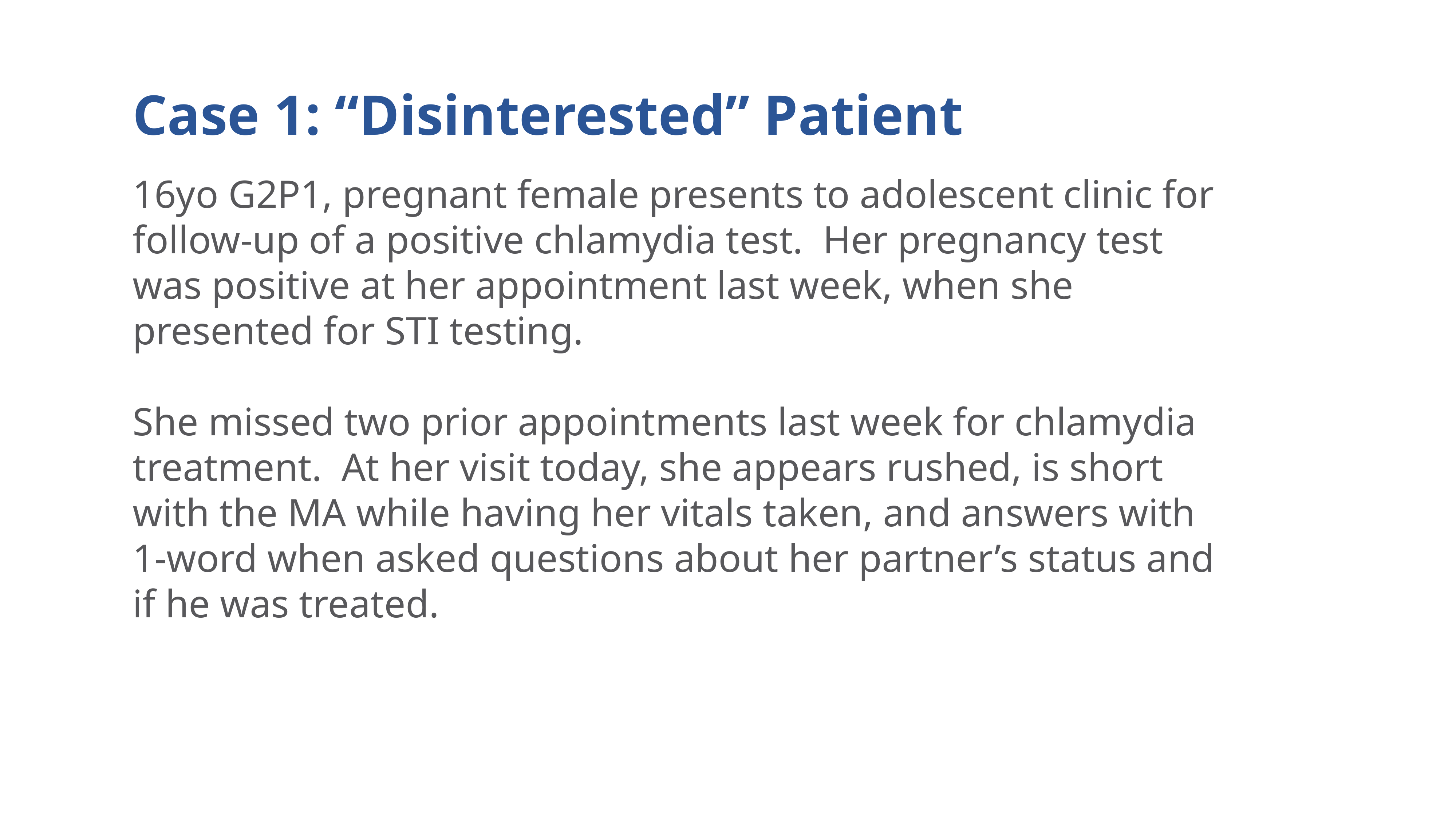

Case 1: “Disinterested” Patient
16yo G2P1, pregnant female presents to adolescent clinic for follow-up of a positive chlamydia test. Her pregnancy test was positive at her appointment last week, when she presented for STI testing.
She missed two prior appointments last week for chlamydia treatment. At her visit today, she appears rushed, is short with the MA while having her vitals taken, and answers with 1-word when asked questions about her partner’s status and if he was treated.

## Slide 44
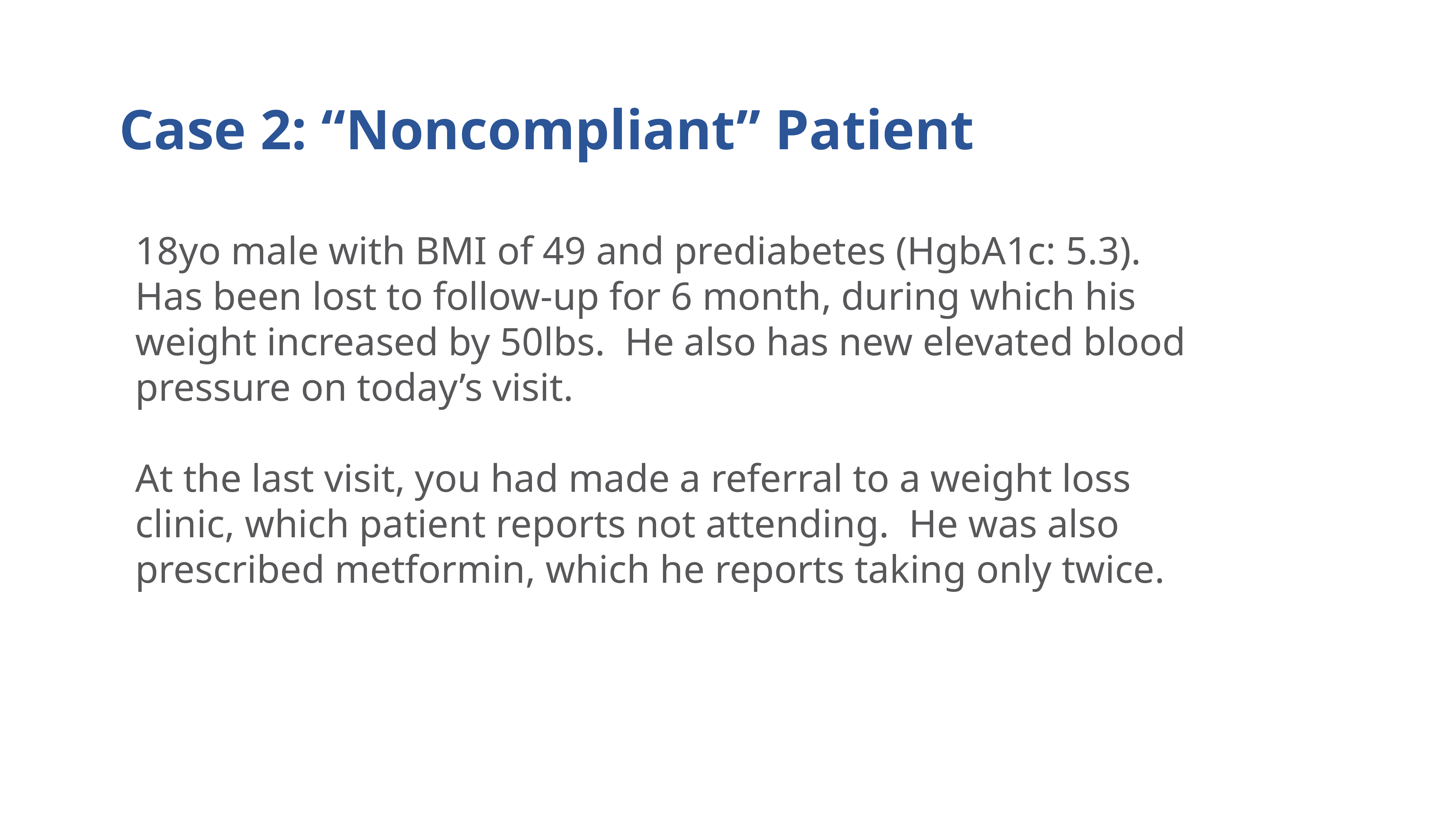

Case 2: “Noncompliant” Patient
18yo male with BMI of 49 and prediabetes (HgbA1c: 5.3). Has been lost to follow-up for 6 month, during which his weight increased by 50lbs. He also has new elevated blood pressure on today’s visit.
At the last visit, you had made a referral to a weight loss clinic, which patient reports not attending. He was also prescribed metformin, which he reports taking only twice.

## Slide 45
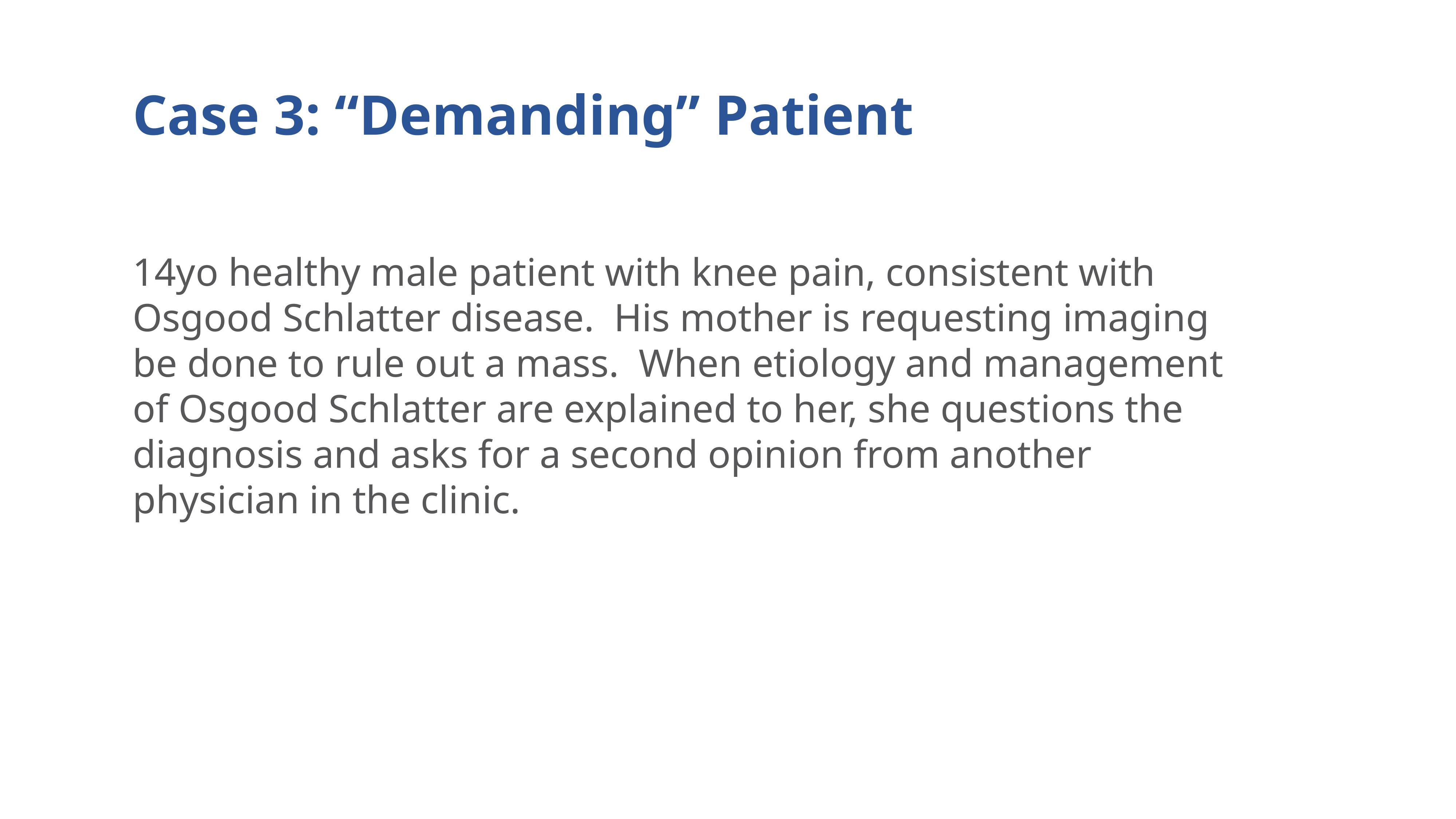

Case 3: “Demanding” Patient
14yo healthy male patient with knee pain, consistent with Osgood Schlatter disease. His mother is requesting imaging be done to rule out a mass. When etiology and management of Osgood Schlatter are explained to her, she questions the diagnosis and asks for a second opinion from another physician in the clinic.

## Slide 46
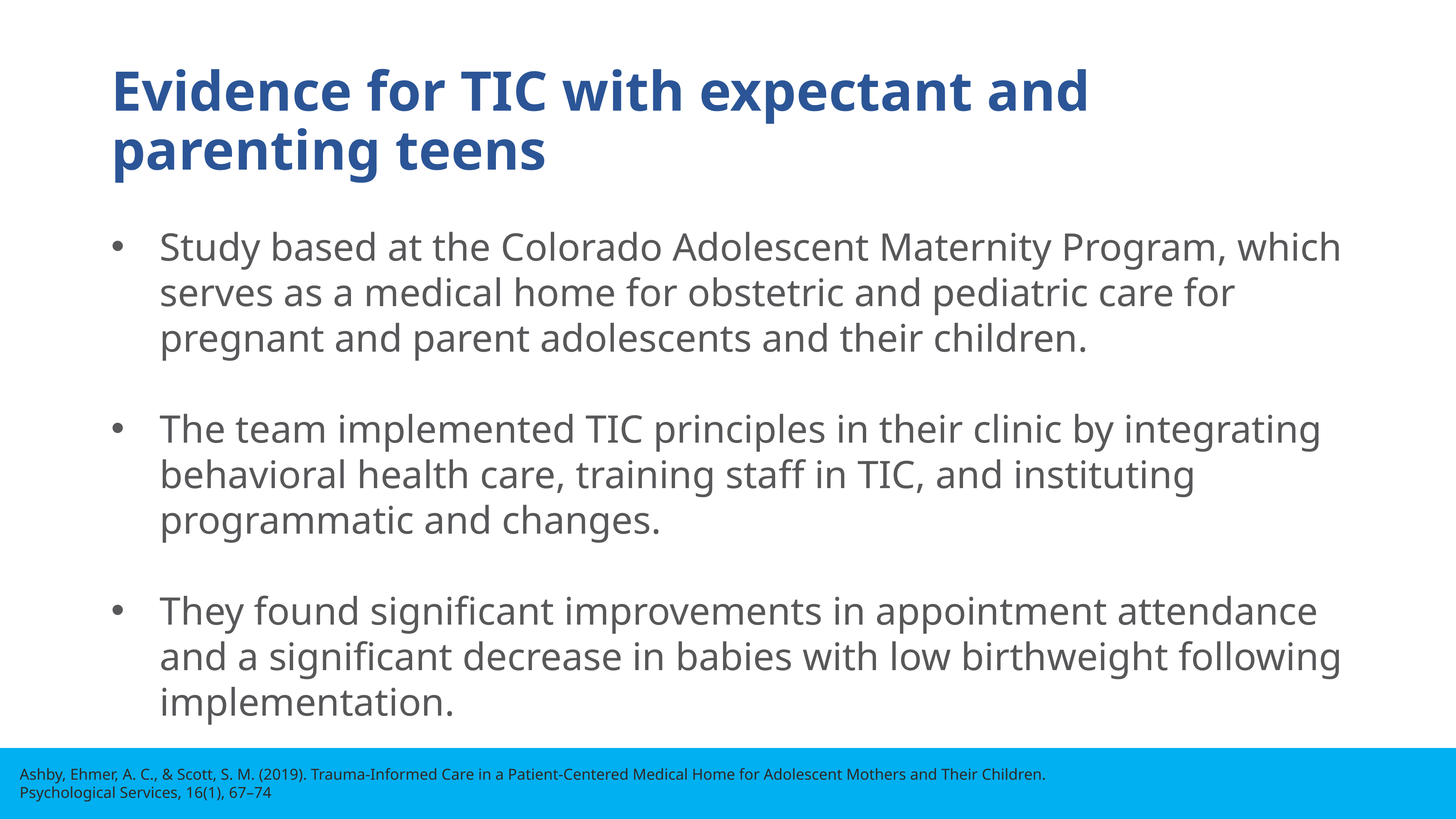

# Evidence for TIC with expectant and parenting teens
Study based at the Colorado Adolescent Maternity Program, which serves as a medical home for obstetric and pediatric care for pregnant and parent adolescents and their children.
The team implemented TIC principles in their clinic by integrating behavioral health care, training staff in TIC, and instituting programmatic and changes.
They found significant improvements in appointment attendance and a significant decrease in babies with low birthweight following implementation.
Ashby, Ehmer, A. C., & Scott, S. M. (2019). Trauma-Informed Care in a Patient-Centered Medical Home for Adolescent Mothers and Their Children. Psychological Services, 16(1), 67–74

## Slide 47
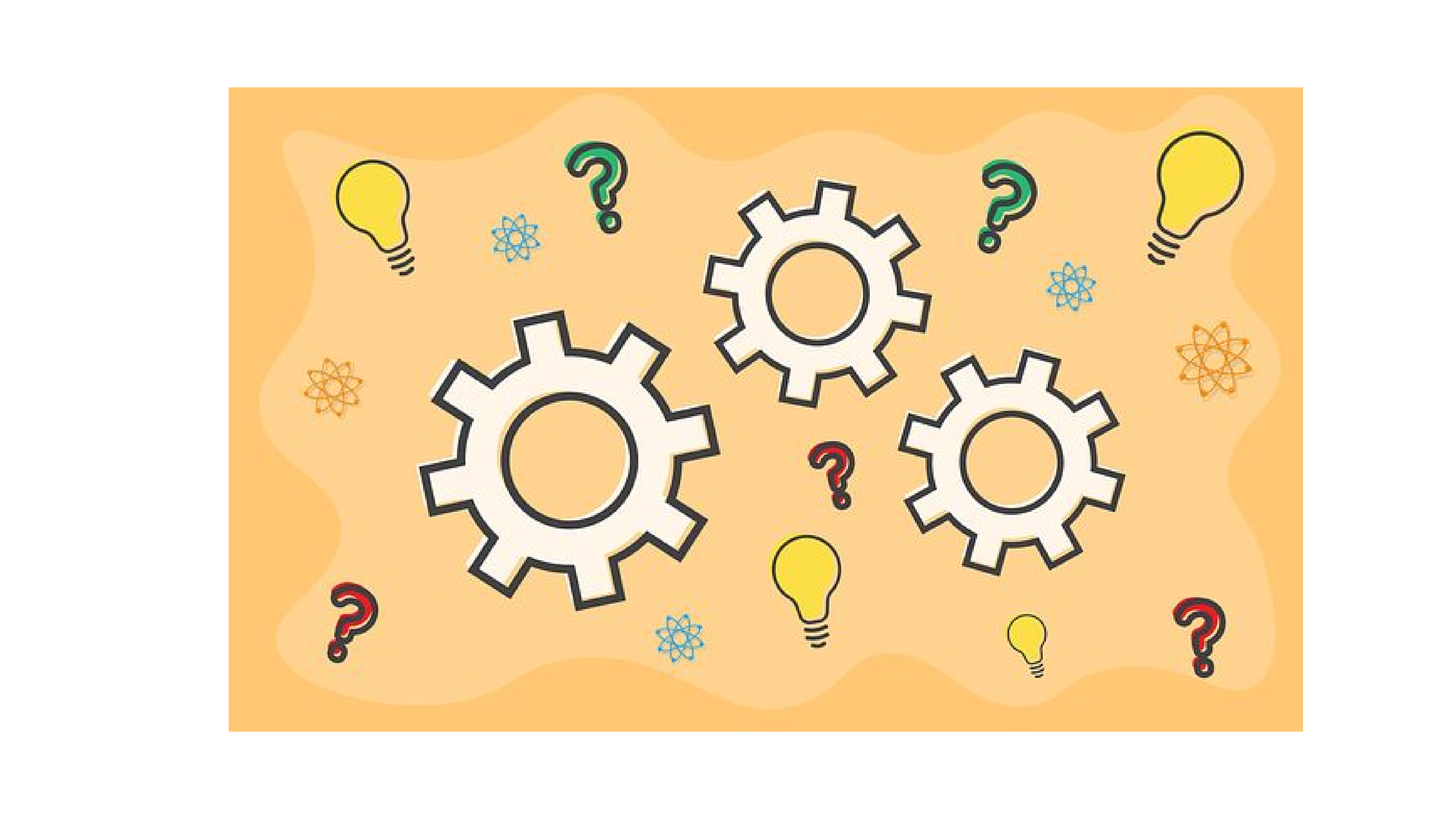

## Slide 48
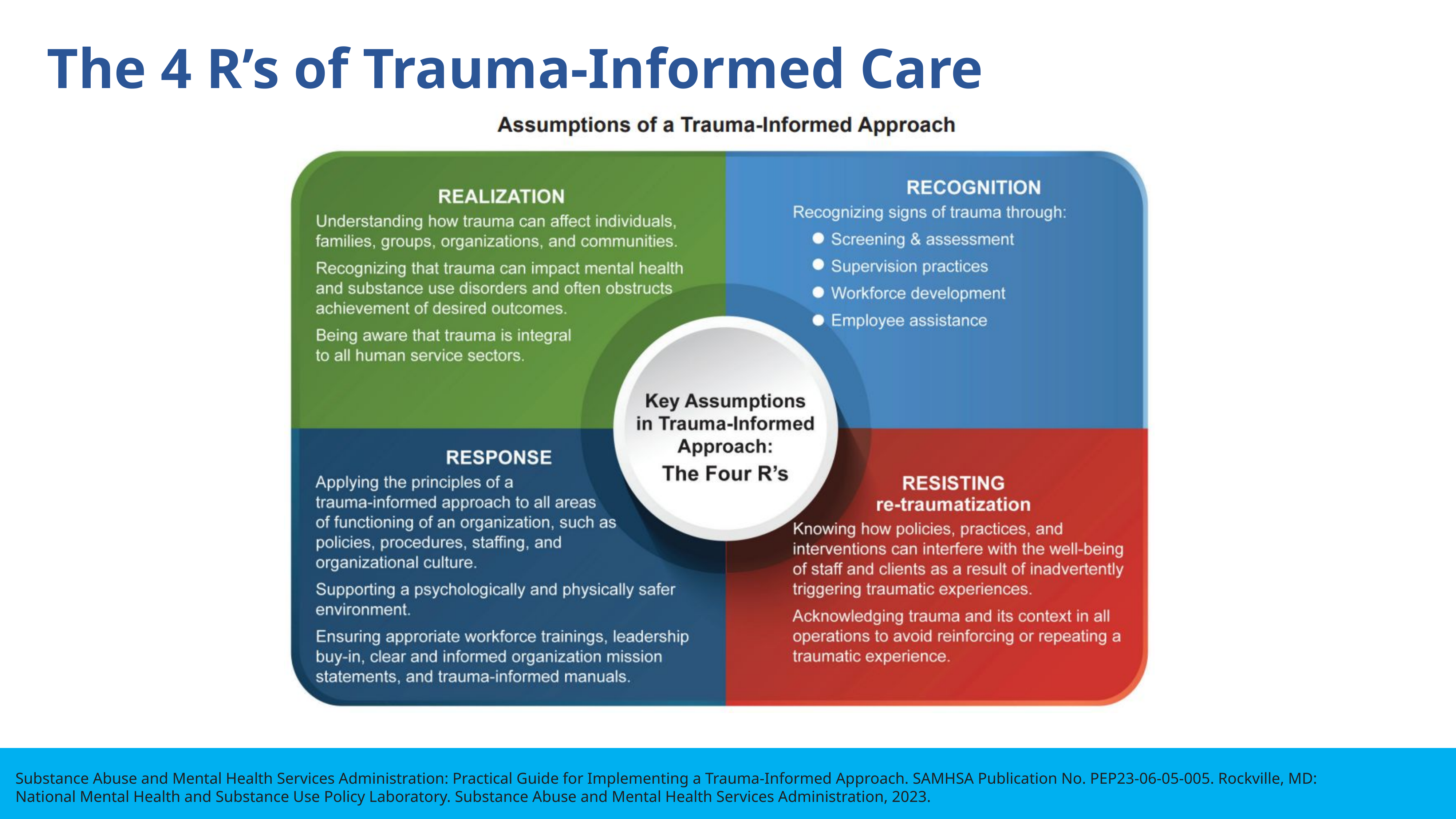

# The 4 R’s of Trauma-Informed Care
Substance Abuse and Mental Health Services Administration: Practical Guide for Implementing a Trauma-Informed Approach. SAMHSA Publication No. PEP23-06-05-005. Rockville, MD: National Mental Health and Substance Use Policy Laboratory. Substance Abuse and Mental Health Services Administration, 2023.
