# Supplementary material for: Applying a Trauma-Informed Lens to Challenging Adolescent Encounters: A Faculty Development Session for Pediatricians
Source: MedEdPORTAL. 2024 May 31;20:11408. doi: 10.15766/mep_2374-8265.11408 (PMC11219089; doi:10.15766/mep_2374-8265.11408)
Supplement: Supplementary file 1 — Facilitator Guide.docxModule Slide Set.pptxPre- and Postsession Survey.docx [file mep_2374-8265.11408-s001.zip › C. Pre- and Postsession Survey.docx]

Trauma-informed Care for Pediatric Providers training session survey

Survey Flow

Unique Identifier (1 Question, p.2)

- Required for each survey completion

Demographics (2 Questions, p.2-3)

- Required for each survey completion

Pre or Post Session Survey (1 Question, p.3)

- Branch point to determine survey questions that follow

Pre-Test Block (3 Questions p.4-6)

- Shown only if participant indicates “Before” above

Post-Test Block (9 Questions p.7-11)

- Shown only if participant indicates “After” above

|  |
| --- |

Unique Identifier- Required

|  |
| --- |

Q1.1 Please use the following to create a unique identifier that will allow us to track survey data anonymously:
1. First letter of the city you were born in
2. Birth month in 2-digit format
3. First 2 letters of the name of your primary/elementary school
4. Number of siblings in 2-digit format
5. Last letter of your middle name (use X if none)
Example: Baltimore, May, Ketcham, none, none= B05KE00X

Demographics- Required

Q2.1 What is your role?

- Medical Student
- Resident Physician
- Fellow
- Attending Physician
- NP, DNP, or PA
- Social Worker
- Other (please describe below)

Q2.2 What is your primary site of clinical practice?

- Private Practice
- Academic Center
- Federally Qualified Health Center
- School Based Health Center
- Other (please specify below)

Pre-Test Block

Q3.2 Knowledge: Please use the scale provided to rate your current level of knowledge on the following topics:

|  | Not knowledgeable at all (1) | Slightly knowledgeable (2) | Moderately knowledgeable (3) | Very knowledgeable (4) | Extremely knowledgeable (5) |
| --- | --- | --- | --- | --- | --- |
| The link between traumatic exposures and poor health outcomes |  |  |  |  |  |
| The relationship between traumatic exposures and coping behaviors |  |  |  |  |  |
| The central tenet of trauma-informed care |  |  |  |  |  |
| The six principles of trauma-informed care |  |  |  |  |  |
| The concept of universal trauma precautions |  |  |  |  |  |

Q3.3 Practice: Rate how often you do the following:

|  | Never (1) | Sometimes (2) | About half the time (3) | Most of the time (4) | Always (5) |
| --- | --- | --- | --- | --- | --- |
| Utilize the central tenet of trauma-informed care when confronted with a challenging patient interaction |  |  |  |  |  |
| Incorporate a trauma-informed approach in your routine patient interactions |  |  |  |  |  |
| Incorporate a trauma-informed approach in the care of pregnant and parenting adolescents |  |  |  |  |  |
| Incorporate a trauma-informed approach in the care of patients with obesity |  |  |  |  |  |

Q3.4 Confidence: Rate your confidence with the following:

|  | Not Confident (1) | A Little Confident (2) | Somewhat Confident (3) | Confident (4) | Very Confident (5) |
| --- | --- | --- | --- | --- | --- |
| The application of a trauma-informed lens to patient care interactions |  |  |  |  |  |
| The application of the six principles of trauma-informed care to patient care interactions |  |  |  |  |  |

End of Block: Pre-Test Block

Start of Block: Post-Test Block

Q4.1 Knowledge: After completing this module, how would you now rate your knowledge on the following topics:

|  | Not knowledgeable at all (1) | Slightly knowledgeable (2) | Moderately knowledgeable (3) | Very knowledgeable (4) | Extremely knowledgeable (5) |
| --- | --- | --- | --- | --- | --- |
| The link between traumatic exposures and poor health outcomes |  |  |  |  |  |
| The relationship between traumatic exposures and coping behaviors |  |  |  |  |  |
| The central tenet of trauma-informed care |  |  |  |  |  |
| The six principles of trauma-informed care |  |  |  |  |  |
| The concept of universal trauma precautions |  |  |  |  |  |

Q4.2 Practice: After completing this module, how often will you do the following:

|  | Never (1) | Sometimes (2) | About half the time (3) | Most of the time (4) | Always (5) |
| --- | --- | --- | --- | --- | --- |
| Utilize the central tenet of trauma-informed care when confronted with a challenging patient interaction |  |  |  |  |  |
| Incorporate a trauma-informed approach in your routine patient interactions |  |  |  |  |  |
| Incorporate a trauma-informed approach in the care of pregnant and parenting adolescents |  |  |  |  |  |
| Incorporate a trauma-informed approach in the care of patients with obesity |  |  |  |  |  |

Q4.3 Confidence: After completing this module, please rate your confidence with the following:

|  | Not Confident (1) | A Little Confident (2) | Somewhat Confident (3) | Confident (4) | Very Confident (5) |
| --- | --- | --- | --- | --- | --- |
| The application of a trauma-informed lens to patient care interactions |  |  |  |  |  |
| The application of the six principles of trauma-informed care to patient care interactions |  |  |  |  |  |

Q4.4 Please rate the overall quality of this module:

- Terrible (1)
- Poor (2)
- Average (3)
- Good (4)
- Excellent (5)

Q4.5 Rate your agreement with the following statements:

|  | Strongly disagree (1) | Somewhat disagree (2) | Neither agree nor disagree (3) | Somewhat agree (4) | Strongly agree (5) |
| --- | --- | --- | --- | --- | --- |
| This topic is relevant to my learning/practice. |  |  |  |  |  |
| The presenter was effective at facilitating my learning. |  |  |  |  |  |

Q4.6 Please provide any suggestions you have on ways to improve this course.

________________________________________________________________

Q4.7 Please tell us about anything that you felt was helpful or enhanced your learning in this course.

________________________________________________________________

Q4.8 Please list 2 specific learning points that you will take away from this course:

________________________________________________________________

Q4.9 Please list 2 specific changes that you plan to make in your clinical practice as a result of this course:

________________________________________________________________

End of Block: Post-Test Block
