# Supplementary material for: Applying a Trauma-Informed Lens to Challenging Adolescent Encounters: A Faculty Development Session for Pediatricians
Source: MedEdPORTAL. 2024 May 31;20:11408. doi: 10.15766/mep_2374-8265.11408 (PMC11219089; doi:10.15766/mep_2374-8265.11408)
Supplement: Supplementary file 1 — Facilitator Guide.docxModule Slide Set.pptxPre- and Postsession Survey.docx [file mep_2374-8265.11408-s001.zip › A. Facilitator Guide.docx]

**Appendix A: Facilitator Guide**

*Applying a Trauma-Informed Lens to Challenging Adolescent Encounters: A Faculty Development Session for Pediatricians*

This guide should be shared with all facilitators and can be used to support planning and delivery of the content.

**Objectives**

1. Describe the association between traumatic exposures and health outcomes.

2. Demonstrate how a trauma-informed lens can be applied to challenging patient encounters.

3. Discuss how to utilize trauma-informed practice in challenging patient encounters.

**Session Setting and Delivery**

We conducted the session over the Zoom video conference platform during a regularly scheduled conference time for pediatricians at our institution. We had one presenter share and advance slides throughout the presentation. We utilized PollEverywhere, an audience response polling system that allowed interactive participation during the case-based learning. This module can also be adapted for in-person delivery or for use of virtual breakout sessions; see notes on alternate delivery of case-based content below.

**Facilitators**

For our session, facilitators consisted of two pediatricians with a background in medical education and trauma-informed care. The number of facilitators can vary to accommodate the size of the participant group. We recommend that facilitators for this session have a basic knowledge of trauma-informed care. We also recommend that facilitators are familiar with the clinical setting in which the audience members see patients, as the case-based elements may generate specific recommendations/discussions that facilitators may have to navigate.

**Timeline of Session**

Total time: 1 hour

From Appendix B: Module Slide Set (includes presenter notes)

1. Introduction/objectives: Slides 1-2 (2 minutes)
2. Background and review of trauma, adverse childhood experiences, and concepts of trauma-informed care: Slides 3-13 (10 minutes)

*Open to participant questions to ensure clarity (2 minutes)*

1. Patient cases- initial review: Slides 14-27 (15 minutes)
2. Trauma-informed clinical practice: Slides 28-41 (10 minutes)

*Open to participant questions to ensure clarity (3 minutes)*

1. Patient cases- “Putting It Into Practice”: Slides 42-45 (13 minutes)
2. Closing/questions: Slides 46-48 (5 minutes)

**Facilitator Notes on Cases and Case Delivery**

These notes reflect how the module was delivered virtually without the use of breakout sessions. There are also suggestions for alternate delivery methods below.

The first time that the cases were presented, we used the chat feature in our online platform and encouraged participants to call out responses in a “popcorn” fashion to solicit input from participants.

- One of the facilitators tracked the responses, both verbal responses and those typed into the chat feature, and read them out to the group to help facilitate discussion.
- On the review slides, a facilitator highlighted some of the common themes from the discussion that occurred during the case slide and affirmed participant experiences.

During the “Putting It Into Practice” section of the module (slides 42-45), we used *PollEverywhere* to allow for free-text responses that could be “voted” up or down by participants, as well as to allow for interactive discussion in real time.

- Examples of some potential ways to put the principles into practice in each scenario as well as potential barriers are included in the presenter notes of the slide set.

**Alternate Delivery of Case-Based Content**

As indicated above, this session can be adapted to optimize participant engagement and to allow for in-person delivery.

- For delivery with breakout sessions, more time will be needed to allow for breaking into small groups and reassembling as well as for “report out” of the small group discussions to the larger reassembled group. We suggest 20 minutes total (inclusive of the time to review the cases) for each breakout session. This may be done with both case presentation sections of the module or just the “Putting It Into Practice” component at the end.
- An adequate number of facilitators is needed for each small group. Facilitators should track participant responses to aid in “report out” to the larger group.
- In-person delivery space should be laid out to optimize engagement and allow for breakout sessions if desired.
